# Genome scans capture key adaptation and historical hybridization signatures in tetraploid wheat

**DOI:** 10.1002/tpg2.20410

**Published:** 2023-11-16

**Authors:** Demissew Sertse, Jemanesh K. Haile, Ehsan Sari, Valentyna Klymiuk, Amidou N'Diaye, Curtis J. Pozniak, Sylvie Cloutier, Sateesh Kagale

**Affiliations:** ^1^ Aquatic and Crop Resource Development National Research Council Canada Saskatoon Saskatchewan Canada; ^2^ Crop Development Centre University of Saskatchewan Saskatoon Saskatchewan Canada; ^3^ Department of Microbiology and Plant Pathology University of California Riverside California USA; ^4^ Ottawa Research and Development Centre Agriculture and Agri‐Food Canada Ottawa Ontario Canada; ^5^ Department of Plant Science Faculty of Agricultural and Food Sciences University of Manitoba Winnipeg Manitoba Canada; ^6^ Canola Council of Canada Crop Production and Innovation Saskatoon, SK Canada

## Abstract

Tetraploid wheats (*Triticum turgidum* L.), including durum wheat (*T. turgidum* ssp*. durum* (Desf.) Husn.), are important crops with high nutritional and cultural values. However, their production is constrained by sensitivity to environmental conditions. In search of adaptive genetic signatures tracing historical selection and hybridization events, we performed genome scans on two datasets: (1) Durum Global Diversity Panel comprising a total of 442 tetraploid wheat and wild progenitor accessions including durum landraces (*n* = 286), domesticated emmer (*T. turgidum* ssp*. dicoccum* (Schrank) Thell.; *n* = 103) and wild emmer (*T. turgidum* ssp*. dicoccoides* (Korn. ex Asch. & Graebn.) Thell.; *n* = 53) wheats genotyped using the 90K single nucleotide polymorphism (SNP) array, and (2) a second dataset comprising a total 121 accessions of nine *T. turgidum* subspecies including wild emmer genotyped with >100 M SNPs from whole‐genome resequencing. The genome scan on the first dataset detected six outlier loci on chromosomes 1A, 1B, 3A (*n* = 2), 6A, and 7A. These loci harbored important genes for adaptation to abiotic stresses, phenological responses, such as seed dormancy, circadian clock, flowering time, and key yield‐related traits, including pleiotropic genes, such as *HAT1, KUODA1, CBL1*, and *ZFN1*. The scan on the second dataset captured a highly differentiated region on chromosome 2B that shows significant differentiation between two groups: one group consists of Georgian (*T. turgidum* ssp. *paleocolchicum* A. Love & D. Love) and Persian (*T. turgidum* ssp. *carthlicum* (Nevski) A. Love & D. Love) wheat accessions, while the other group comprises all the remaining tetraploids including wild emmer. This is consistent with a previously reported introgression in this genomic region from *T. timopheevii* Zhuk. which naturally cohabit in the Georgian and neighboring areas. This region harbored several adaptive genes, including the thermomorphogenesis gene *PIF4*, which confers temperature‐resilient disease resistance and regulates other biological processes. Genome scans can be used to fast‐track germplasm housed in gene banks and in situ; which helps to identify environmentally resilient accessions for breeding and/or to prioritize them for conservation.

## Core Ideas

- Genome scans were performed on two datasets: 90K single nucleotide polymorphism array of Durum Global Diversity Panel and whole‐genome resequencing data of tetraploid wheat.
- Genome scans on both datasets detected outlier loci harboring genes with essential roles in adaptation.
- Genome scan on the second dataset revealed a likely introgression, highly differentiated region on chromosome 2B.
- Genome scans can help to fast‐track germplasm in gene banks and in situ for breeding and conservation purposes.

**Abbreviations**
CIMMYT: International Maize and Wheat Improvement Center; FHB, Fusarium head blight
GDP: Durum Global Diversity Panel
GEA: genome–environment association
GOC: gene order conservation
LD: linkage disequilibrium
NJ: neighbor joining
PC: principal component
PCA: principal component analysis
SNP: single nucleotide polymorphism
UN: United Nations
USD: United States dollar
WG: whole genome

## 1 INTRODUCTION

To cope with the current climate change‐related challenges in food and feed production for the growing world population, developing a wide range of environment‐resilient or niche‐specific cultivars has become a key consideration in plant breeding schemes. Understanding the genomic regions underlying adaptations to different environmental conditions in crops is advantageous. Genetic signatures that pinpoint genomic records of historical selection, hybridization, and other evolutionary events provide vital information for designing or tailoring crop breeding methods toward achieving targeted outcomes.

The tetraploid domesticated emmer wheat (*Triticum turgidum* ssp. *dicoccum*) was one of the crops cultivated at the beginning of agriculture ∼10k years ago in the Fertile Crescent, seemingly in the present Syria (Matsuoka, 2011). Given that hexaploid wheat likely evolved from domesticated emmer wheat (Dvorak et al., 2012), tetraploid wheat cultivation must have predated the advent of hexaploid wheat. Tetraploid wheat exists both as hulled and free‐threshing types, where the two were believed to have evolved from two major post‐domestication gene pools and spread into separate geographic regions (Oliveira et al., 2012). Free‐threshing durum wheat (*T. turgidum* ssp. *durum*) currently predominates all tetraploid wheat cultivating regions and is nearly the sole tetraploid wheat produced in recent times (Laidò et al., 2013).

Durum wheat is an important crop that is well adapted to the Mediterranean and other semi‐arid environmental niches found in Canada, Russia, and West Asia (Ceglar et al., 2021). Historically, durum wheat represented a substantial proportion of wheat production, covering the vast old wheat‐growing regions spanning the high‐latitude temperate zones of Eurasia in the north to the low‐latitude Abyssinian highlands and sub‐Saharan oases in the south (Sall et al., 2019). The crop is assumed to have diverged across these historical habitats.

Durum wheat is a crop with many nutritional and other essential grain qualities (Nazco et al., 2014). As a result, it is primarily used for high‐quality food production (Nazco et al., 2014; Sarkar & Dexter, 2016). Traditional consumers use the grain to make many types of food (Abecassis et al., 2018; Hammami & Sissons, 2020). It is a staple grain in most Mediterranean, West Asian, and some sub‐Saharan African countries (Hammami & Sissons, 2020; Sall et al., 2019). Currently, more than six million tons of durum wheat, worth ∼USD 1.8 billion, are traded in the international market annually; Canada and Senegal were the lead exporter and importer in 2019, respectively (UNData, 2020). Most of the European production, including the Mediterranean region, is consumed domestically (Finch et al., 2014). Nearly all of the durum wheat produced is used for human consumption, unlike most other cereals, including common wheat, which are also used as animal feed and biofuel (Beres et al., 2020).

Despite its high nutritional and cultural relevance, durum wheat suffers from diseases such as Fusarium head blight (FHB) and adaptation to narrow environmental ranges (Martínez‐Moreno et al., 2022). These have been exacerbated by the increasing abiotic and biotic stresses associated with climate change that have impeded genetic improvements in some regions (Beres et al., 2020; Hochman & Horan, 2018). Breeding and its associated practices have resulted in the development of elite cultivars that have successfully spread into several durum wheat‐growing regions (Xynias et al., 2020). The spread of elite cultivars, however, contributes to the erosion of landraces that are well adapted to their specific niches and consequently decreases genetic variation for adaptive traits (Van de Wouw et al., 2010). A large number of the contemporary cultivars grown in many countries were developed by International Maize and Wheat Improvement Center (CIMMYT) and its collaborators, primarily through cross‐breeding among limited elite germplasm. Many cultivars originate from the same lineage and as a consequence, their coefficient of parentage is high posing potential genetic bottlenecks in the breeding gene pool of the crop (Pfeiffer et al., 2000; Xynias et al., 2020). To revise and improve the genetic landscape of the current breeding germplasm, it is essential to explore the missed variations available in landraces and wild relatives that potentially harbor important genetic features underlying traits of interest. To capture genetic signatures of historical selections, hybridizations and adaptations, we performed genome scans of durum wheat landraces (*T. turgidum* ssp. durum), domesticated emmer (*T. turgidum* ssp. *dicoccum*), and wild emmer (*T. turgidum* ssp. *dicoccoides*) wheat accessions from the Durum Global Diversity Panel (GDP) (Mazzucotelli et al., 2020). To trace further hybridization and/or evolutionary events, additional tetraploid wheat genomes based on high‐density markers obtained from whole‐genome resequencing data extracted from Zhou and Yang et al. (2020) were also scanned.

## 2 MATERIALS AND METHODS

The Illumina Infinium 90K single nucleotide polymorphism (SNP) data of the GDP (*n* = 1011) previously published by Mazzucotelli et al. (2020) was downloaded from https://wheat.pw.usda.gov/GG3/global_durum_genomic_resources along with the passport data of the accessions. Considering the high rate of cultivar exchanges across the globe that may undermine the actual adaptation to environmental conditions and, with the assumption that local landraces and wild relatives better reflect historical selections, hybridizations and adaptations, we analyzed only the landrace and emmer wheat subsets of the GDP. Based on their passport information, a total of 442 accessions were extracted from the GDP: 286 durum landrace accessions as well as 103 domesticated and 53 wild emmer wheat accessions (Table S1).

The tetraploid wheat population structure and genetic variations were well studied using the data comprising the full set of the Global Tetraploid Wheat Collection (GTC) (Maccaferri et al., 2019) and GDP (Mazzucotelli et al., 2020). Here we performed similar analyses to specifically get insights into the population structure and genetic variation among populations of the selected subsets.

To define the optimum number of ancestral subpopulations (K), eigenvalues were computed for 30 principal components (PCs) and visualized into scree plots using principal components to detect local adaptation (PCADAPT) (Luu et al., 2017). Following the Cattell's rule suggested by the PCADAPT authors, the PC to the left of the last curve of a horizontal trend was selected as the appropriate number of ancestral subpopulations (Cattell, 1966; Luu et al., 2017).

To estimate the individual ancestral coefficients, a non‐negative matrix factorization (sNMF) (Kim & Park, 2007) was applied using the sNMF function (Frichot et al., 2014) implemented in the R package landscape and ecological association (LEA) (Frichot & François, 2015) at the determined *K* subpopulations. Accessions were assigned to subpopulations according to their percentage of ancestry and visualized as bar plots using the same package. Principal component analysis (PCA) was also performed for *K* PCs. The clustering of the accessions assigned to the subpopulations was visualized based on the first three PCs, and the percent of the variation explained by each PC was calculated. To further observe the genetic relationship among the individuals, a neighbor‐joining (NJ) phylogenetic analysis was performed in TASSEL 5 (Bradbury et al., 2007), and phylogenetic trees were visualized using an interactive tree of life (iTOL) (Letunic & Bork, 2016).

**Core Ideas**

- Genome scans were performed on two datasets: 90K single nucleotide polymorphism array of Durum Global Diversity Panel and whole‐genome resequencing data of tetraploid wheat.
- Genome scans on both datasets detected outlier loci harboring genes with essential roles in adaptation.
- Genome scan on the second dataset revealed a likely introgression, highly differentiated region on chromosome 2B.
- Genome scans can help to fast‐track germplasm in gene banks and in situ for breeding and conservation purposes.

To estimate the genetic variation among the subpopulations, F‐statistics were estimated based on *F*_ST_ (Weir & Cockerham, 1984) and *G*″_ST_ (Meirmans & Hedrick, 2011) using the R package diveRsity (Keenan et al., 2013). To estimate the genetic diversity of subpopulations, both inter‐ and intra‐genetic diversities were estimated based on mean square allele size differences from GENEPOP v4.7.5 (Rousset, 2008) in R (Rousset et al., 2020).

To capture the outlier loci posited to be genetic signatures hinting at historical selection and evolutionary events, a genome scan based on PCADAPT at *K* PCs was performed, and the *p‐*value of each marker was obtained using PCADAPT v4 (Luu et al., 2017; Privé et al., 2020). A significant threshold *α* = 0.05 was set, and Bonferroni correction (*α*/*n*, *n* = number of SNPs) was applied to control the cumulative type I errors resulting from the large numbers of comparisons. Results were visualized in Manhattan plots. The outlier markers/loci with a significant Bonferroni‐corrected *p*‐value were considered as potential adaptive signatures.

To discern the variations based on the outlier loci, both multi‐locus and single‐locus haplotype analyses were performed. The multi‐locus haplotypes were constructed based on the alleles at the outlier markers whereas the single‐locus haplotypes at each outlier SNP were obtained from markers in strong linkage disequilibrium (*r* ^2^> 0.64) with the outlier SNP. The country of origin of the accessions was obtained from their passport information. The haplotype frequencies for the domesticated emmer, landraces, and wild emmer were calculated. The geographic distributions of the haplotypes were also shown as pie charts displayed on a world map created using georeferenced coordinates of the country of origin obtained with Quantum Geographic Information System (QGIS) from the Open‐Source Geospatial Foundation Project (http://qgis.osgeo.org). The pie chart's sizes were proportionally scaled to the number of accessions carrying the depicted haplotypes.

To identify genes linked to the detected markers/loci, genes and their coordinates were extracted from the reference genome annotation of durum wheat cv. Svevo (Maccaferri et al., 2019). Linkage disequilibrium analysis using the genotypic data was performed using the R package gpart (Kim et al., 2019). Linkage disequilibrium blocks (LDBlocks) that contained outlier loci were identified (*r* ^2^ *>* *0.64*), and all the genes within these blocks were investigated for their potential role based on their orthologs in *Triticum aestivum* and other well‐studied species, such as *Arabidopsis thaliana*, as annotated in Ensembl Plants (https://plants.ensembl.org). Orthologs of *T. aestivum* with gene order conservation (GOC) score ≥50 were selected, and their functions were investigated using the KNETMINER database (https://knetminer.com). Genes with potential adaptive functions were summarized and positioned onto cv. Svevo reference genome.

To capture additional signatures of historical selection and hybridization as well as other evolutionary events, we used a high‐density whole‐genome resequencing dataset of tetraploid accessions of wheat and their wild ancestors from the Genome Variation Map repository (http://bigd.big.ac.cn/gvm). This 100 M SNP dataset was filtered to ∼110K using a 97% SNP call rate and >5% minor allele frequency (MAF). We then performed a genome scan with the ∼110K SNPs for 121 accessions of tetraploid wheat and their wild relatives (Table S3), each accession having no more than 10% missing SNPs.

## 3 RESULTS

### 3.1 Population structure

**FIGURE 1:**
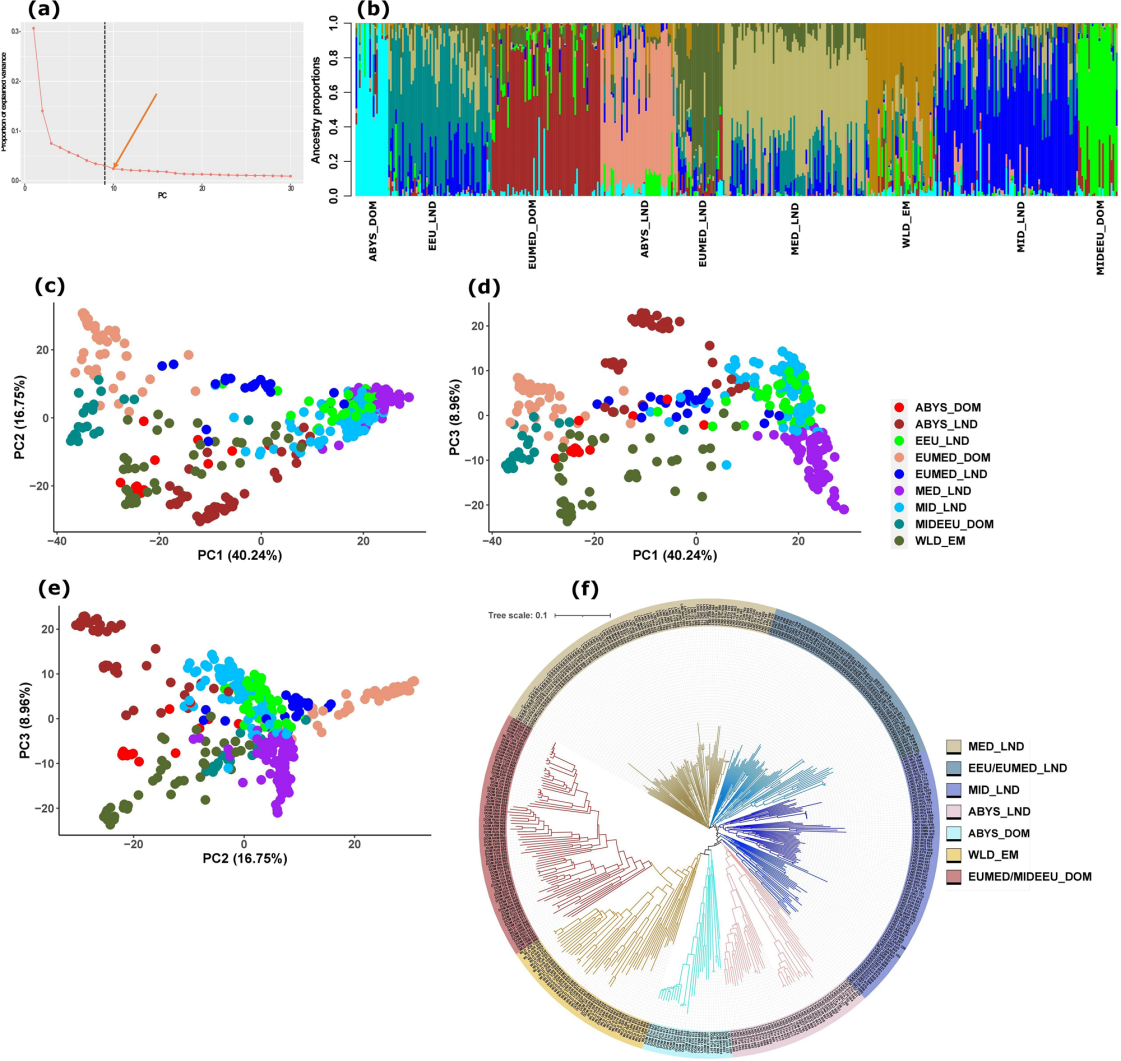
Population structure of 286 durum landraces,103 domesticated, and 53 wild emmer wheat accessions of the Durum Global Diversity Panel. (a) Principal component (PC)‐based scree plot showing the estimated number of subpopulations indicated by the PC (PC = 9) at the broken vertical line to the left of the last curve toward horizontal as indicated by the orange arrow (PC = 10), (b) bar plots showing the level of admixture for each accession, (c–e) plots of the first three PCs (PC1, PC2, and PC3) and percent variation explained by each, (f) neighbor‐joining (NJ) phylogenetic tree where each clade is colored differently and accession names are indicated at the end of each branch. Accession names comprise the cultivation status (DOM, domesticated emmer; LND, durum landrace; WLD, wild emmer), the United Nations three‐letter country code of origin and the accession number.

The entire population (*n* = 442) was subdivided and assigned to nine subpopulations (Figure 1a and Table S1). The highest number of individuals assigned to a subpopulation was dominated by Mediterranean landrace accessions (MED_LND). Accessions within this subpopulation were noticeably admixed with the subgroups dominated by Eastern European (EEU_LND) and Middle Asian (MID_LND) landraces. The subgroup that harbored the wild emmer wheats (WLD_EM) was fairly admixed with Abyssinian (ABYS_DOM) and Middle East–Eastern European (MIDEEU_DOM) domesticated emmer wheat accessions. The ABYS_DOM and MIDEEU_DOM shared little ancestry, and both appeared to be relatively pure subpopulations. A noticeable number of accessions under the subgroup European–Mediterranean domesticated (EUMED_DOM) also included high ancestral proportion from MIDEEU_DOM. However, MIDEEU_DOM was nearly absent in Mediterranean landraces (MED_LND) (Figure 1b and Table S1).

EUMED_DOM and MIDEEU_DOM consistently overlapped, as displayed in the PC1 versus PC2 and PC1 versus PC3 plots (Figure 1c,d) as well as in the NJ phylogenetic tree (Figure 1f). Together, these subgroups occupied a clade with the longest branch in the NJ tree. Consistent with the results of the admixture analysis, WLD_EM overlapped with ABYS_DOM in PCA (Figure 1c–e); this relationship is also reflected in the NJ tree where the two subpopulations are distinct but closely related (Figure 1f). The accessions of the Mediterranean landrace (MED_LND) subgroup clustered together in both PCA plots and phylogenetic NJ tree. They occupied a clade with short and less subdivided branches. Generally, the five landrace subgroups were depicted by short and less branched clades. Among them, the ABYS_LND displayed the longest branches (Figure 1f).

### 3.2 Genetic variation and diversity

The genetic variation pattern among the population exhibited similarity in both genome‐wide (Table 1) and SNP‐level analyses when based on *F_ST_* and G’’_ST_. (Figure S1). The highest genetic differentiation (*F*_ST_ = 0.55) was computed between MIDEEU_DOM and MED_LND, followed by ABYS_DOM and MED_LND (*F*_ST_ = 0.52), while the lowest (*F*_ST_ = 0.16) was between MED_LND and MID_LND (Table 1). The WLD_EM was more closely related to the Abyssinian and Middle Eastern subgroups than to their Mediterranean and European counterparts. The highest genetic variation (*F*_ST_ = 0.38) for the WLD_EM subpopulation was estimated with the MED_LND, which also showed a consistently higher variation with both the domesticated and wild emmer wheats. The WLD_EM harbored the highest genetic diversity (*D* = 0.33), followed by EUMED_LND (*D* = 0.30), while the lowest *D* = 0.19 and 0.21 were obtained for ABYS_DOM and MIDEEU_DOM, respectively. This was also reflected in the admixture analysis, where these latter two subgroups appeared to be relatively pure, with little admixture.

**TABLE 1:** Genetic variation and inter‐individual genetic diversity of cultivated durum landraces, domesticated, and wild emmer wheat accessions of the Durum Global Diversity Panel allocated to their subpopulations (Figure 1), where the diagonal values are the gene diversity, the values above the diagonal are the *F*_ST_ (Weir & Cockerham, 1984) and those below are the *G*”_ST_ (Meirmans & Hedrick, 2011).

Subpopulation	ABYS_DOM	ABYS_LND	EEUR_LND	EUMED_DOM	EUMED_LND	MED_LND	MID_LND	MIDEEU_DOM	WLD_EM
ABYS_DOM	0.19	0.37	0.46	0.47	0.41	0.52	0.41	0.48	0.29
ABYS_LND	0.53	0.29	0.33	0.43	0.28	0.39	0.28	0.41	0.24
EEUR_LND	0.63	0.47	0.27	0.45	0.23	0.17	0.09	0.48	0.32
EUMED_DOM	0.61	0.57	0.60	0.22	0.32	0.51	0.42	0.35	0.34
EUMED_LND	0.57	0.41	0.33	0.42	0.30	0.28	0.21	0.40	0.25
MED_LND	0.68	0.52	0.23	0.66	0.37	0.22	0.16	0.55	0.38
MID_LND	0.59	0.41	0.13	0.58	0.31	0.22	0.29	0.44	0.29
MIDEEU_DOM	0.61	0.57	0.65	0.46	0.56	0.72	0.63	0.21	0.29
WLD_EM	0.44	0.36	0.46	0.46	0.39	0.52	0.42	0.43	0.33

Abbreviations: ABYS, Abyssinian; DOM, domesticated, EEUR, East European; EM, Emmer; EUMED, European and Mediterranean mixed; LND, landraces; MED, Mediterranean; MID, Middle Eastern; MIDEEU, Middle Eastern and Eastern Europe mixed; WLD, wild.

### 3.3 The haplotypes from the genomescan detected loci

**FIGURE 2:**
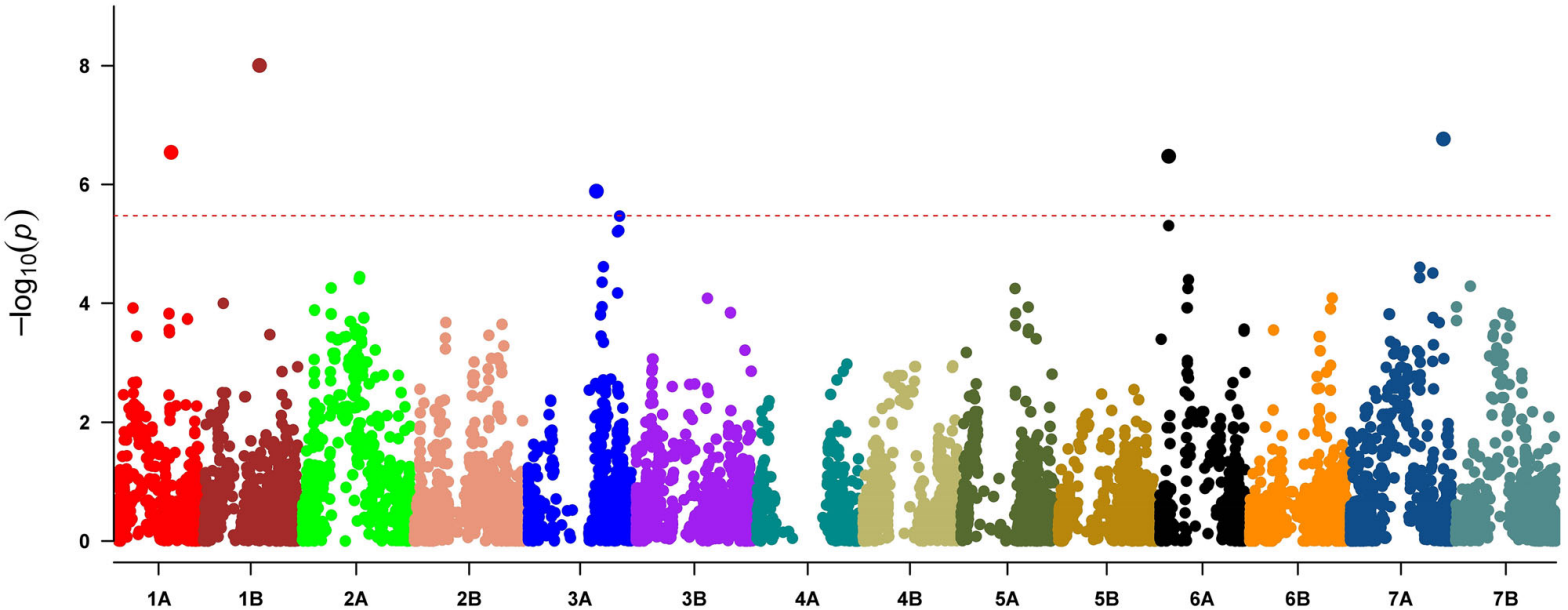
Manhattan plot showing the six outlier loci representative of the adaptive genetic signatures. The horizontal dotted line indicates the significance threshold after Bonferroni correction (*α*/*n* = 0.05/*n*, where *n* = number of markers). The colors represent the chromosomes of tetraploid wheat as denoted on the *x* axis. Three outlier markers are superimposed on chromosomes 3A and 6A.

The genome scan of the GDP captured a total of ten outlier markers (Table 2) at six loci on chromosomes 1A, 1B, 3A (*n* = 2), 6A, and 7A (Table 2 and Figure 2). Loci and/or LDBlocks (*r* > 0.8) on chromosomes 3A and 6A, each harbored three outlier markers. The locus on 1B spanned a large 5 Mbp block, and comprised the most genes (Table 2).

**TABLE 2:** Chromosomal location and *p* values of adaptive loci inferred from genome scan analysis, linkage disequilibrium blocks (LDBlocks), and number of genes.

Marker	**Chr**	Position	*p* **values**	LDBlock (Locus)	No. genes
Excalibur_c16292_1338	1A	382853977	2.9E‐07	BobWhite_c4942_140 ‐ GENE‐0491_632 (380188147‐ 382858874)	19
Tdurum_contig10955_429	1B	403564038	9.9E‐09	Tdurum_contig10955_429 ‐ BobWhite_c11460_291 (403564038 −408577880)	21
BS00090405_51	3A‐1^a^	490120631	1.3E‐06	BS00090405_51 ‐ BS00110452_51 (490120631 −490513609)	2
IACX5968	3A‐1^a^	490120866	1.3E‐06		
BS00110452_51	3A‐1^a^	490513609	1.3E‐06		
Tdurum_contig45539_226	3A‐2	657352397	3.4E‐06	BobWhite_c801_862 ‐ RAC875_c79551_167 (654788785 ‐ 657600168)	17
BS00090928_51	6A^a^	64002219	3.6E‐07	BS00090928_51 ‐ wsnp_Ex_c25300_34566908 (64002219 −64947345)	9
wsnp_Ex_c55340_57883479	6A^a^	64382573	3.6E‐07		
wsnp_Ex_c25300_34566908	6A^a^	64947345	3.6E‐07		
BS00030391_51	7A	668807476	1.7E‐07	Excalibur_c14451_389 ‐ wsnp_CAP7_c1321_664480 (668541326‐ 669297487)	9

Abbreviations: Chr, chromosome; LDBlock, linkage disequilibrium block defined by lower and upper boundary marker; No. genes, number of genes harbored within the LDBlocks.

^a^ Markers within the same LDBlock (*r* ^2^ > 0.64).

**FIGURE 3:**
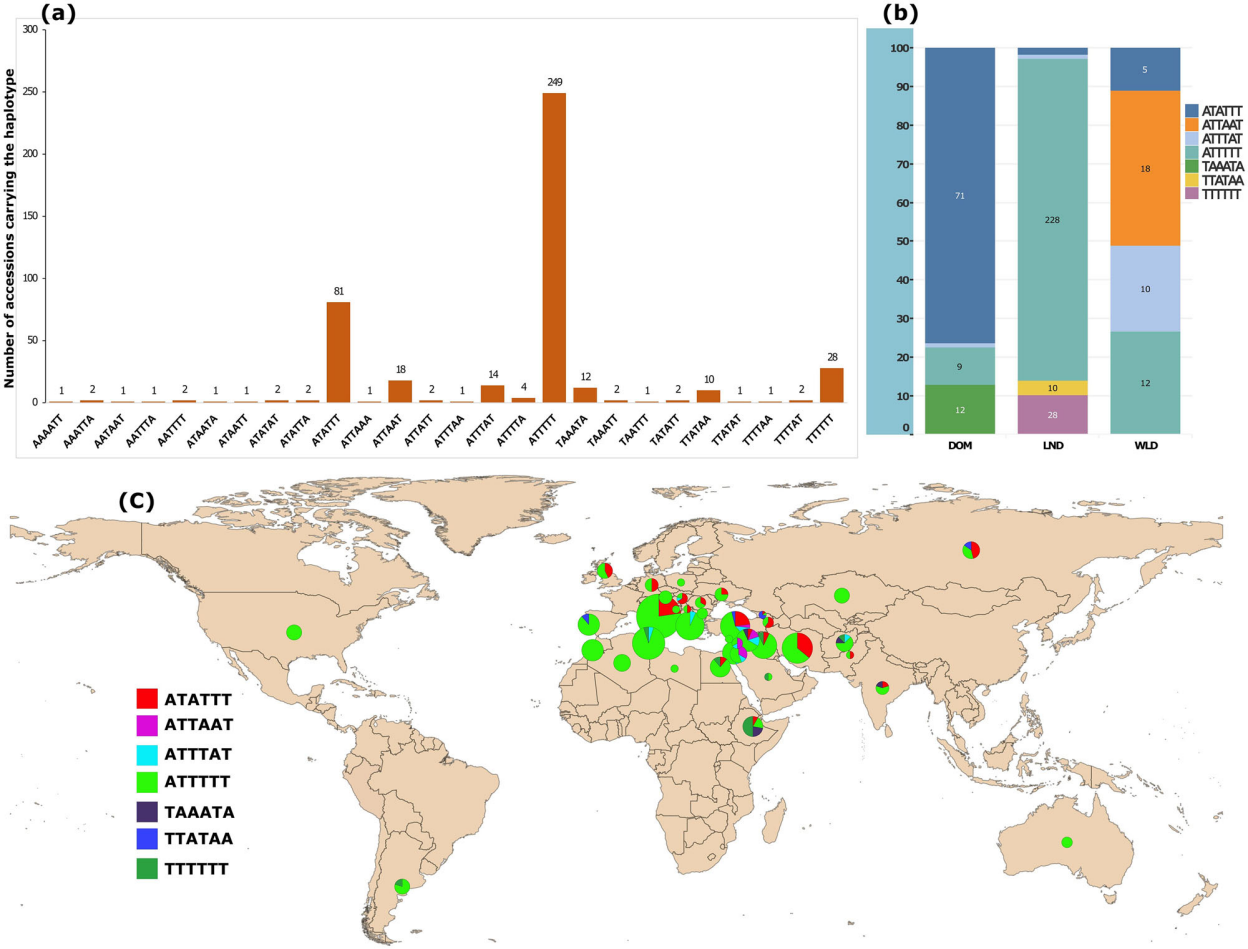
Frequency and geographic distribution of multi‐locus haplotypes. (a) Number of individuals carrying each of the 26 haplotypes defined by the single nucleotide polymorphisms at the six outlier loci identified by genome scan, (b) the frequencies of the haplotypes in domesticated emmer, landrace, and wild emmer wheat types, and (c) geographic distribution of the seven most frequent haplotypes. The size of the pie is proportional to the number of accessions. Each color in the pie chart represents one of the seven most frequent haplotypes as described in the legend.

Based on the SNPs at the six loci, the 442 accessions could be assigned to 26 multi‐locus haplotypes, of which ten were represented by a single accession, eight by two accessions, and one by four accessions. The remaining seven haplotypes were represented by at least ten accessions, and the most frequent haplotype was present in 249 accessions (Figure 3a). The seven most frequent haplotypes, assigned to domesticated emmer, landrace and wild emmer types (Figure 3b), were overlaid onto a world map based on the geographic coordinates of the countries from which the accessions originated (Figure 3c). The most frequently observed haplotype, “ATTTTT”, was predominately carried by the landraces (Figure 3b) and did not show a distinct geographic distribution as can be noted by its occurrence in nearly all countries of origin (Figure 3c, green). However, the second most frequent haplotype, “ATATTT,” was mostly restricted to Europe and Middle East Asia (Figure 3c, red) and mainly present in domesticated emmer accessions (Figure 3b). This haplotype was observed at a reasonably high frequency among Italian and Turkish accessions, but it was absent in most other Mediterranean countries. In contrast, haplotype “ATTTAT” was mostly limited to the Mediterranean environment, although it was seen as far east as Afghanistan (Figure 3c, light blue). This haplotype was observed mainly in wild emmer accessions. The haplotype “ATTAAT,” exclusively harbored by wild emmer wheats, was distributed in a confined region represented by the neighboring countries of Israel, Jordan, Lebanon, Syria, and Turkey (Figure 3c, magenta). Haplotype “TTTTTT,” present in nearly half of the Abyssinian accessions, was also observed in Argentina (Figure 3c, dark green). Other than the most frequent haplotype, this was the only haplotype observed in the New World. This haplotype was observed only in the landraces (Figure 3b). The other two haplotypes (dark purple and dark blue) were scattered over vast and diverse geographic regions. No private/endemic haplotype was observed for any of the countries.

The locus‐specific analysis based on markers in strong linkage disequilibrium (*r* ^2^> 0.64) at each locus yielded 42 single‐locus haplotypes (Table 3 and Figure S2), of which 30 were present in at least five accessions in a single group (domesticated emmer, landraces, or wild emmer) (Table 3). Haplotype “ATTA” at Chr 6A:4002219–64947345 was the most frequent, being observed in 391 of the 442 accessions. This haplotype was carried by 99% of the domesticated emmer and 94% of the landraces. Haplotypes “TTAT” at Chr1A: 380188147–382858874 and “TAAT” and “TTAT” at Chr 3A: 654788785–657600168 were endemic to the landrace and wild emmer groups, respectively (Table 3 and Figure S2).

**TABLE 3:** Locus‐specific haplotypes and their distribution among the domesticated emmer, landrace, and wild emmer wheat types.

Locus	Haplotype block	Hap	Distribution among the wheat types					
			Count			Percentage		
			DOM	LND	WLD	DOM	LND	WLD
**Hap1A**	Chr1A: 380188147–382858874	AAAA	13	2	2	12.6	0.7	3.8
		AAAT	1	5	19	1.0	1.7	35.8
		AATT	15	43		14.6	15.0	0.0
		ATAA	42	17	9	40.8	5.9	17.0
		ATAT	2	3		1.9	1.0	0.0
		TAAA	1	2	2	1.0	0.7	3.8
		TAAT	29	193	21	28.2	67.5	39.6
		TATT	1			1.0	0.0	0.0
		TTAT^a^		20		0.0	7.0	0.0
**Hap1B**	Chr1B: 403564038–408577880	AAATA	4	2		3.9	0.7	0.0
		AATAT	14	2		13.6	0.7	0.0
		AATTA	1			1.0	0.0	0.0
		ATTTA			1	0.0	0.0	1.9
		TAATA			4	0.0	0.0	7.5
		TATAA			1	0.0	0.0	1.9
		TATAT	19	274	14	18.4	95.8	26.4
		TATTA		7	25	0.0	2.4	47.2
		TTTTAD	65	1	8	63.1	0.3	15.1
**Hap3A1**	Chr3A: 490120631–490513609	ATAAL	6	42	20	5.8	14.7	37.7
		ATTAL	5	228	25	4.9	79.7	47.2
		TATT	92	16	8	89.3	5.6	15.1
**Hap3A2**	Chr3A: 654788785–657600168	AATA	29	103	11	28.2	36.0	20.8
		AATT	53	40	12	51.5	14.0	22.6
		TAAT^a^			6	0.0	0.0	11.3
		TATA		4	1	0.0	1.4	1.9
		TTAA	17	2		16.5	0.7	0.0
		TTAT^a^			16	0.0	0.0	30.2
		TTTA	4	130	6	3.9	45.5	11.3
		TTTT		7	1	0.0	2.4	1.9
**Hap6A**	Chr6A: 64002219–64947345	ATTA	102	268	21	99.0	93.7	39.6
		TAAT	1	18	31	1.0	6.3	58.5
		TATT			1	0.0	0.0	1.9
**Hap7A**	Chr7A: 668541326–669297487	AAAA	2	73	3	1.9	25.5	5.7
		AATA	3	66	4	2.9	23.1	7.5
		AATT	5	168	7	4.9	58.7	13.2
		ATTT	10	2	1	9.7	0.7	1.9
		TAAA	7	12		6.8	4.2	0.0
		TATA	1	2		1.0	0.7	0.0
		TATT	10	31	7	9.7	10.8	13.2
		TTAA	10	1	1	9.7	0.3	1.9
		TTAT			2	0.0	0.0	3.8
		TTTT		2	31	0.0	0.7	58.5

Abbreviation: LDBlock, linkage disequilibrium block (interval) at each locus on chromosomes.

^a^ Haplotype observed only in one wheat type.

Country‐specific haplotypes were not identified (Figure S3a–f). However, haplotypes “AAAT” at Chr1A:380188147–382858874, “TATTA” at Chr1B:403564038–408577880, and “TTAT” at Chr3A:654788785–657600168 were largely confined to the Mediterranean region (Figure S3a,b,d). These haplotypes were predominant in wild emmer accessions (Table 3). Haplotypes “AATT” at Chr1A: 380188147–382858874, “AATAT” at Chr1B:403564038–408577880, and “TTAA” at Chr3A:654788785–657600168 and Chr7A:668541326–669297487 were noticeably more frequent in Abyssinian accessions, while they were rarely observed in other geographic regions (Figure S3a,b,d,f).

### 3.4 Candidate genes

Based on the cv. Svevo gene annotation (Table 2), a total of 77 genes were in LD (*r* ^2^ *= 0.64*) with the detected markers. Based on their strong orthologs in *T.aestivum*, with GOC > 50%, with most being 90% and above, the majority of these genes in have key function(s) in important biological processes involved in the adaptation of plants to environmental conditions, and some could be hypothesized to affect traits that could conceivably be targeted in anthropogenic selections. Of the 77 genes, 30 were involved in regulating important adaptive or selection target traits such as seed dormancy, circadian rhythm, phenological traits (e.g., days to heading, flowering, and maturity), grain yield‐related traits, disease resistance and response to abiotic stresses, including drought, salt, cold, and heat (Table 4).

**TABLE 4:** Genes located within LDBlocks (*r* ^2^ > 64) of the six outlier loci identified by principal component analyses of the Durum Global Diversity Panel that play roles in adaptation and/or as selection targets.

*Triticum turgidum* gene ID	*T*riticum *aestivum* ortholog	Chr	Sv_Start	CS_Start	Gene name	Role
*TRITD1Av1G140940*	*TRAESCS1A02G219200*	1A	380535963	387840338	*HAT1*	1Sdr; Cr; 2D; S; 3*; 4
*TRITD1Av1G141030*	*TRAESCS1A02G219400*	1A	380854610	388156746	*KUA1*	2C; 3*; 4stripe
*TRITD1Av1G141040*	*TRAESCS1A02G219500*	1A	380859106	388160270	*HEX6*	3
*TRITD1Av1G141260*	*TRAESCS1A02G220000*	1A	381559410	388849047	*DREB1F*	2S; D
*TRITD1Av1G141440*	*TRAESCS1A02G220300*	1A	381874391	389129281	*GHD7*	3; 5H
*TRITD1Av1G141680*	*TRAESCS1A02G221000*	1A	382621628	389869226	*FDH1*	2S; 3
*TRITD1Bv1G132490*	*TRAESCS1B02G229000*	1B	405063354	411153702	*BAM3*	4
*TRITD1Bv1G132660*	*TRAESCS1B02G229200*	1B	405480562	411567688	*CKB1*	2D; C; H; 3; 4;
*TRITD1Bv1G132830*	*TRAESCS1B02G229300*	1B	405709476	411821164	*CBL1*	1Sdr; Cr; 2D; S; C; 3*; 4
*TRITD1Bv1G132890*	*TRAESCS1B02G229400*	1B	405877697	411987863	*SAPK3*	2
*TRITD1Bv1G133150*	*TRAESCS1B02G229800*	1B	406728932	412858562	*MED31*	4stripe
*TRITD1Bv1G133170*	*TRAESCS1B02G229900*	1B	406766069	412895371	*PEP*	1FD; 2D; S; H
*TRITD1Bv1G133250*	*TRAESCS1B02G230100*	1B	407056128	413204282	*CYCP4‐1*	4striperust
*TRITD1Bv1G133450*	*TRAESCS1B02G230600*	1B	407952008	414161870	*CDKF‐4*	2S; 4
*TRITD3Av1G175540*	*TRAESCS3A02G263900*	3A	490512570	487853163	*RACK1A*	1Sdr; 2D; S; 3; 4
*TRITD3Av1G241410*	*TRAESCS3A02G422100*	3A	654785629	663435887	*SBP1*	1FD; MD; 2D; C; 5
*TRITD3Av1G241420*	*TRAESCS3A02G422200*	3A	654785629	663435887	*D4H*	5H
*TRITD3Av1G241510*	*TRAESCS3A02G422600*	3A	654935122	663574417	*RBG2*	2S; 3
*TRITD3Av1G242000*	*TRAESCS3A02G423200*	3A	656028986	664691550	*ZFN1*	1HD; FD; M; 2D; 3*; 4stripe
*TRITD3Av1G242470*	*TRAESCS3A02G423500*	3A	657334932	665964671	*OGR1*	3
*TRITD3Av1G242490*	*TRAESCS3A02G423600*	3A	657352035	665980620	*FLS2*	4
*TRITD3Av1G242580*	*TRAESCS3A02G423600*	3A	657490574	665980620	*FLS2*	4
*TRITD6Av1G027150*	*TRAESCS6A02G098500*	6A	64255013	65681325	*CLC‐A*	1FD; 2D; S
*TRITD6Av1G027160*	*TRAESCS6A02G098600*	6A	64257269	65797706	*CLC‐A*	1FD; 2D; S
*TRITD6Av1G027170*	*TRAESCS6A02G098700*	6A	64361409	65801743	*NUF2*	1 M; 2S
*TRITD6Av1G027180*	*TRAESCS6A02G098800*	6A	64370791	65843198	*HSFA5*	2H
*TRITD6Av1G027190*	*TRAESCS6A02G098900*	6A	64382427	65856073	*SYT5*	2D
*TRITD6Av1G027380*	*TRAESCS6A02G099100*	6A	64893281	66373343	*Rf1*	3Fertility
*TRITD7Av1G254470*	*TRAESCS7A02G484800*	7A	668819625	675587045	*RKS1*	4
*TRITD7Av1G254580*	*TRAESCS7A02G485300*	7A	669296601	676102667	*FKBP13*	3

Abbreviations: Chr, chromosome; CS_Start, start position of the gene in the Chinese Spring reference genome; Role = reported role(s) of the gene, 1 = phenological and related traits, Sdr = seed dormancy, Cr = Circadian rhythm, FD = days to flowering, HD = days to heading, MD = days to maturity, 2 = abiotic stress, C = cold, D = drought, H = heat, S = salt, 3 = yield‐related trait, * genes affecting multiple yield traits such as grain size, grain number and shattering, 4 = disease resistance, stripe = stripe rust. Sv_Start, start position of the gene in the cv Svevo reference genome. Databases such as KNETMINER (https://knetminer.com), and ENSEMBL Plants (https://plants.ensembl.org) were consulted to collect information for this table.

### 3.5 Potential signatures of historical selection and hybridization events

**FIGURE 4:**
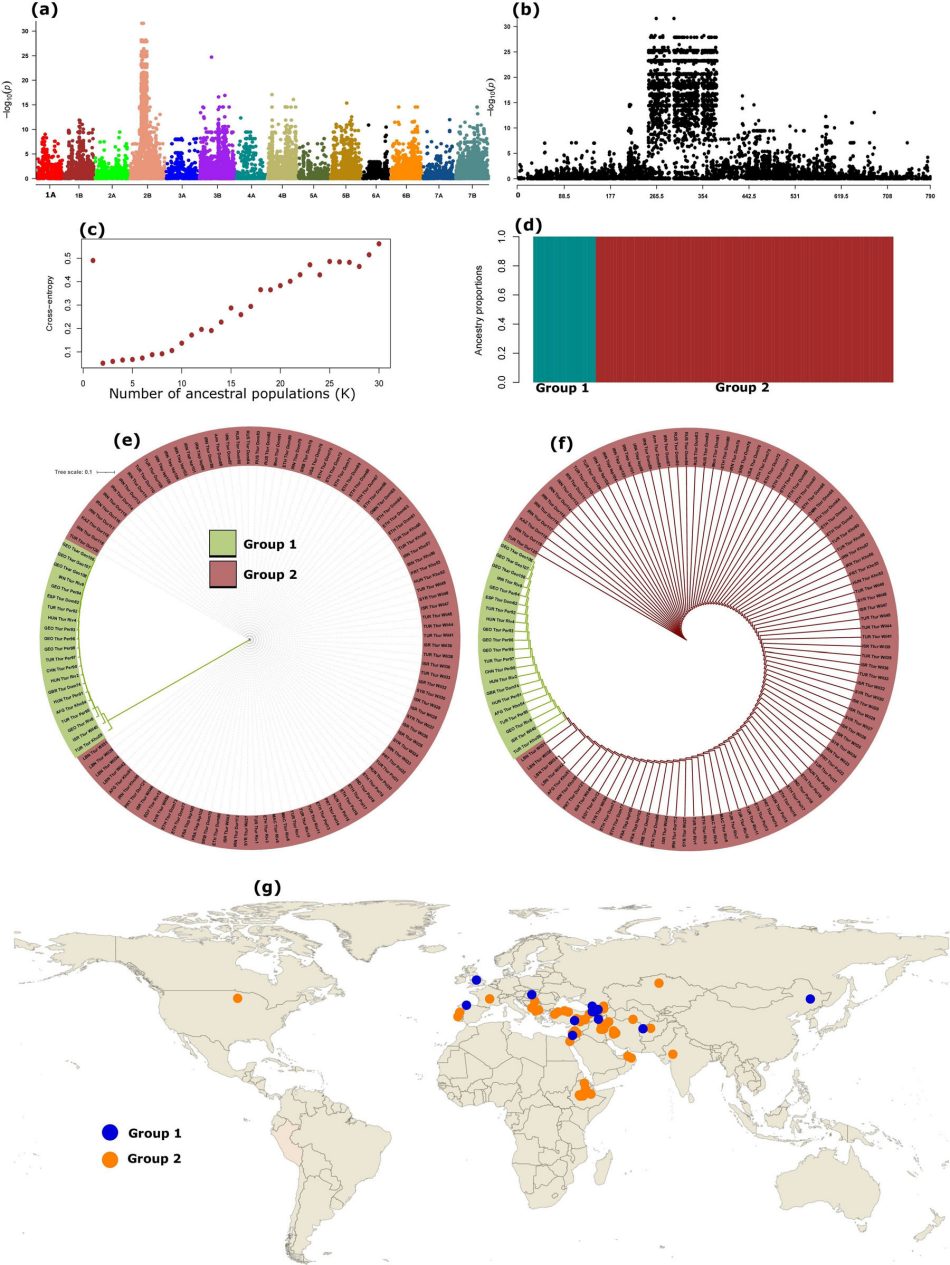
Genome scan analyses of the ∼110K SNP dataset showing a large and unique region on chromosome 2B, population structure, and geographic distribution of the accessions based on the highly differentiated 2B region. (a) Manhattan plot illustration of the genome scan showing the signature regions across the genome; (b) Manhattan plot close‐up showing the position of a ∼130Mb signature region on chromosome 2B; (c) cross‐validation plot based on the signature region of chromosome 2B indicating the appropriate (the lowest point) number of subpopulations (*n* = 2) based on this peak region; (d) bar plots showing admixture (percent ancestral coefficient) of each accession (see Table S3); (e,f) NJ phylogenetic trees based on chromosome 2B signature region indicating the evolutionary distances and topology, respectively. The accession names at the end of each branch indicate the United Nations three‐letter code of the country of origin, short species name (Tkar, *Triticum karamyschevii;* Ttur, *Triticum turgidum*), and common names with a unique number for each accession; (g) geographic distribution of the accessions according to their group assignment based on their haplotype at the chromosome 2B signature region.

To trace major selection and hybridization events along the history of tetraploid wheat, we performed a genome scan of a second dataset that contained a broad genetic diversity of tetraploid wheats including many of its subspecies. The genome scan of this dataset detected a large and uniquely differentiated segment on chromosome 2B (Figure 4a), located in the ∼250–380 Mb interval (Figure 4b). The region harbors genes that regulate biological processes, such as circadian clock, vernalization, seed dormancy and yield‐related traits (Table 5 and Table S2).

**TABLE 5:** Subset of high‐confidence genes located within the ∼130Mb signature region of chromosome 2B that have putative roles in adaptation.

Gene ID	Gene name	CS_Start^a^	Tetraploid ortholog^b^	Sv_Start	Sv_End	Role
*TRAESCS2B02G273500*	*PIF4*	374801457	*TRITD2Bv1G121430*	359292935	359294566	FL; Dis; Temp; multiple
*TRAESCS2B02G254000*	*CBT*	281872239	*TRITD2Bv1G101540*	281857170	281870847	Cr; Ds; FL
*TRAESCS2B02G267000*	*MTERF2*	359612800	*TRITD2Bv1G120740*	357463546	357467553	Cr
*TRAESCS2B02G271700*	*CIPK23*	373301321	*TRIDC2BG039280*	375684953	375689049	FL; Sdr
*TRAESCS2B02G250900*	*SPL3*	260765427	*TRITD2Bv1G096640*	260530749	260532970	FL; Sdr; Vr
*TRAESCS2B02G248700*	*CPK9*	255050988	*TRITD2Bv1G093740*	249853408	249856746	FL; Sdr
*TRAESCS2B02G266700*	*BAM1*	359310845	*TRITD2Bv1G120570*	357169560	357173307	FL
*TRAESCS2B02G264000*	*TIFY5*	356582219	*TRITD2Av1G136210*	374390107	374392768	FL
*TRAESCS2B02G256600*	*GRF9*	298324714	*TRITD2Bv1G105550*	300215014	300217004	FL
*TRAESCS2B02G253600*	*CSN7*	278435469	*TRITD2Bv1G100600*	278417998	278421880	FL; Sdr
*TRAESCS2B02G264100*	*GLO1*	356594198	*TRITD2Bv1G119070*	354414600	354416948	Cr
*TRAESCS2B02G268900*	*OEP61*	364316379	*TRITD2Bv1G125080*	369750009	369754839	FL
*TRAESCS2B02G263600*	*HEN1*	354875272	*TRITD2Bv1G118520*	352701890	352706840	FL
*TRAESCS2B02G268100*	*CRL5*	361422652	*TRITD2Bv1G125930*	372639307	372642395	FL
*TRAESCS2B02G270300*	*PSBO1*	368613668	*TRITD2Bv1G123660*	365453241	365454313	Cr
*TRAESCS2B02G267500*	*PSBP*	360481412	*TRITD2Bv1G120990*	358326425	358327385	Cr
*TRAESCS2B02G273900*	*ATPC1*	375544513	*TRITD2Bv1G094130*	251446567	251447643	Cr
*TRAESCS2B02G251400*	*pod*	263811436	*TRITD2Bv1G097410*	263821373	263822474	FL
*TRAESCS2B02G250500*	*CAR2*	260605521	*TRITD2Bv1G096540*	260371214	260372662	Sdr
*TRAESCS2B02G263000*	*EXO84A*	350298617	*TRIDC2BG037760*	357260534	357272209	FL

Abbreviations: Cr, circadian clock; CS_Start, start position of the gene in the tetraploid (Svevo) genome; Ds = disease resistance; FL, flowering time; Sdr, seed dormancy; Sv_Start, end position of the gene in tetraploid (Svevo) genome; Temp = temperature; Vr = vernalization. Databases such as KNETMINER (https://knetminer.com), and ENSEMBL Plants (https://plants.ensembl.org) were consulted to collect information for this table.

^a^ Starting position in *Triticum aestivum* (Chinese Spring RefSeq 2.1).

^b^ Orthologs in tetraploid wheat with GOC ≥50%. Genes with TRITD2 prefix are orthologous to *T. turgidum* ssp. *durum* (cv. Svevo), and those with prefix TRIDC2B are orthologous to *T. turgidum* ssp. *dicoccoides*.

Ancestral coefficient analysis based on this region dichotomized the accessions into two groups: group 1 and group 2, where accessions in each group shared more than 98% of ancestral proportion (Figure 4c,d). The former included all accessions of the hulled type Georgian wheat (*Triticum karamyschevii/Triticum turgium* subsp. *paleocolchicum*) and the free‐threshing Persian wheat (*Triticum carthlicum/T. turgidum* subsp. *carthlicum*), while the latter encompassed all the remaining tetraploids other than the Georgian and Persian wheats (Table S3). Phylogenetic analysis based on this region resulted in clear variations in branch length of the trees between the two groups. Although the topology of the trees provided little discrimination between the groups, accessions in group 1 had extremely long tree branches, while the branches for accessions in group 2 appeared nearly invisible (Figure 4e,f). The accessions with long branches (group 1) were mostly confined to European accessions and were at a high frequency in Georgia (Figure 4e,f and Table S3). The accessions in this group 1 were nearly identical to Georgian wheat (*T. karamyschevii/T. turgium* ssp. *paleocolchicum*) (Table S3).

## 4 DISCUSSION

Genetic signatures help to reconstruct historical selection and hybridization events that shaped the current crop genetic diversity. Understanding these historical events is vital for establishing conservation and breeding strategies to cope with the current climate change dynamics. Most crops were domesticated from few plants originating from a specific geographic region (Meyer & Purugganan, 2013). However, from this core germplasm, most crops spread to wide ecogeographic realms as a consequence of subsequent introgressions (gene flow) from their wild relatives through hybridization, selections for improved resilience over wide ranges and/or specialization for specific environments (Purugganan, 2019). The genetic signatures identified herein reflect these scenarios over the cultivation history of tetraploid wheats.

### 4.1 Population structure and haplotype distribution

The population structure in this study is based on the biological relationships of the GDP subset that includes wild and domesticated emmer wheats and durum wheat landraces. The detailed population genetic structure of the entire tetraploid wheat set including modern durum wheat cultivars was previously described (Maccaferri et al., 2019; Mazzucotelli et al., 2020). Unlike the previous studies that grouped the wild emmer accessions into two subpopulations (Maccaferri et al., 2019; Mazzucotelli et al., 2020), the GDP subset used herein clustered nearly all wild emmer accessions into a single group, independent of the methods employed to define the population structure: admixture, PCA, or phylogenetic NJ (Figure 1). On the other hand, the domesticated emmer wheat accessions, previously grouped into an Ethiopian (here we used the preferred term Abyssinian to describe the region) and a non‐Ethiopian or “other” group (Maccaferri et al., 2019), ended up being assigned to three subpopulations in our study, namely Abyssinian, West European–Mediterranean, and Middle East–Eastern European. The non‐Abyssinian domesticated emmer accessions clearly fell into two clades in both previous and current studies. This was also manifested by the ancestral coefficient analyses where the domesticated emmer wheat accessions fell into three or more subgroups as indicated by *K* = 5 (Maccaferri et al., 2019; Mazzucotelli et al., 2020).

Compared to other durum landrace subgroups and despite their relative geographic proximity, the higher differentiation of the Mediterranean landraces from the domesticated and wild emmer wheats can be attributed to post‐domestication historical selection pressure toward accessions adapted to the unique Mediterranean environment (Ceglar et al., 2021; Royo et al., 2014). This appears to be consistent with the patterns of adaptation of other crop species in the region (del Pozo et al., 2019; Sertse et al., 2019; Stella et al., 2013). Durum wheat is believed to have been introduced to agrarian land as secondary domestication (selection) from domesticated emmer wheat (Gioia et al., 2015) in Levantine (Great Syria: Jordan, Lebanon, Israel, and part of Turkey) which itself is part of the eastern Mediterranean (Kabbaj et al., 2017) and home of wild emmer wheat (Zohary et al., 2012). With the exception of the Abyssinian landraces, the relatively lower differentiation of the Mediterranean landraces compared to other landraces suggests a gene flow possibly radiating from the Mediterranean region as a source of germplasm (Moragues et al., 2007).

The Abyssinian landraces were interestingly more related to the wild emmer wheats than to any other subgroups. The Abyssinian durum was assumed to be peculiar in that it originated from Abyssinian domesticated emmer wheat as a tertiary domestication event of the crop (Vavilov, 1951). The present result, however, is consistent with Kabbaj et al. (2017), who argued that Ethiopia is a secondary center of diversification of the population that originated from the Levantine as opposed to Abyssinian emmer wheat being the result of an independent tertiary domestication. The Abyssinian highlands are known for harboring high genetic diversity in many crops, attributed in part to its topography which creates highly heterogeneous ecological zones and by many centuries of germplasm selection by farmers to best‐fit these diverse niches (Vavilov, 1951). As a result, earlier authors mentioned the region as a center of origin while it is more likely a center of secondary diversification (Harlan, 1969; Kabbaj et al., 2017; Milner et al., 2019; Sertse et al., 2019).

The durum wheat landraces appear to be closer to wild emmer wheat than to domesticated emmer wheat (Maccaferri et al., 2019). As displayed in the present study, and also reported by He et al. (2019), a high frequency of introgression from the wild emmer wheat gene pool was observed, thereby reducing the differentiation between contemporary wheat cultivars and wild emmer wheat accessions, while the differentiation between domesticated and wild emmer wheats remained unaffected. This suggests the continuous and greater gene flow between wild emmer wheats and durum landraces compared to that between the domesticated emmer wheats and the durum landraces, especially across areas where durum landraces and wild emmer wheats co‐exist (He et al., 2019; Luo et al., 2007; Syouf et al., 2006). Such massive gene flow from wild emmer to durum wheats, not only reduces the differentiation, but also increases the genetic diversity of the durum landraces (Syouf et al., 2006). The high genetic diversity in wild emmer wheat is indeed consistent with several previous reports (Avni et al., 2017; Maccaferri et al., 2019; Rahman et al., 2020; Scott et al., 2019), while the low genetic diversity in the Abyssinian domesticated emmer wheat subpopulation indicates the isolation of this population (Maccaferri et al., 2019). The haplotypes' geographic distribution based on the six adaptive loci depicts the gene flow across tetraploid wheat habitats. The dominant multi‐locus (Figure 3b, green) and single‐locus haplotypes (Figure S3a–f) that spread in all geographical regions represented in the GDP reflects the influence of contemporary germplasm transfer by breeders and explorers, mainly via durum wheat (Rahman et al., 2020). In the past century or so, numerous exchanges of superior germplasm (i.e., high yielding) took place among breeders of different continents, resulting in dramatic replacement of local landraces by more exotic elite materials (Reif et al., 2005). In extreme cases, a single cultivar dominates the growing regions of the crop (Kabbaj et al., 2017). The second most frequent multi‐locus haplotype (Figure 3b, red) can be associated with ecological adaptation to the north and east of the Mediterranean region. Although this haplotype is dominant in the North Mediterranean region, it is localized and did not spread, suggesting the distinct adaptation pattern of the germplasm for this region (Moragues et al., 2007; Soriano et al., 2018). The frequency (Table 3 and Figure S2) and geographic distribution (Figure S3a,b) of the single‐locus haplotypes “AAAT” at Chr1A:380188147–382858874, and “TATTA” at Chr1B:403564038–408577880 presumably reflect a recent gene flow from wild to cultivated tetraploid wheats in the Mediterranean region. The multi‐locus haplotype (Figure 3b, magenta) confined to the Levantine/Great Syria (Israel, Jordan, Lebanon, Syria, and part of Turkey) most likely exists only in the wild emmer wheat gene pool. The geographic distribution of the Abyssinian haplotypes: two multi‐locus (Figure 3b, dark green and dark blue) and the single‐locus haplotypes “AATT” (Figure S3a), “AATAT” (Figure S3b), and “TTAA” (Figure S3d,f) suggests a gene flow attributable to the ancient trades of the region (Boardman, 1999). These haplotypes are likely remnants of old migrant germplasm suited to the Abyssinian highlands (Samberg et al., 2010). They could also have originated from this region and subsequently spread to similar niches where they were adapted.

### 4.2 Adaptive loci and candidate genes

All six adaptive signature loci harbor genes that can be targeted by natural and anthropogenic selections (Table 3). For example, *TRITD1Av1G140940* and *TRITD1Av1G141030* on chromosome 1A are predicted to be *HISTONE ACETYLTRANSFERASE1* (*HAT1*) and *KUODA1* (meaning “enlarge” in Chinese) (*KUA1*) encoding genes, that are key in yield‐ and phenology‐related traits, respectively. The overexpression of *HAT1* increased yield in rice by contributing to grain size, grain number, spike length, grain filling, and overall biomass (Song et al., 2015). Histone deacetylase genes, such as *HATs*, are also involved in regulating phenology‐related biological processes, including seed dormancy (Hung et al., 2019; Zhou & Zhou et al., 2020), circadian clock (Hung et al., 2019), and flowering time through the regulation of flowering genes (Kim et al., 2016; Luo et al., 2015; Xiao et al., 2013). *HATs* are also involved in abiotic stress responses such as drought (Tan et al., 2018). The transcriptional factor *KUA1* is known for regulating leaf size in Arabidopsis, and being regulated with the circadian rhythm (Lu et al., 2014). Variations in genes that regulate yield traits and responses to stress conditions can be targeted by anthropogenic and natural selections. The signature locus overlaps with grain yield‐related QTL including QKns.mgb‐1A associated with kernel number per spike (Maccaferri et al., 2019; Mangini et al., 2018). The locus is also within an interval of two QTL (Maccaferri et al., 2019) for thousand kernel weight and grain weight per spike: *QTkw.fcu‐1A* and *QGws.fcu‐1A*, respectively, identified in a population developed by crossing durum with domesticated emmer wheat (Faris et al., 2014).

The *TRITD1Bv1G132830* and *TRITD1Bv1G132660* on chromosome 1B, encoding CALCINEURIN B‐LIKE PROTEIN 1 (CBL1) and CASEIN KINASE II BETA CHAIN 1 (CKB1), respectively, modulate responses to different stress conditions and regulate yield‐related traits. *CBL1* regulates responses to osmotic‐related abiotic stresses such as drought, salt, and cold conditions in plants (Chen et al., 2012; Cheong et al., 2003), including wheat (Cui et al., 2018). *CKBs* regulate circadian clock and flowering time (Lu et al., 2011) which can be important adaptive genes to day‐length conditions (Portolés & Más, 2007). They are also known to be involved in signal transduction in response to different stresses (Yuan et al., 2017). These genes are essential for the normal growth of plants and can also be involved in pathogenicity (Zhang et al., 2019). The physical position of the 1B locus is within a region (Maccaferri et al., 2019) that harbors important QTL such as for Fusarium head blight: *QFhs.mgb‐1BL* in a recombinant inbred line population developed by crossing a resistant hexaploid and a susceptible durum parents (Giancaspro et al., 2016) indicating the relevance of this locus for disease resistance. The 1B signature locus also overlaps with yield trait QTL for spike length in durum and domesticated emmer wheats (Giraldo et al., 2016).

The RECEPTOR FOR ACTIVATED C KINASE 1 (RACK1A) encoding gene, *TRITD3Av1G175540* at Chr3A:490120631–490513609 can modulate both phenological and yield traits. *RACK1A* is involved in circadian rhythm, seed germination, and dormancy in plants (Fennell et al., 2012; Zhang et al., 2014), including wheat (Bykova et al., 2011). This gene is also involved in plant immunity to diseases (Nakashima et al., 2008), stress response, and it is considered important for survival (Bykova et al., 2011; Chen et al., 2006). The other major gene on chromosome 3A is a *T. aestivum* ortholog predicted to encode ZINC FINGER PROTEIN 1 (ZFN1). *ZFN* genes prevent silique shattering in oilseed crops such as *Brassica* species (Tao et al., 2017). Shattering is a problem in many crops because it can contribute to major yield losses. These genes were also reported to increase seed oil in soybean (Li et al., 2017) and to regulate responses to several abiotic stresses (Sakamoto et al., 2000; Wang et al., 2019). In wheat, they regulate many phenological traits such as heading, flowering, and maturity times (Bapela et al., 2022; Qin et al., 2008) and play a role in stripe rust resistance (Guo et al., 2013). Zink finger protein genes in tandem with *DROUGHT AND SALT TOLERANCE (DST)* were found to enhance spike length, grain number, and consequently grain yield (Li et al., 2013). Generally, these genes have diverse roles that favor production, and their loci can be targeted in anthropogenic selections, including in modern improvement breeding programs. The physical position of locus Chr3A:490120631–490513609 is within the intervals that harbors QTL associated with yield and disease traits (Maccaferri et al., 2019).

The remaining genes of the outlier loci (Table 3) are also involved in regulating a number of adaptive and yield‐related traits. For example, the *CHLORIDE CHANNEL‐A* (*CLC‐A*) predicted genes are key in proline biosynthesis and enhanced drought and salt tolerances in Arabidopsis (Yang et al., 2018; Zhou et al., 2010). The two genes on chromosome 7A: *TRITD7Av1G254470* and *TRITD7Av1G254580*, with orthologs of *TRAESCS7A02G484800* and *TRAESCS7A02G485300*, respectively, are also likely involved in regulating traits that are targeted by breeding. *TRITD7Av1G254470*, annotated as *RESISTANCE RELATED KINASE 1* (*RKS1*), confers broad‐spectrum disease resistance, including resistance to vascular diseases caused by *Xanthomonas campestris* (Delplace et al., 2020; Huard‐Chauveau et al., 2013). The 7A signature locus is also within the physical interval of a QTL associated with FHB in population derived from FHB‐resistant Tunisian lines (Ghavami et al., 2011; Maccaferri et al., 2019). *TRITD7Av1G254580* at this locus is predicted to encode FK506‐BINDING PROTEIN 13 (FKBP13), which regulates plant photosynthesis including in wheat (Gollan et al., 2011) and controls developmental processes such as branching in cabbages (Gollan et al., 2011; Guo et al., 2021; Ingelsson et al., 2009).

The uniquely differentiated region on chromosome 2B is a signature of an alien introgression in tetraploid wheat species that is consistent with the alien introgression from *Triticum timopheevii* recently reported in hexaploid wheat by Walkowiak et al. (2020). While the similarity of the majority of the tetraploid accessions in the region was expected because it has likely descended from the wild emmer progenitor (Avni et al., 2017), the deviance of the group 1′s accessions and their geographic distribution is consistent with the natural habitat of *T. timopheevii* and suggests the historical gene flow between this species and other wheat types that have been cultivated in Georgia and its vicinity for millennia (Bedoshvili et al., 2020). *T. timopheevii* is known for its adaptation to extreme abiotic stresses, such as saline conditions (Badridze et al., 2009) and diseases, including Fusarium head blight (Malihipour et al., 2016) and stripe rust (Bariana et al., 2001; Kosgey et al., 2020). As a result, the species has been a source of resistance to both abiotic and biotic stresses as well as other traits targeted for improvement in breeding programs via introgression (Devi et al., 2019; Steed et al., 2022).

Considering the genes it harbors, the 2B signature region in the current study is vital for adaptation to environmental conditions and improvement of target traits such as yield. For example, the predicted *PHYTOCHROME INTERACTING FACTOR 4* (*PIF4*), a gene that has a reportedly fundamental role in plant thermomorphogenesis, is located in the introgressed segment (Casal & Balasubramanian, 2019; Perrella et al., 2022). This gene regulates numerous physiological and developmental processes by controlling several other genes (Xu, 2018). PIFs are key components in coordinating several internal processes such as hormonal pathways, circadian clock, and responses to external stresses such as light, temperature, and competitors (Leivar & Monte, 2014). PIFs are known to regulate the growth of plants in Arabidopsis via interacting with the major photomorphogenesis repressor CONSTITUTIVELY PHOTOMORPHOGENIC 1 (COP1) protein (Kathare et al., 2020; Xu et al., 2015). It also confers resistance to diseases and is suggested to be a key target in breeding for temperature resilience and disease resistance (Gangappa et al., 2017). Consistent with the presence of *PIF4* and other potential diseaseresistance genes believed to have been derived from *T. timopheevii* (Bansal et al., 2014; Walkowiak et al., 2020), the region is known to confer resistance to several rust diseases in wheat (Bansal et al., 2014; Chemayek et al., 2017). The *SQUAMOSA PROMOTER BINDING PROTEIN‐LIKE3* (*SPL3*) gene in this region also regulates flowering time in response to temperature conditions (Lee et al., 2012) and vernalization responses (Schiessl et al., 2014; Zhou et al., 2013). The *CALMODULIN‐BINDING TRANSCRIPTION FACTORS* (CBTs) were postulated to have a negative role in disease resistance regulation in wheat (Wang et al., 2019) and other crops such as rice (Koo et al., 2009). These are a few examples but other genes in the region are also potentially involved in regulating a number of physiological and developmental traits that can be important for plants’ adaptation to environmental conditions.

## 5 CONCLUSION

Principal‐component‐based tetraploid wheat genome scans captured adaptive loci that harbor genes potentially playing essential roles in regulating traits of environmental adaptation as well as yield and its components, suggesting that these loci have been under natural and anthropogenic selections in the tetraploid wheat genome for a long time. The single dominant haplotype that spread across the entire tetraploid wheat‐growing regions, while most others (*n* = 7) are confined to certain geographic regions, reflect the effect of contemporary breeding and swift transfer of yield and yield‐related traits in the development of elite germplasm. This not only decreases the diversity, creating genetic bottlenecks in contemporary breeding populations, but also leads to limited elite materials, eventually overtaking local landraces in multiple geographic regions, that can result in the extinction of critical adaptive genes. Strategies can be devised based on the haplotype knowledge described herein to ensure conservation of all haplotypes in gene banks and their use in breeding programs. This is particularly critical for the haplotypes confined to particular geographic regions because they likely contain genetics specific to their adaptation to these environments. The outlier loci in this study can be used as genetic signatures in future studies to assist in maintaining the representation of germplasm in breeding and conservation populations. In this study, the putative functions of genes in LD with adaptive loci were inferred from their orthologs in *T. aestivum* and *A. thaliana*. We recommend further validation of these genes through functional genomics and reverse genetic analyses, particularly those with strong adaptive affiliations and those that are prime targets of selection. The genome scan approach we applied in this study has yielded relevant results that hint at many avenues for further studies and possible applications. Given the current availability of public genetic data for multiple species and the current low cost of genotyping, we recommend this approach for profiling germplasm housed in gene banks and in situ in the search for candidate germplasm that are either environmentally resilient and/or niche adapted for breeding and those that require conservation attention. Considering the freely available multi‐years environmental grid data, the genome scan outcomes could further be supported by genome–environment association (GEA) studies. Where geographic coordinates of germplasm collection sites are included in passport data, it is possible to obtaindata for key climate and edaphic factors of a site from open‐source databases such as NASA.

## AUTHOR CONTRIBUTIONS

**Demissew Sertse**: Conceptualization; data curation; formal analysis; investigation; methodology; resources; software; visualization; writing—original draft. **Jemanesh K. Haile**: Conceptualization; investigation; methodology; writing—review and editing. **Ehsan Sari**: Investigation; methodology; writing—review and editing. **Valentyna Klymiuk**: Investigation; methodology; writing—review and editing. **Amidou N'Diaye**: Investigation; methodology; writing—review and editing. **Curtis J. Pozniak**: Methodology; validation; writing—review and editing. **Sylvie Cloutier**: Investigation; methodology; validation; writing—review and editing. **Sateesh Kagale**: Funding acquisition; investigation; supervision; writing—review and editing.

## CONFLICT OF INTEREST STATEMENT

The authors declare that they have no known competing financial interests or personal relationships that could have appeared to influence the work reported in this paper.

## Supporting information



SUPPLEMENTAL FILES AND CAPTIONS
Table S1. Durum Global Diversity Panel (GDP) passport information and ancestral coefficients. Accessions 1the first three letters indicate the name of the country based on united nations three letter country code, followed by accession names at GDP. Each color indicates the population based on the ancestral coefficient of the individuals at K = 9
**Figure S1**. Pattern of differentiation at each locus based on *F_ST_* and *G''_ST_*.
**Figure S2**. Allelic frequencies the SNP at each locus in Landrace, domesticated and wild emmer wheats. The *y* axis shows the percent frequency and the labels show the absolute frequencies.
**Figure S3**. The geographic distribution of single‐locus haplotypes. (**a**) Haplotypes at locus 1A, **(b**) haplotype 1B (**c**) haplotype 3A1, (**d**) haplotype 3A2, (**e**) haplotype 6A and (f) haplotype 7A. Each color in each pie chart represents a haplotype, the size of the pie circle is proportional with the number of accessions included.
**Table S2**. Major Genes in the uniquely differentiated region on chromosome 2B. Cr = Circadian clocking, FL = Flowering, Sdr = Seed dormancy, Vr = Vernalization.
**Table S3**. Passport information of the accessions and ancestral coefficients (Q1 and Q2) based on the unique 2B region from genome scan of WG resequencing data. Passport information was extracted from (Zhou & Zhou et al., 2020), each color shade indicates accessions sharing over 98% of their ancestral proportion.

## Data Availability

The study used two sets of genotype data: 90K SNP array of 442 accessions from Durum Global Diversity Panel (GDP) and SNPs obtained by whole‐genome resequencing of 121 tetraploid wheat accessions downloaded from https://wheat.pw.usda.gov/GG3/global_durum_genomic_resources and http://bigd.big.ac.cn/gvm, respectively. All other data presented in this study are included in the manuscript.
