# Supplementary material for: Genome scans capture key adaptation and historical hybridization signatures in tetraploid wheat
Source: Plant Genome. 2023 Nov 16;18(1):e20410. doi: 10.1002/tpg2.20410 (PMC11726425; doi:10.1002/tpg2.20410)
Supplement: Supplementary file 1 — SUPPLEMENTAL FILES AND CAPTIONS Table S1. Durum Global Diversity Panel (GDP) passport information and ancestral coefficients. Accessions 1the first three letters indicate the name of the country based on united nations three letter country code, followed by accession names at GDP. Each color indicates the population based on the ancestral coefficient of the individuals at K = 9 Figure S1. Pattern of differentiation at each locus based on FST and G''ST . Figure S2. Allelic frequencies the SNP at each locus in Landrace, domesticated and wild emmer wheats. The y axis shows the percent frequency and the labels show the absolute frequencies. Figure S3. The geographic distribution of single‐locus haplotypes. (a) Haplotypes at locus 1A, (b) haplotype 1B (c) haplotype 3A1, (d) haplotype 3A2, (e) haplotype 6A and (f) haplotype 7A. Each color in each pie chart represents a haplotype, the size of the pie circle is proportional with the number of accessions included. Table S2. Major Genes in the uniquely differentiated region on chromosome 2B. Cr = Circadian clocking, FL = Flowering, Sdr = Seed dormancy, Vr = Vernalization. Table S3. Passport information of the accessions and ancestral coefficients (Q1 and Q2) based on the unique 2B region from genome scan of WG resequencing data. Passport information was extracted from (Zhou & Zhou et al., 2020), each color shade indicates accessions sharing over 98% of their ancestral proportion. [file TPG2-18-e20410-s001.docx]

**Supplementary Materials**

Analyses in this manuscript were based on two genotype datasets: the 90K SNPs data of Global Durum Wheat Panel (GDP) (n=1011) non cultivar subset (n=442, assumption: landraces, domesticated and wild emmer show better historical adaptive signatures) from Mazzucotelli et al 2020 and the other was only tetraploid WG genotype subset data extracted from Zhou et al 2020. Most of the stories are based on the former; but the genome scan based on the second dataset captured a uniquely differentiated region on 2B, which matches the findings in Walkowiak et al 2020. The entire population in the second dataset was strikingly dichotomized in this region into Georgian wheat (*Triticum karamyschevii*) plus some *T. turgidum* and the rest of *T. turgidum*, including wild emmer (*Triticum turgidum ssp. dicoccoides),* and *T. ispahanicum*

**Table S1.** Durum Global Diversity Panel (GDP) passport information and ancestral coefficients.

| **Accession^1^** | **GDPaccess** | **Subspecies** | **Type** | **Q1** | **Q2** | **Q3** | **Q4** | **Q5** | **Q6** | **Q7** | **Q8** | **Q9** | **POP** |
| --- | --- | --- | --- | --- | --- | --- | --- | --- | --- | --- | --- | --- | --- |
| YEM_GDPv2-608 | DWRC-1197 | T.durum | Landrace | 0.413932 | 0.065705 | 0.001039 | 0.025731 | 0.411471 | 0.012249 | 1.00E-04 | 0.044041 | 0.025733 | ABYS_DOM |
| ETH_GDPv2-875 | DWRC-1524 | T.dicoccum | D_Emmer | 0.999201 | 9.99E-05 | 9.99E-05 | 9.99E-05 | 9.99E-05 | 9.99E-05 | 9.99E-05 | 9.99E-05 | 9.99E-05 | ABYS_DOM |
| UK_GDPv2-888 | DWRC-1549 | T.dicoccum | D_Emmer | 0.624341 | 0.105912 | 0.043734 | 0.006296 | 1.00E-04 | 0.04063 | 0.051827 | 0.016488 | 0.110672 | ABYS_DOM |
| ETH_GDPv2-893 | DWRC-1569 | T.dicoccum | D_Emmer | 0.999201 | 9.99E-05 | 9.99E-05 | 9.99E-05 | 9.99E-05 | 9.99E-05 | 9.99E-05 | 9.99E-05 | 9.99E-05 | ABYS_DOM |
| AFG_GDPv2-901 | DWRC-1583 | T.dicoccum | D_Emmer | 0.999201 | 9.99E-05 | 9.99E-05 | 9.99E-05 | 9.99E-05 | 9.99E-05 | 9.99E-05 | 9.99E-05 | 9.99E-05 | ABYS_DOM |
| ETH_GDPv2-912 | DWRC-1610 | T.dicoccum | D_Emmer | 0.689683 | 9.99E-05 | 0.303588 | 9.99E-05 | 9.99E-05 | 9.99E-05 | 9.99E-05 | 9.99E-05 | 0.00613 | ABYS_DOM |
| ETH_GDPv2-913 | DWRC-1611 | T.dicoccum | D_Emmer | 0.999201 | 9.99E-05 | 9.99E-05 | 9.99E-05 | 9.99E-05 | 9.99E-05 | 9.99E-05 | 9.99E-05 | 9.99E-05 | ABYS_DOM |
| ETH_GDPv2-914 | DWRC-1613 | T.dicoccum | D_Emmer | 0.999201 | 9.99E-05 | 9.99E-05 | 9.99E-05 | 9.99E-05 | 9.99E-05 | 9.99E-05 | 9.99E-05 | 9.99E-05 | ABYS_DOM |
| ETH_GDPv2-915 | DWRC-1615 | T.dicoccum | D_Emmer | 0.602753 | 0.020049 | 1.00E-04 | 0.06164 | 0.223616 | 0.033729 | 1.00E-04 | 0.057914 | 1.00E-04 | ABYS_DOM |
| ETH_GDPv2-916 | DWRC-1618 | T.dicoccum | D_Emmer | 0.999201 | 9.99E-05 | 9.99E-05 | 9.99E-05 | 9.99E-05 | 9.99E-05 | 9.99E-05 | 9.99E-05 | 9.99E-05 | ABYS_DOM |
| ETH_GDPv2-917 | DWRC-1619 | T.dicoccum | D_Emmer | 0.438471 | 1.00E-04 | 1.00E-04 | 0.018666 | 0.121318 | 0.240333 | 0.171307 | 0.009605 | 1.00E-04 | ABYS_DOM |
| ETH_GDPv2-918 | DWRC-1621 | T.dicoccum | D_Emmer | 0.588716 | 0.119536 | 0.007267 | 1.00E-04 | 0.10414 | 0.150653 | 0.008877 | 0.020611 | 1.00E-04 | ABYS_DOM |
| IND_GDPv2-921 | DWRC-1633 | T.dicoccum | D_Emmer | 0.476394 | 0.371559 | 1.00E-04 | 0.037752 | 1.00E-04 | 0.043908 | 0.038833 | 1.00E-04 | 0.031255 | ABYS_DOM |
| ETH_GDPv2-940 | DWRC-1690 | T.dicoccum | D_Emmer | 0.999201 | 9.99E-05 | 9.99E-05 | 9.99E-05 | 9.99E-05 | 9.99E-05 | 9.99E-05 | 9.99E-05 | 9.99E-05 | ABYS_DOM |
| ETH_GDPv2-943 | DWRC-1697 | T.dicoccum | D_Emmer | 0.999201 | 9.99E-05 | 9.99E-05 | 9.99E-05 | 9.99E-05 | 9.99E-05 | 9.99E-05 | 9.99E-05 | 9.99E-05 | ABYS_DOM |
| IND_GDPv2-944 | DWRC-1698 | T.dicoccum | D_Emmer | 0.90825 | 0.021179 | 0.006727 | 0.002785 | 1.00E-04 | 1.00E-04 | 1.00E-04 | 1.00E-04 | 0.060659 | ABYS_DOM |
| ETH_GDPv2-947 | DWRC-1705 | T.dicoccum | D_Emmer | 0.999201 | 9.99E-05 | 9.99E-05 | 9.99E-05 | 9.99E-05 | 9.99E-05 | 9.99E-05 | 9.99E-05 | 9.99E-05 | ABYS_DOM |
| SYR_GDPv2-950 | DWRC-1709 | T.dicoccum | D_Emmer | 0.901678 | 0.023763 | 0.008964 | 0.001872 | 1.00E-04 | 1.00E-04 | 1.00E-04 | 1.00E-04 | 0.063324 | ABYS_DOM |
| CND_GDPv2-951 | DWRC-1710 | T.dicoccum | D_Emmer | 0.909985 | 0.023469 | 0.001478 | 1.00E-04 | 1.00E-04 | 1.00E-04 | 1.00E-04 | 1.00E-04 | 0.064569 | ABYS_DOM |
| ETH_GDPv2-388 | DWRC-2166 | T.durum | Landrace | 0.055518 | 9.99E-05 | 9.99E-05 | 0.943782 | 9.99E-05 | 9.99E-05 | 9.99E-05 | 9.99E-05 | 9.99E-05 | ABYS_LND |
| ERI_GDPv2-389 | DWRC-2167 | T.durum | Landrace | 0.076295 | 1.00E-04 | 1.00E-04 | 0.769384 | 0.122798 | 0.021218 | 0.009905 | 1.00E-04 | 1.00E-04 | ABYS_LND |
| GRC_GDPv2-395 | DWRC-2218 | T.durum | Landrace | 1.00E-04 | 0.025727 | 0.15996 | 0.239181 | 0.111704 | 0.111037 | 1.00E-04 | 0.150155 | 0.202036 | ABYS_LND |
| ARG_GDPv2-398 | DWRC-2226 | T.durum | Landrace | 0.027901 | 9.99E-05 | 9.99E-05 | 0.971399 | 9.99E-05 | 9.99E-05 | 9.99E-05 | 9.99E-05 | 9.99E-05 | ABYS_LND |
| ETH_GDPv2-402 | DWRC-2236 | T.durum | Landrace | 0.029148 | 9.99E-05 | 9.99E-05 | 0.96905 | 9.99E-05 | 0.001202 | 9.99E-05 | 9.99E-05 | 9.99E-05 | ABYS_LND |
| ESP_GDPv2-582 | DWRC-1154 | T.durum | Landrace | 0.062112 | 9.99E-05 | 9.99E-05 | 0.937189 | 9.99E-05 | 9.99E-05 | 9.99E-05 | 9.99E-05 | 9.99E-05 | ABYS_LND |
| GRC_GDPv2-586 | DWRC-1159 | T.durum | Landrace | 1.00E-04 | 1.00E-04 | 0.162816 | 0.4252 | 0.013279 | 1.00E-04 | 1.00E-04 | 0.100859 | 0.297445 | ABYS_LND |
| ETH_GDPv2-592 | DWRC-1166 | T.durum | Landrace | 1.00E-04 | 1.00E-04 | 1.00E-04 | 0.423602 | 0.007533 | 0.220582 | 0.265124 | 0.08276 | 1.00E-04 | ABYS_LND |
| ETH_GDPv2-594 | DWRC-1174 | T.durum | Landrace | 0.070314 | 1.00E-04 | 1.00E-04 | 0.785506 | 0.084336 | 0.059345 | 1.00E-04 | 1.00E-04 | 1.00E-04 | ABYS_LND |
| ETH_GDPv2-595 | DWRC-1175 | T.durum | Landrace | 0.067696 | 1.00E-04 | 1.00E-04 | 0.860841 | 0.031433 | 0.03953 | 1.00E-04 | 1.00E-04 | 1.00E-04 | ABYS_LND |
| ETH_GDPv2-596 | DWRC-1176 | T.durum | Landrace | 0.095176 | 1.00E-04 | 1.00E-04 | 0.796456 | 0.064305 | 0.043564 | 1.00E-04 | 1.00E-04 | 1.00E-04 | ABYS_LND |
| ETH_GDPv2-601 | DWRC-1184 | T.durum | Landrace | 1.00E-04 | 0.009004 | 0.005464 | 0.304155 | 0.152877 | 0.218634 | 0.207208 | 0.102458 | 1.00E-04 | ABYS_LND |
| YEM_GDPv2-607 | DWRC-1196 | T.durum | Landrace | 0.00708 | 0.008316 | 1.00E-04 | 0.525275 | 0.388978 | 0.047051 | 0.023 | 1.00E-04 | 1.00E-04 | ABYS_LND |
| ETH_GDPv2-617 | DWRC-1209 | T.durum | Landrace | 0.055614 | 1.00E-04 | 1.00E-04 | 0.85612 | 0.07909 | 0.000346 | 0.008432 | 1.00E-04 | 1.00E-04 | ABYS_LND |
| SAU_GDPv2-619 | DWRC-1212 | T.durum | Landrace | 0.043009 | 1.00E-04 | 1.00E-04 | 0.323793 | 0.317416 | 0.119404 | 0.112128 | 0.029323 | 0.054728 | ABYS_LND |
| ETH_GDPv2-622 | DWRC-1217 | T.durum | Landrace | 0.071422 | 1.00E-04 | 1.00E-04 | 0.858381 | 0.057002 | 0.001485 | 0.01131 | 1.00E-04 | 1.00E-04 | ABYS_LND |
| ETH_GDPv2-623 | DWRC-1218 | T.durum | Landrace | 0.053739 | 1.00E-04 | 1.00E-04 | 0.797294 | 0.113758 | 0.03471 | 1.00E-04 | 1.00E-04 | 1.00E-04 | ABYS_LND |
| ETH_GDPv2-625 | DWRC-1222 | T.durum | Landrace | 0.075139 | 9.99E-05 | 9.99E-05 | 0.924162 | 9.99E-05 | 9.99E-05 | 9.99E-05 | 9.99E-05 | 9.99E-05 | ABYS_LND |
| TUN_GDPv2-672 | DWRC-1304 | T.durum | Landrace | 0.013676 | 1.00E-04 | 1.00E-04 | 0.573246 | 0.207585 | 0.03532 | 0.169772 | 1.00E-04 | 1.00E-04 | ABYS_LND |
| ETH_GDPv2-673 | DWRC-1305 | T.durum | Landrace | 0.021356 | 1.00E-04 | 1.00E-04 | 0.752727 | 0.135951 | 0.089466 | 1.00E-04 | 1.00E-04 | 1.00E-04 | ABYS_LND |
| ETH_GDPv2-682 | DWRC-1316 | T.durum | Landrace | 0.03874 | 9.99E-05 | 9.99E-05 | 0.960561 | 9.99E-05 | 9.99E-05 | 9.99E-05 | 9.99E-05 | 9.99E-05 | ABYS_LND |
| TUN_GDPv2-694 | DWRC-1332 | T.durum | Landrace | 0.014521 | 0.006086 | 0.026688 | 0.427468 | 0.087921 | 0.111113 | 0.232397 | 0.093706 | 1.00E-04 | ABYS_LND |
| ETH_GDPv2-745 | DWRC-2025 | T.durum | Landrace | 0.010171 | 0.004747 | 1.00E-04 | 0.961319 | 1.00E-04 | 0.015608 | 1.00E-04 | 0.007755 | 1.00E-04 | ABYS_LND |
| EGY_GDPv2-785 | DWRC-1954 | T.polonicum | Landrace | 0.070487 | 1.00E-04 | 1.00E-04 | 0.87964 | 0.034786 | 0.009311 | 0.005376 | 1.00E-04 | 1.00E-04 | ABYS_LND |
| ETH_GDPv2-786 | DWRC-1955 | T.polonicum | Landrace | 0.071665 | 0.009 | 1.00E-04 | 0.778156 | 0.103672 | 0.024352 | 1.00E-04 | 0.012855 | 1.00E-04 | ABYS_LND |
| TUR_GDPv2-791 | DWRC-1962 | T.turgidum | Landrace | 1.00E-04 | 1.00E-04 | 0.156004 | 0.336158 | 0.07524 | 1.00E-04 | 1.00E-04 | 0.192224 | 0.239975 | ABYS_LND |
| PRT_GDPv2-795 | DWRC-1968 | T.turgidum | Landrace | 1.00E-04 | 1.00E-04 | 0.123293 | 0.762364 | 1.00E-04 | 1.00E-04 | 1.00E-04 | 0.036204 | 0.07764 | ABYS_LND |
| GEO_GDPv2-802 | DWRC-1979 | T.carthlicum | Landrace | 1.00E-04 | 1.00E-04 | 0.157627 | 0.410394 | 1.00E-04 | 1.00E-04 | 1.00E-04 | 0.140522 | 0.290958 | ABYS_LND |
| RUS_GDPv2-804 | DWRC-1981 | T.carthlicum | Landrace | 1.00E-04 | 1.00E-04 | 0.135249 | 0.757265 | 1.00E-04 | 1.00E-04 | 1.00E-04 | 0.027845 | 0.079141 | ABYS_LND |
| GEO_GDPv2-805 | DWRC-1982 | T.carthlicum | Landrace | 1.00E-04 | 1.00E-04 | 0.1215 | 0.759539 | 1.00E-04 | 1.00E-04 | 1.00E-04 | 0.034861 | 0.0836 | ABYS_LND |
| RUS_GDPv2-806 | DWRC-1983 | T.carthlicum | Landrace | 1.00E-04 | 1.00E-04 | 0.141673 | 0.7639 | 1.00E-04 | 1.00E-04 | 1.00E-04 | 0.016645 | 0.077282 | ABYS_LND |
| TUR_GDPv2-807 | DWRC-1984 | T.carthlicum | Landrace | 1.00E-04 | 1.00E-04 | 0.122884 | 0.723254 | 1.00E-04 | 1.00E-04 | 1.00E-04 | 0.070229 | 0.083134 | ABYS_LND |
| GEO_GDPv2-808 | DWRC-1985 | T.carthlicum | Landrace | 1.00E-04 | 1.00E-04 | 0.120043 | 0.759573 | 1.00E-04 | 1.00E-04 | 1.00E-04 | 0.026747 | 0.093137 | ABYS_LND |
| GEO_GDPv2-809 | DWRC-1986 | T.carthlicum | Landrace | 1.00E-04 | 1.00E-04 | 0.129223 | 0.756307 | 1.00E-04 | 1.00E-04 | 1.00E-04 | 0.035301 | 0.078669 | ABYS_LND |
| ETH_GDPv2-810 | DWRC-2018 | T.turgidum | Landrace | 0.061767 | 9.99E-05 | 9.99E-05 | 0.885453 | 0.05218 | 9.99E-05 | 9.99E-05 | 9.99E-05 | 9.99E-05 | ABYS_LND |
| ETH_GDPv2-811 | DWRC-2019 | T.durum | Landrace | 0.048655 | 9.99E-05 | 9.99E-05 | 0.907918 | 0.042827 | 9.99E-05 | 9.99E-05 | 9.99E-05 | 9.99E-05 | ABYS_LND |
| na_GDPv2-812 | DWRC-2020 | T.aethiopicum | Landrace | 0.024283 | 9.99E-05 | 9.99E-05 | 0.975017 | 9.99E-05 | 9.99E-05 | 9.99E-05 | 9.99E-05 | 9.99E-05 | ABYS_LND |
| ETH_GDPv2-817 | DWRC-2027 | T.durum | Landrace | 0.056079 | 9.99E-05 | 9.99E-05 | 0.90953 | 0.033791 | 9.99E-05 | 9.99E-05 | 9.99E-05 | 9.99E-05 | ABYS_LND |
| GEO_GDPv2-818 | DWRC-2138 | T.carthlicum | Landrace | 1.00E-04 | 1.00E-04 | 0.112264 | 0.754314 | 1.00E-04 | 1.00E-04 | 1.00E-04 | 0.074599 | 0.058324 | ABYS_LND |
| GEO_GDPv2-819 | DWRC-2139 | T.carthlicum | Landrace | 1.00E-04 | 1.00E-04 | 0.122179 | 0.764429 | 1.00E-04 | 1.00E-04 | 1.00E-04 | 0.046336 | 0.066556 | ABYS_LND |
| ESP_GDPv2-936 | DWRC-1678 | T.dicoccum | D_Emmer | 0.035127 | 1.00E-04 | 0.011338 | 0.416372 | 1.00E-04 | 0.281576 | 0.255188 | 1.00E-04 | 1.00E-04 | ABYS_LND |
| na_GDPv2-957 | DWRC-1987 | T.carthlicum | Landrace | 1.00E-04 | 1.00E-04 | 0.12765 | 0.70895 | 1.00E-04 | 1.00E-04 | 1.00E-04 | 0.074811 | 0.08809 | ABYS_LND |
| na_GDPv2-958 | DWRC-1989 | T.carthlicum | Landrace | 1.00E-04 | 0.021041 | 1.00E-04 | 0.264405 | 0.079872 | 0.26182 | 0.18921 | 0.183352 | 1.00E-04 | ABYS_LND |
| ARG_GDPv2-043 | DWRC-0169 | T.durum | Landrace | 0.033456 | 1.00E-04 | 0.016079 | 0.058616 | 0.071395 | 0.495534 | 0.32258 | 1.00E-04 | 0.002139 | EEU_LND |
| HUN_GDPv2-1007 | DWRC-2232 | T.durum | Landrace | 0.001392 | 0.143994 | 0.113546 | 1.00E-04 | 0.077345 | 0.270818 | 0.231849 | 0.14705 | 0.013906 | EEU_LND |
| ICARDA_GDPv2-193 | DWRC-0685 | T.durum | Landrace | 0.027476 | 0.029487 | 1.00E-04 | 0.027209 | 0.307127 | 0.522193 | 0.051914 | 1.00E-04 | 0.034395 | EEU_LND |
| CND_GDPv2-221 | DWRC-0729 | T.durum | Landrace | 0.085954 | 1.00E-04 | 0.054106 | 1.00E-04 | 0.022528 | 0.830933 | 1.00E-04 | 1.00E-04 | 0.006079 | EEU_LND |
| USA_GDPv2-366 | DWRC-2087 | T.durum | Landrace | 9.99E-05 | 9.99E-05 | 0.030558 | 9.99E-05 | 9.99E-05 | 0.968742 | 9.99E-05 | 9.99E-05 | 9.99E-05 | EEU_LND |
| USA_GDPv2-387 | DWRC-2160 | T.durum | Landrace | 1.00E-04 | 1.00E-04 | 1.00E-04 | 1.00E-04 | 0.233918 | 0.67864 | 0.077783 | 0.00916 | 1.00E-04 | EEU_LND |
| AUS_GDPv2-392 | DWRC-2186 | T.durum | Landrace | 1.00E-04 | 1.00E-04 | 1.00E-04 | 1.00E-04 | 0.085275 | 0.503251 | 0.211646 | 0.164523 | 0.034906 | EEU_LND |
| KAZ_GDPv2-394 | DWRC-2215 | T.durum | Landrace | 9.99E-05 | 9.99E-05 | 9.99E-05 | 9.99E-05 | 9.99E-05 | 0.999201 | 9.99E-05 | 9.99E-05 | 9.99E-05 | EEU_LND |
| POL_GDPv2-400 | DWRC-2228 | T.durum | Landrace | 1.00E-04 | 1.00E-04 | 1.00E-04 | 0.025775 | 0.027037 | 0.92018 | 0.026508 | 1.00E-04 | 1.00E-04 | EEU_LND |
| JOR_GDPv2-557 | DWRC-1008 | T.durum | Landrace | 0.045271 | 1.00E-04 | 0.067778 | 1.00E-04 | 0.02655 | 0.789584 | 0.070418 | 1.00E-04 | 1.00E-04 | EEU_LND |
| TUR_GDPv2-558 | DWRC-1009 | T.durum | Landrace | 1.00E-04 | 1.00E-04 | 1.00E-04 | 1.00E-04 | 0.306485 | 0.53059 | 0.096904 | 0.065521 | 1.00E-04 | EEU_LND |
| TUR_GDPv2-561 | DWRC-1012 | T.durum | Landrace | 1.00E-04 | 0.022578 | 1.00E-04 | 1.00E-04 | 0.063596 | 0.581541 | 0.173599 | 0.158285 | 1.00E-04 | EEU_LND |
| ESP_GDPv2-565 | DWRC-1029 | T.durum | Landrace | 1.00E-04 | 0.106645 | 0.074391 | 0.007694 | 1.00E-04 | 0.551085 | 0.242943 | 1.00E-04 | 0.016941 | EEU_LND |
| KAZ_GDPv2-573 | DWRC-1121 | T.durum | Landrace | 1.00E-04 | 0.016484 | 1.00E-04 | 0.03747 | 0.028916 | 0.910636 | 0.006095 | 1.00E-04 | 1.00E-04 | EEU_LND |
| KAZ_GDPv2-574 | DWRC-1122 | T.durum | Landrace | 0.012237 | 0.012121 | 0.053692 | 0.016875 | 0.019643 | 0.875702 | 1.00E-04 | 1.00E-04 | 0.00953 | EEU_LND |
| DZA_GDPv2-588 | DWRC-1161 | T.durum | Landrace | 0.07576 | 1.00E-04 | 0.050794 | 1.00E-04 | 0.020955 | 0.842345 | 1.00E-04 | 1.00E-04 | 0.009747 | EEU_LND |
| TUR_GDPv2-597 | DWRC-1177 | T.durum | Landrace | 1.00E-04 | 0.001605 | 1.00E-04 | 0.016643 | 0.403227 | 0.503463 | 0.047906 | 0.026857 | 1.00E-04 | EEU_LND |
| AFG_GDPv2-599 | DWRC-1182 | T.durum | Landrace | 9.99E-05 | 9.99E-05 | 9.99E-05 | 9.99E-05 | 0.038698 | 0.710682 | 0.25002 | 9.99E-05 | 9.99E-05 | EEU_LND |
| AFG_GDPv2-600 | DWRC-1183 | T.durum | Landrace | 1.00E-04 | 1.00E-04 | 1.00E-04 | 1.00E-04 | 0.252143 | 0.593447 | 0.070359 | 0.083551 | 1.00E-04 | EEU_LND |
| KAZ_GDPv2-605 | DWRC-1193 | T.durum | Landrace | 1.00E-04 | 0.053046 | 0.029367 | 0.02531 | 0.005069 | 0.794796 | 0.037811 | 0.016754 | 0.037747 | EEU_LND |
| ARM_GDPv2-613 | DWRC-1203 | T.durum | Landrace | 1.00E-04 | 1.00E-04 | 0.006841 | 1.00E-04 | 0.061003 | 0.694156 | 0.221808 | 0.015792 | 1.00E-04 | EEU_LND |
| ARM_GDPv2-615 | DWRC-1206 | T.durum | Landrace | 1.00E-04 | 1.00E-04 | 1.00E-04 | 1.00E-04 | 0.344225 | 0.593601 | 0.025073 | 0.036601 | 1.00E-04 | EEU_LND |
| DZA_GDPv2-640 | DWRC-1243 | T.durum | Landrace | 9.99E-05 | 9.99E-05 | 9.99E-05 | 9.99E-05 | 9.99E-05 | 0.980991 | 9.99E-05 | 0.018309 | 9.99E-05 | EEU_LND |
| PRT_GDPv2-643 | DWRC-1246 | T.durum | Landrace | 0.031325 | 1.00E-04 | 1.00E-04 | 0.025434 | 0.28853 | 0.303542 | 0.264848 | 0.086021 | 1.00E-04 | EEU_LND |
| TUN_GDPv2-645 | DWRC-1248 | T.durum | Landrace | 1.00E-04 | 0.012476 | 1.00E-04 | 1.00E-04 | 0.043783 | 0.457809 | 0.333863 | 0.151669 | 1.00E-04 | EEU_LND |
| TUN_GDPv2-648 | DWRC-1257 | T.durum | Landrace | 1.00E-04 | 0.002685 | 1.00E-04 | 1.00E-04 | 1.00E-04 | 0.660188 | 0.324473 | 0.012154 | 1.00E-04 | EEU_LND |
| TUN_GDPv2-650 | DWRC-1259 | T.durum | Landrace | 1.00E-04 | 1.00E-04 | 1.00E-04 | 1.00E-04 | 0.072103 | 0.804702 | 0.093927 | 0.028769 | 1.00E-04 | EEU_LND |
| UKR_GDPv2-651 | DWRC-1260 | T.durum | Landrace | 9.99E-05 | 9.99E-05 | 9.99E-05 | 9.99E-05 | 9.99E-05 | 0.999201 | 9.99E-05 | 9.99E-05 | 9.99E-05 | EEU_LND |
| UKR_GDPv2-652 | DWRC-1261 | T.durum | Landrace | 1.00E-04 | 1.00E-04 | 1.00E-04 | 1.00E-04 | 0.018418 | 0.846308 | 0.076628 | 0.058147 | 1.00E-04 | EEU_LND |
| IRQ_GDPv2-666 | DWRC-1287 | T.durum | Landrace | 9.99E-05 | 9.99E-05 | 9.99E-05 | 9.99E-05 | 0.208195 | 0.791105 | 9.99E-05 | 9.99E-05 | 9.99E-05 | EEU_LND |
| SRB_GDPv2-667 | DWRC-1288 | T.durum | Landrace | 0.027202 | 1.00E-04 | 0.033966 | 1.00E-04 | 0.037898 | 0.900434 | 1.00E-04 | 1.00E-04 | 1.00E-04 | EEU_LND |
| USA_GDPv2-668 | DWRC-1294 | T.durum | Landrace | 1.00E-04 | 0.007451 | 1.00E-04 | 0.012424 | 0.410552 | 0.49417 | 0.043301 | 0.030965 | 0.000937 | EEU_LND |
| CYP_GDPv2-685 | DWRC-1321 | T.durum | Landrace | 0.003507 | 1.00E-04 | 1.00E-04 | 1.00E-04 | 0.319147 | 0.326736 | 0.301607 | 0.048603 | 1.00E-04 | EEU_LND |
| AFG_GDPv2-686 | DWRC-1322 | T.durum | Landrace | 1.00E-04 | 1.00E-04 | 1.00E-04 | 1.00E-04 | 0.107905 | 0.689588 | 0.157925 | 0.044082 | 1.00E-04 | EEU_LND |
| IRN_GDPv2-688 | DWRC-1324 | T.durum | Landrace | 1.00E-04 | 1.00E-04 | 1.00E-04 | 1.00E-04 | 0.360712 | 0.52448 | 0.06765 | 0.046659 | 1.00E-04 | EEU_LND |
| BGR_GDPv2-692 | DWRC-1329 | T.durum | Landrace | 1.00E-04 | 1.00E-04 | 0.019176 | 0.004178 | 0.397029 | 0.516924 | 0.017426 | 0.044966 | 1.00E-04 | EEU_LND |
| GRC_GDPv2-703 | DWRC-1354 | T.durum | Landrace | 0.022604 | 1.00E-04 | 1.00E-04 | 1.00E-04 | 0.246949 | 0.469017 | 0.220174 | 0.040856 | 1.00E-04 | EEU_LND |
| GRC_GDPv2-704 | DWRC-1355 | T.durum | Landrace | 0.019526 | 1.00E-04 | 1.00E-04 | 0.016136 | 1.00E-04 | 0.556754 | 0.291681 | 0.113777 | 0.001827 | EEU_LND |
| GRC_GDPv2-705 | DWRC-1357 | T.durum | Landrace | 0.010594 | 1.00E-04 | 1.00E-04 | 1.00E-04 | 0.234221 | 0.327046 | 0.206586 | 0.221154 | 1.00E-04 | EEU_LND |
| GRC_GDPv2-708 | DWRC-1361 | T.durum | Landrace | 0.029935 | 1.00E-04 | 1.00E-04 | 1.00E-04 | 0.378707 | 0.291761 | 0.136726 | 0.162471 | 1.00E-04 | EEU_LND |
| GRC_GDPv2-709 | DWRC-1366 | T.durum | Landrace | 0.023646 | 0.012476 | 1.00E-04 | 0.019018 | 0.252449 | 0.318053 | 0.099664 | 0.274495 | 1.00E-04 | EEU_LND |
| ITA_GDPv2-716 | DWRC-1378 | T.durum | Landrace | 1.00E-04 | 0.01121 | 1.00E-04 | 1.00E-04 | 0.051762 | 0.474329 | 0.29576 | 0.166539 | 1.00E-04 | EEU_LND |
| GRC_GDPv2-719 | DWRC-1383 | T.durum | Landrace | 0.017025 | 0.019153 | 1.00E-04 | 1.00E-04 | 0.269906 | 0.308029 | 0.130202 | 0.255384 | 1.00E-04 | EEU_LND |
| RUS_GDPv2-757 | DWRC-2213 | T.durum | Landrace | 9.99E-05 | 9.99E-05 | 9.99E-05 | 9.99E-05 | 9.99E-05 | 0.999201 | 9.99E-05 | 9.99E-05 | 9.99E-05 | EEU_LND |
| TUR_GDPv2-767 | DWRC-1923 | T.turanicum | Landrace | 1.00E-04 | 1.00E-04 | 1.00E-04 | 0.013089 | 0.416202 | 0.445579 | 0.025162 | 0.099568 | 1.00E-04 | EEU_LND |
| GEO_GDPv2-783 | DWRC-1950 | T.polonicum | Landrace | 1.00E-04 | 1.00E-04 | 1.00E-04 | 1.00E-04 | 0.291297 | 0.521963 | 0.110463 | 0.075777 | 1.00E-04 | EEU_LND |
| ITA_GDPv2-784 | DWRC-1952 | T.polonicum | Landrace | 1.00E-04 | 1.00E-04 | 1.00E-04 | 0.016861 | 0.282278 | 0.334584 | 0.113322 | 0.252555 | 1.00E-04 | EEU_LND |
| LBN_GDPv2-843 | DWRC-1485 | T.dicoccoides | W_Emmer | 0.210365 | 0.075102 | 0.026999 | 1.00E-04 | 0.058873 | 0.578064 | 0.050297 | 1.00E-04 | 1.00E-04 | EEU_LND |
| MAR_GDPv2-894 | DWRC-1570 | T.dicoccum | D_Emmer | 0.034424 | 0.292501 | 0.113646 | 1.00E-04 | 1.00E-04 | 0.42168 | 0.017912 | 0.089761 | 0.029876 | EEU_LND |
| TUR_GDPv2-1006 | DWRC-1484 | T.dicoccoides | W_Emmer | 9.99E-05 | 0.999201 | 9.99E-05 | 9.99E-05 | 9.99E-05 | 9.99E-05 | 9.99E-05 | 9.99E-05 | 9.99E-05 | EUMED_DOM |
| TUR_GDPv2-827 | DWRC-1454 | T.dicoccoides | W_Emmer | 1.00E-04 | 0.4883 | 0.03851 | 1.00E-04 | 0.15517 | 0.069192 | 0.008083 | 0.240446 | 1.00E-04 | EUMED_DOM |
| TUR_GDPv2-830 | DWRC-1458 | T.dicoccoides | W_Emmer | 9.99E-05 | 0.905688 | 0.035375 | 9.99E-05 | 9.99E-05 | 9.99E-05 | 9.99E-05 | 0.058337 | 9.99E-05 | EUMED_DOM |
| TUR_GDPv2-842 | DWRC-1483 | T.dicoccoides | W_Emmer | 0.028593 | 0.581283 | 0.269014 | 1.00E-04 | 0.028091 | 1.00E-04 | 1.00E-04 | 0.054602 | 0.038115 | EUMED_DOM |
| na_GDPv2-848 | DWRC-1495 | T.dicoccoides | W_Emmer | 0.025271 | 0.548818 | 0.069189 | 1.00E-04 | 1.00E-04 | 1.00E-04 | 1.00E-04 | 0.325742 | 0.030581 | EUMED_DOM |
| na_GDPv2-849 | DWRC-1497 | T.dicoccoides | W_Emmer | 0.025587 | 0.554766 | 0.069263 | 1.00E-04 | 1.00E-04 | 1.00E-04 | 1.00E-04 | 0.31902 | 0.030964 | EUMED_DOM |
| na_GDPv2-853 | DWRC-2009 | T.dicoccum | D_Emmer | 9.99E-05 | 0.999201 | 9.99E-05 | 9.99E-05 | 9.99E-05 | 9.99E-05 | 9.99E-05 | 9.99E-05 | 9.99E-05 | EUMED_DOM |
| ARM_GDPv2-857 | DWRC-2015 | T.dicoccum | D_Emmer | 0.068735 | 0.327115 | 0.176937 | 1.00E-04 | 0.085839 | 1.00E-04 | 1.00E-04 | 0.060588 | 0.280486 | EUMED_DOM |
| TUR_GDPv2-869 | DWRC-2497 | T.dicoccoides | W_Emmer | 9.99E-05 | 0.989634 | 9.99E-05 | 9.99E-05 | 9.99E-05 | 9.99E-05 | 0.006107 | 9.99E-05 | 0.003659 | EUMED_DOM |
| na_GDPv2-874 | DWRC-1431 | T.dicoccum | D_Emmer | 0.004189 | 0.830422 | 1.00E-04 | 1.00E-04 | 1.00E-04 | 0.012798 | 0.041773 | 0.084492 | 0.026026 | EUMED_DOM |
| ITA_GDPv2-876 | DWRC-1530 | T.dicoccum | D_Emmer | 0.050934 | 0.799346 | 0.00828 | 1.00E-04 | 0.017559 | 1.00E-04 | 1.00E-04 | 0.107046 | 0.016536 | EUMED_DOM |
| ITA_GDPv2-877 | DWRC-1532 | T.dicoccum | D_Emmer | 0.010956 | 0.829931 | 0.025647 | 1.00E-04 | 1.00E-04 | 1.00E-04 | 1.00E-04 | 0.12242 | 0.010647 | EUMED_DOM |
| ITA_GDPv2-878 | DWRC-1534 | T.dicoccum | D_Emmer | 0.027196 | 0.829902 | 0.021963 | 1.00E-04 | 0.000643 | 0.001558 | 1.00E-04 | 0.113566 | 0.004972 | EUMED_DOM |
| ITA_GDPv2-879 | DWRC-1536 | T.dicoccum | D_Emmer | 0.004789 | 0.841169 | 0.011992 | 0.006411 | 1.00E-04 | 0.002178 | 1.00E-04 | 0.111492 | 0.021769 | EUMED_DOM |
| ITA_GDPv2-880 | DWRC-1537 | T.dicoccum | D_Emmer | 0.022113 | 0.847012 | 1.00E-04 | 0.008955 | 1.00E-04 | 1.00E-04 | 1.00E-04 | 0.102649 | 0.018871 | EUMED_DOM |
| ITA_GDPv2-881 | DWRC-1538 | T.dicoccum | D_Emmer | 0.035248 | 0.797192 | 0.044734 | 1.00E-04 | 1.00E-04 | 1.00E-04 | 1.00E-04 | 0.111101 | 0.011325 | EUMED_DOM |
| KEN_GDPv2-882 | DWRC-1540 | T.dicoccum | D_Emmer | 0.009044 | 0.84062 | 0.005813 | 0.007009 | 1.00E-04 | 0.005247 | 1.00E-04 | 0.108719 | 0.023349 | EUMED_DOM |
| UK_GDPv2-886 | DWRC-1546 | T.dicoccum | D_Emmer | 0.041696 | 0.502321 | 0.237127 | 0.008014 | 0.071856 | 0.014969 | 1.00E-04 | 0.082129 | 0.041789 | EUMED_DOM |
| UK_GDPv2-887 | DWRC-1547 | T.dicoccum | D_Emmer | 1.00E-04 | 0.900762 | 0.054865 | 1.00E-04 | 0.011703 | 1.00E-04 | 0.010444 | 0.021826 | 1.00E-04 | EUMED_DOM |
| BGR_GDPv2-897 | DWRC-1574 | T.dicoccum | D_Emmer | 0.025255 | 0.530236 | 0.414982 | 1.00E-04 | 1.00E-04 | 1.00E-04 | 0.029027 | 1.00E-04 | 1.00E-04 | EUMED_DOM |
| DEU_GDPv2-898 | DWRC-1578 | T.dicoccum | D_Emmer | 0.007016 | 0.552744 | 0.259464 | 0.032941 | 0.005352 | 1.00E-04 | 1.00E-04 | 0.074263 | 0.068021 | EUMED_DOM |
| RUS_GDPv2-899 | DWRC-1581 | T.dicoccum | D_Emmer | 1.00E-04 | 0.904514 | 0.037005 | 1.00E-04 | 1.00E-04 | 1.00E-04 | 1.00E-04 | 0.056049 | 0.001932 | EUMED_DOM |
| IND_GDPv2-904 | DWRC-1590 | T.dicoccum | D_Emmer | 0.220896 | 0.357804 | 0.056248 | 1.00E-04 | 0.181474 | 1.00E-04 | 0.133771 | 0.049507 | 1.00E-04 | EUMED_DOM |
| ESP_GDPv2-906 | DWRC-1593 | T.dicoccum | D_Emmer | 9.99E-05 | 0.879333 | 0.079535 | 9.99E-05 | 9.99E-05 | 9.99E-05 | 9.99E-05 | 0.040533 | 9.99E-05 | EUMED_DOM |
| ESP_GDPv2-907 | DWRC-1595 | T.dicoccum | D_Emmer | 0.032885 | 0.546456 | 0.275244 | 0.004947 | 0.023521 | 1.00E-04 | 1.00E-04 | 0.059281 | 0.057465 | EUMED_DOM |
| ETH_GDPv2-908 | DWRC-1597 | T.dicoccum | D_Emmer | 0.456003 | 0.497813 | 0.035498 | 0.000311 | 1.00E-04 | 1.00E-04 | 1.00E-04 | 1.00E-04 | 0.009976 | EUMED_DOM |
| ETH_GDPv2-909 | DWRC-1602 | T.dicoccum | D_Emmer | 9.99E-05 | 0.999201 | 9.99E-05 | 9.99E-05 | 9.99E-05 | 9.99E-05 | 9.99E-05 | 9.99E-05 | 9.99E-05 | EUMED_DOM |
| ETH_GDPv2-910 | DWRC-1603 | T.dicoccum | D_Emmer | 9.99E-05 | 0.999201 | 9.99E-05 | 9.99E-05 | 9.99E-05 | 9.99E-05 | 9.99E-05 | 9.99E-05 | 9.99E-05 | EUMED_DOM |
| ETH_GDPv2-911 | DWRC-1604 | T.dicoccum | D_Emmer | 0.391221 | 0.467563 | 0.09149 | 1.00E-04 | 0.010393 | 0.032462 | 1.00E-04 | 1.00E-04 | 0.006572 | EUMED_DOM |
| SRB_GDPv2-919 | DWRC-1623 | T.dicoccum | D_Emmer | 0.033347 | 0.664687 | 0.129033 | 1.00E-04 | 1.00E-04 | 1.00E-04 | 1.00E-04 | 0.145102 | 0.02743 | EUMED_DOM |
| DEU_GDPv2-922 | DWRC-1641 | T.dicoccum | D_Emmer | 0.014928 | 0.365729 | 0.326716 | 0.013725 | 0.046397 | 0.064301 | 0.041951 | 0.063274 | 0.062979 | EUMED_DOM |
| DEU_GDPv2-923 | DWRC-1642 | T.dicoccum | D_Emmer | 1.00E-04 | 0.75003 | 0.107487 | 1.00E-04 | 0.047854 | 0.069821 | 0.024408 | 1.00E-04 | 1.00E-04 | EUMED_DOM |
| IRN_GDPv2-924 | DWRC-1644 | T.dicoccum | D_Emmer | 0.035239 | 0.559578 | 0.404583 | 9.99E-05 | 9.99E-05 | 9.99E-05 | 9.99E-05 | 9.99E-05 | 9.99E-05 | EUMED_DOM |
| HUN_GDPv2-929 | DWRC-1663 | T.dicoccum | D_Emmer | 9.99E-05 | 0.995674 | 9.99E-05 | 9.99E-05 | 9.99E-05 | 9.99E-05 | 0.003627 | 9.99E-05 | 9.99E-05 | EUMED_DOM |
| HUN_GDPv2-930 | DWRC-1664 | T.dicoccum | D_Emmer | 9.99E-05 | 0.999201 | 9.99E-05 | 9.99E-05 | 9.99E-05 | 9.99E-05 | 9.99E-05 | 9.99E-05 | 9.99E-05 | EUMED_DOM |
| HUN_GDPv2-931 | DWRC-1665 | T.dicoccum | D_Emmer | 9.99E-05 | 0.999201 | 9.99E-05 | 9.99E-05 | 9.99E-05 | 9.99E-05 | 9.99E-05 | 9.99E-05 | 9.99E-05 | EUMED_DOM |
| HUN_GDPv2-932 | DWRC-1666 | T.dicoccum | D_Emmer | 9.99E-05 | 0.999201 | 9.99E-05 | 9.99E-05 | 9.99E-05 | 9.99E-05 | 9.99E-05 | 9.99E-05 | 9.99E-05 | EUMED_DOM |
| HUN_GDPv2-933 | DWRC-1667 | T.dicoccum | D_Emmer | 0.035628 | 0.436755 | 0.242738 | 1.00E-04 | 0.130567 | 0.007732 | 0.065006 | 0.05262 | 0.028856 | EUMED_DOM |
| ESP_GDPv2-934 | DWRC-1674 | T.dicoccum | D_Emmer | 1.00E-04 | 0.937196 | 0.04732 | 0.001446 | 0.008444 | 1.00E-04 | 0.005195 | 1.00E-04 | 1.00E-04 | EUMED_DOM |
| ESP_GDPv2-935 | DWRC-1677 | T.dicoccum | D_Emmer | 9.99E-05 | 0.910823 | 9.99E-05 | 9.99E-05 | 9.99E-05 | 9.99E-05 | 9.99E-05 | 0.066927 | 0.02165 | EUMED_DOM |
| ESP_GDPv2-937 | DWRC-1680 | T.dicoccum | D_Emmer | 1.00E-04 | 0.862243 | 1.00E-04 | 0.001213 | 0.009198 | 1.00E-04 | 0.009127 | 0.108636 | 0.009282 | EUMED_DOM |
| ESP_GDPv2-938 | DWRC-1682 | T.dicoccum | D_Emmer | 9.99E-05 | 0.997701 | 9.99E-05 | 9.99E-05 | 9.99E-05 | 9.99E-05 | 0.0016 | 9.99E-05 | 9.99E-05 | EUMED_DOM |
| ESP_GDPv2-939 | DWRC-1686 | T.dicoccum | D_Emmer | 9.99E-05 | 0.999103 | 9.99E-05 | 9.99E-05 | 0.000197 | 9.99E-05 | 9.99E-05 | 9.99E-05 | 9.99E-05 | EUMED_DOM |
| RUS_GDPv2-945 | DWRC-1700 | T.dicoccum | D_Emmer | 9.99E-05 | 0.991446 | 9.99E-05 | 9.99E-05 | 9.99E-05 | 9.99E-05 | 0.007855 | 9.99E-05 | 9.99E-05 | EUMED_DOM |
| RUS_GDPv2-946 | DWRC-1701 | T.dicoccum | D_Emmer | 0.009432 | 0.948655 | 0.026971 | 0.00782 | 1.00E-04 | 1.00E-04 | 0.006723 | 1.00E-04 | 1.00E-04 | EUMED_DOM |
| CHN_GDPv2-949 | DWRC-1707 | T.dicoccum | D_Emmer | 9.99E-05 | 0.572258 | 0.424092 | 9.99E-05 | 9.99E-05 | 9.99E-05 | 0.00305 | 9.99E-05 | 9.99E-05 | EUMED_DOM |
| YUG_GDPv2-955 | DWRC-1716 | T.dicoccum | D_Emmer | 0.071283 | 0.397836 | 0.334045 | 1.00E-04 | 1.00E-04 | 1.00E-04 | 0.035761 | 0.091844 | 0.068931 | EUMED_DOM |
| IRN_GDPv2-956 | DWRC-1717 | T.dicoccum | D_Emmer | 0.158983 | 0.682588 | 0.059889 | 1.00E-04 | 1.00E-04 | 1.00E-04 | 1.00E-04 | 0.098041 | 1.00E-04 | EUMED_DOM |
| ESP_GDPv2-960 | DWRC-1991 | T.dicoccum | D_Emmer | 9.99E-05 | 0.999201 | 9.99E-05 | 9.99E-05 | 9.99E-05 | 9.99E-05 | 9.99E-05 | 9.99E-05 | 9.99E-05 | EUMED_DOM |
| ITA_GDPv2-961 | DWRC-1992 | T.dicoccum | D_Emmer | 9.99E-05 | 0.91971 | 0.024516 | 9.99E-05 | 9.99E-05 | 9.99E-05 | 9.99E-05 | 0.055174 | 9.99E-05 | EUMED_DOM |
| ITA_GDPv2-962 | DWRC-1993 | T.dicoccum | D_Emmer | 9.99E-05 | 0.902547 | 0.03478 | 9.99E-05 | 9.99E-05 | 9.99E-05 | 9.99E-05 | 0.062073 | 9.99E-05 | EUMED_DOM |
| IRN_GDPv2-963 | DWRC-1995 | T.dicoccum | D_Emmer | 1.00E-04 | 0.625839 | 0.356506 | 1.00E-04 | 1.00E-04 | 0.007321 | 1.00E-04 | 0.009834 | 1.00E-04 | EUMED_DOM |
| ESP_GDPv2-964 | DWRC-1996 | T.dicoccum | D_Emmer | 9.99E-05 | 0.966002 | 0.02209 | 9.99E-05 | 0.011308 | 9.99E-05 | 9.99E-05 | 9.99E-05 | 9.99E-05 | EUMED_DOM |
| SYR_GDPv2-965 | DWRC-1997 | T.dicoccum | D_Emmer | 1.00E-04 | 0.492458 | 0.036524 | 1.00E-04 | 0.156158 | 0.065058 | 0.015501 | 0.234001 | 1.00E-04 | EUMED_DOM |
| ETH_GDPv2-966 | DWRC-1998 | T.dicoccum | D_Emmer | 9.99E-05 | 0.999201 | 9.99E-05 | 9.99E-05 | 9.99E-05 | 9.99E-05 | 9.99E-05 | 9.99E-05 | 9.99E-05 | EUMED_DOM |
| ITA_GDPv2-967 | DWRC-2000 | T.dicoccum | D_Emmer | 9.99E-05 | 0.999201 | 9.99E-05 | 9.99E-05 | 9.99E-05 | 9.99E-05 | 9.99E-05 | 9.99E-05 | 9.99E-05 | EUMED_DOM |
| ITA_GDPv2-968 | DWRC-2001 | T.dicoccum | D_Emmer | 0.026305 | 0.851478 | 1.00E-04 | 0.006715 | 1.00E-04 | 1.00E-04 | 1.00E-04 | 0.09621 | 0.018892 | EUMED_DOM |
| MAR_GDPv2-969 | DWRC-2003 | T.dicoccum | D_Emmer | 0.035309 | 0.814739 | 1.00E-04 | 1.00E-04 | 1.00E-04 | 0.013461 | 0.018541 | 0.117549 | 1.00E-04 | EUMED_DOM |
| UK_GDPv2-970 | DWRC-2004 | T.dicoccum | D_Emmer | 1.00E-04 | 0.844553 | 1.00E-04 | 0.005126 | 0.018032 | 1.00E-04 | 0.025342 | 0.106548 | 1.00E-04 | EUMED_DOM |
| HUN_GDPv2-972 | DWRC-2006 | T.dicoccum | D_Emmer | 0.082964 | 0.83169 | 1.00E-04 | 1.00E-04 | 1.00E-04 | 0.034636 | 0.05021 | 1.00E-04 | 1.00E-04 | EUMED_DOM |
| na_GDPv2-976 | DWRC-2148 | T.dicoccum | D_Emmer | 9.99E-05 | 0.999201 | 9.99E-05 | 9.99E-05 | 9.99E-05 | 9.99E-05 | 9.99E-05 | 9.99E-05 | 9.99E-05 | EUMED_DOM |
| TUR_GDPv2-560 | DWRC-1011 | T.durum | Landrace | 0.003884 | 0.138305 | 1.00E-04 | 1.00E-04 | 0.13652 | 0.106895 | 0.027416 | 0.553673 | 0.033106 | EUMED_LND |
| ESP_GDPv2-620 | DWRC-1213 | T.durum | Landrace | 0.01892 | 1.00E-04 | 1.00E-04 | 0.014575 | 0.179383 | 0.128407 | 0.308573 | 0.349842 | 1.00E-04 | EUMED_LND |
| YUG_GDPv2-621 | DWRC-1216 | T.durum | Landrace | 0.009102 | 0.114301 | 1.00E-04 | 1.00E-04 | 0.046661 | 0.089245 | 0.080356 | 0.660036 | 1.00E-04 | EUMED_LND |
| TUR_GDPv2-669 | DWRC-1297 | T.durum | Landrace | 1.00E-04 | 1.00E-04 | 1.00E-04 | 0.015617 | 0.19976 | 0.155605 | 0.300575 | 0.328043 | 1.00E-04 | EUMED_LND |
| GRC_GDPv2-717 | DWRC-1379 | T.durum | Landrace | 0.025813 | 0.109898 | 1.00E-04 | 1.00E-04 | 0.042051 | 0.044753 | 0.11961 | 0.63201 | 0.025665 | EUMED_LND |
| ITA_GDPv2-726 | DWRC-1403 | T.turgidum | Landrace | 1.00E-04 | 0.048284 | 0.094649 | 0.228952 | 0.067284 | 0.051178 | 0.010696 | 0.307829 | 0.191028 | EUMED_LND |
| ITA_GDPv2-727 | DWRC-1404 | T.durum | Landrace | 1.00E-04 | 0.108068 | 0.089644 | 0.150219 | 0.049737 | 0.059466 | 0.054064 | 0.282745 | 0.205958 | EUMED_LND |
| ITA_GDPv2-732 | DWRC-1411 | T.durum | Landrace | 1.00E-04 | 1.00E-04 | 0.116144 | 0.199283 | 0.081609 | 0.103788 | 0.113841 | 0.232633 | 0.152502 | EUMED_LND |
| PRT_GDPv2-746 | DWRC-2026 | T.durum | Landrace | 1.00E-04 | 1.00E-04 | 1.00E-04 | 1.00E-04 | 0.050905 | 0.007885 | 0.040311 | 0.900399 | 1.00E-04 | EUMED_LND |
| UK_GDPv2-779 | DWRC-1945 | T.polonicum | Landrace | 1.00E-04 | 0.000843 | 0.025815 | 1.00E-04 | 0.123399 | 0.06882 | 0.013669 | 0.767154 | 1.00E-04 | EUMED_LND |
| ROU_GDPv2-781 | DWRC-1947 | T.polonicum | Landrace | 1.00E-04 | 1.00E-04 | 0.028577 | 1.00E-04 | 0.13776 | 0.096447 | 0.007831 | 0.728985 | 1.00E-04 | EUMED_LND |
| PRT_GDPv2-788 | DWRC-1958 | T.turgidum | Landrace | 0.026858 | 0.034822 | 1.00E-04 | 1.00E-04 | 1.00E-04 | 0.031234 | 0.022011 | 0.867801 | 0.016974 | EUMED_LND |
| ITA_GDPv2-789 | DWRC-1960 | T.turgidum | Landrace | 1.00E-04 | 0.046092 | 1.00E-04 | 0.019682 | 1.00E-04 | 0.099753 | 0.128834 | 0.705239 | 1.00E-04 | EUMED_LND |
| ITA_GDPv2-790 | DWRC-1961 | T.turgidum | Landrace | 1.00E-04 | 0.075469 | 1.00E-04 | 0.051732 | 1.00E-04 | 0.059455 | 0.095592 | 0.717351 | 1.00E-04 | EUMED_LND |
| PRT_GDPv2-792 | DWRC-1963 | T.turgidum | Landrace | 9.99E-05 | 9.99E-05 | 9.99E-05 | 9.99E-05 | 0.015399 | 9.99E-05 | 9.99E-05 | 0.983901 | 9.99E-05 | EUMED_LND |
| ESP_GDPv2-794 | DWRC-1965 | T.turgidum | Landrace | 1.00E-04 | 0.02922 | 1.00E-04 | 1.00E-04 | 0.164544 | 0.261552 | 0.259148 | 0.285137 | 1.00E-04 | EUMED_LND |
| DEU_GDPv2-796 | DWRC-1970 | T.turgidum | Landrace | 9.99E-05 | 0.026101 | 0.017884 | 9.99E-05 | 9.99E-05 | 9.99E-05 | 9.99E-05 | 0.955415 | 9.99E-05 | EUMED_LND |
| TUR_GDPv2-797 | DWRC-1972 | T.turgidum | Landrace | 1.00E-04 | 0.072333 | 0.024214 | 0.01215 | 1.00E-04 | 1.00E-04 | 1.00E-04 | 0.890803 | 1.00E-04 | EUMED_LND |
| UK_GDPv2-798 | DWRC-1973 | T.turgidum | Landrace | 1.00E-04 | 0.098913 | 0.020812 | 0.003183 | 1.00E-04 | 1.00E-04 | 1.00E-04 | 0.876592 | 1.00E-04 | EUMED_LND |
| DEU_GDPv2-799 | DWRC-1974 | T.turgidum | Landrace | 9.99E-05 | 9.99E-05 | 9.99E-05 | 9.99E-05 | 0.014214 | 9.99E-05 | 9.99E-05 | 0.985086 | 9.99E-05 | EUMED_LND |
| RUS_GDPv2-800 | DWRC-1977 | T.carthlicum | Landrace | 1.00E-04 | 0.087152 | 1.00E-04 | 0.01226 | 1.00E-04 | 1.00E-04 | 0.014097 | 0.885992 | 1.00E-04 | EUMED_LND |
| PRT_GDPv2-816 | DWRC-2024 | T.turgidum | Landrace | 9.99E-05 | 9.99E-05 | 9.99E-05 | 9.99E-05 | 0.0136 | 9.99E-05 | 9.99E-05 | 0.9857 | 9.99E-05 | EUMED_LND |
| na_GDPv2-847 | DWRC-1494 | T.dicoccoides | W_Emmer | 1.00E-04 | 1.00E-04 | 0.026094 | 1.00E-04 | 0.129065 | 0.066482 | 0.008735 | 0.769224 | 1.00E-04 | EUMED_LND |
| ROU_GDPv2-941 | DWRC-1692 | T.dicoccum | D_Emmer | 0.094284 | 0.376368 | 0.048148 | 1.00E-04 | 1.00E-04 | 1.00E-04 | 1.00E-04 | 0.4807 | 1.00E-04 | EUMED_LND |
| DEU_GDPv2-953 | DWRC-1713 | T.dicoccum | D_Emmer | 0.087164 | 0.384781 | 1.00E-04 | 1.00E-04 | 1.00E-04 | 0.015357 | 1.00E-04 | 0.512198 | 1.00E-04 | EUMED_LND |
| EGY_GDPv2-023 | DWRC-0100 | T.durum | Landrace | 0.00573 | 1.00E-04 | 1.00E-04 | 1.00E-04 | 1.00E-04 | 0.08749 | 0.788935 | 0.117345 | 1.00E-04 | MED_LND |
| ARG_GDPv2-032 | DWRC-0131 | T.durum | Landrace | 9.99E-05 | 9.99E-05 | 9.99E-05 | 0.008898 | 9.99E-05 | 0.003 | 0.987503 | 9.99E-05 | 9.99E-05 | MED_LND |
| ARG_GDPv2-033 | DWRC-0132 | T.durum | Landrace | 9.99E-05 | 9.99E-05 | 9.99E-05 | 9.99E-05 | 9.99E-05 | 9.99E-05 | 0.999201 | 9.99E-05 | 9.99E-05 | MED_LND |
| ARG_GDPv2-060 | DWRC-0230 | T.durum | Landrace | 9.99E-05 | 9.99E-05 | 9.99E-05 | 9.99E-05 | 9.99E-05 | 9.99E-05 | 0.999201 | 9.99E-05 | 9.99E-05 | MED_LND |
| ITA_GDPv2-091 | DWRC-0285 | T.durum | Landrace | 1.00E-04 | 0.009483 | 1.00E-04 | 0.017066 | 0.044677 | 0.043108 | 0.885266 | 1.00E-04 | 1.00E-04 | MED_LND |
| GRC_GDPv2-285 | DWRC-0999 | T.durum | Landrace | 1.00E-04 | 1.00E-04 | 1.00E-04 | 1.00E-04 | 0.321857 | 0.128023 | 0.530306 | 0.019314 | 1.00E-04 | MED_LND |
| ESP_GDPv2-299 | DWRC-1061 | T.durum | Landrace | 1.00E-04 | 1.00E-04 | 1.00E-04 | 1.00E-04 | 0.06128 | 0.259322 | 0.411795 | 0.26234 | 0.004863 | MED_LND |
| ESP_GDPv2-397 | DWRC-2225 | T.durum | Landrace | 1.00E-04 | 1.00E-04 | 1.00E-04 | 1.00E-04 | 0.052033 | 0.286226 | 0.421482 | 0.23976 | 1.00E-04 | MED_LND |
| ISR_GDPv2-401 | DWRC-2235 | T.durum | Landrace | 0.017016 | 1.00E-04 | 0.029383 | 1.00E-04 | 0.259945 | 0.123415 | 0.484678 | 0.085262 | 1.00E-04 | MED_LND |
| TUN_GDPv2-405 | DWRC-2278 | T.durum | Landrace | 1.00E-04 | 0.001868 | 0.010201 | 0.006252 | 1.00E-04 | 0.32631 | 0.635293 | 0.019776 | 1.00E-04 | MED_LND |
| JOR_GDPv2-556 | DWRC-1007 | T.durum | Landrace | 0.005941 | 1.00E-04 | 0.047692 | 0.014096 | 0.197063 | 0.150653 | 0.58413 | 1.00E-04 | 0.000225 | MED_LND |
| TUR_GDPv2-562 | DWRC-1014 | T.durum | Landrace | 1.00E-04 | 1.00E-04 | 1.00E-04 | 0.010568 | 0.14045 | 0.173579 | 0.574663 | 0.10034 | 1.00E-04 | MED_LND |
| TUR_GDPv2-563 | DWRC-1015 | T.durum | Landrace | 9.99E-05 | 9.99E-05 | 9.99E-05 | 9.99E-05 | 0.284463 | 9.99E-05 | 0.714838 | 9.99E-05 | 9.99E-05 | MED_LND |
| ESP_GDPv2-564 | DWRC-1024 | T.durum | Landrace | 1.00E-04 | 1.00E-04 | 1.00E-04 | 0.00246 | 1.00E-04 | 0.183999 | 0.740772 | 0.072269 | 1.00E-04 | MED_LND |
| ISR_GDPv2-568 | DWRC-1038 | T.durum | Landrace | 1.00E-04 | 1.00E-04 | 1.00E-04 | 0.007255 | 0.353951 | 0.133062 | 0.480134 | 0.025198 | 1.00E-04 | MED_LND |
| TUN_GDPv2-569 | DWRC-1040 | T.durum | Landrace | 9.99E-05 | 9.99E-05 | 9.99E-05 | 9.99E-05 | 9.99E-05 | 9.99E-05 | 0.99234 | 9.99E-05 | 0.006961 | MED_LND |
| JOR_GDPv2-581 | DWRC-1153 | T.durum | Landrace | 1.00E-04 | 0.008743 | 1.00E-04 | 1.00E-04 | 1.00E-04 | 0.205656 | 0.745685 | 0.039416 | 1.00E-04 | MED_LND |
| DZA_GDPv2-589 | DWRC-1163 | T.durum | Landrace | 1.00E-04 | 1.00E-04 | 1.00E-04 | 1.00E-04 | 0.039056 | 0.352916 | 0.562918 | 0.044611 | 1.00E-04 | MED_LND |
| RUS_GDPv2-593 | DWRC-1168 | T.durum | Landrace | 0.010592 | 0.02179 | 1.00E-04 | 1.00E-04 | 1.00E-04 | 0.298498 | 0.497726 | 0.170995 | 1.00E-04 | MED_LND |
| ESP_GDPv2-598 | DWRC-1179 | T.durum | Landrace | 1.00E-04 | 1.00E-04 | 1.00E-04 | 0.015094 | 0.077819 | 0.067325 | 0.792001 | 0.025721 | 0.021741 | MED_LND |
| ETH_GDPv2-602 | DWRC-1185 | T.durum | Landrace | 9.99E-05 | 9.99E-05 | 9.99E-05 | 9.99E-05 | 9.99E-05 | 9.99E-05 | 0.999201 | 9.99E-05 | 9.99E-05 | MED_LND |
| AFG_GDPv2-603 | DWRC-1186 | T.durum | Landrace | 0.006703 | 1.00E-04 | 1.00E-04 | 0.376927 | 1.00E-04 | 1.00E-04 | 0.598042 | 1.00E-04 | 0.017828 | MED_LND |
| TUR_GDPv2-606 | DWRC-1195 | T.durum | Landrace | 1.00E-04 | 1.00E-04 | 1.00E-04 | 1.00E-04 | 0.071945 | 0.382742 | 0.49694 | 0.047872 | 1.00E-04 | MED_LND |
| AZE_GDPv2-618 | DWRC-1211 | T.durum | Landrace | 1.00E-04 | 0.011671 | 1.00E-04 | 1.00E-04 | 0.060776 | 0.179662 | 0.625803 | 0.121689 | 1.00E-04 | MED_LND |
| ETH_GDPv2-624 | DWRC-1220 | T.durum | Landrace | 9.99E-05 | 9.99E-05 | 9.99E-05 | 0.234919 | 0.043246 | 9.99E-05 | 0.721235 | 9.99E-05 | 9.99E-05 | MED_LND |
| ITA_GDPv2-626 | DWRC-1223 | T.durum | Landrace | 9.99E-05 | 9.99E-05 | 9.99E-05 | 9.99E-05 | 9.99E-05 | 9.99E-05 | 0.999201 | 9.99E-05 | 9.99E-05 | MED_LND |
| ITA_GDPv2-628 | DWRC-1225 | T.durum | Landrace | 1.00E-04 | 1.00E-04 | 1.00E-04 | 1.00E-04 | 0.016477 | 0.178284 | 0.750434 | 0.05369 | 0.000715 | MED_LND |
| GRC_GDPv2-629 | DWRC-1227 | T.durum | Landrace | 9.99E-05 | 9.99E-05 | 9.99E-05 | 9.99E-05 | 0.24899 | 0.097983 | 0.652427 | 9.99E-05 | 9.99E-05 | MED_LND |
| ETH_GDPv2-630 | DWRC-1228 | T.durum | Landrace | 9.99E-05 | 9.99E-05 | 9.99E-05 | 0.117017 | 9.99E-05 | 9.99E-05 | 0.882284 | 9.99E-05 | 9.99E-05 | MED_LND |
| ITA_GDPv2-631 | DWRC-1229 | T.durum | Landrace | 9.99E-05 | 9.99E-05 | 9.99E-05 | 9.99E-05 | 9.99E-05 | 0.381961 | 0.382541 | 0.234898 | 9.99E-05 | MED_LND |
| PAK_GDPv2-632 | DWRC-1230 | T.durum | Landrace | 1.00E-04 | 0.02883 | 0.006057 | 1.00E-04 | 0.300973 | 0.005126 | 0.653181 | 0.005533 | 1.00E-04 | MED_LND |
| TUN_GDPv2-635 | DWRC-1235 | T.durum | Landrace | 0.020237 | 1.00E-04 | 1.00E-04 | 1.00E-04 | 0.151563 | 0.077979 | 0.696717 | 0.044506 | 0.008698 | MED_LND |
| TUN_GDPv2-636 | DWRC-1238 | T.durum | Landrace | 0.017264 | 1.00E-04 | 0.007834 | 1.00E-04 | 0.253628 | 0.102272 | 0.585991 | 0.032712 | 1.00E-04 | MED_LND |
| TUN_GDPv2-637 | DWRC-1239 | T.durum | Landrace | 1.00E-04 | 1.00E-04 | 0.011793 | 0.003273 | 0.004722 | 0.332965 | 0.624433 | 0.022514 | 1.00E-04 | MED_LND |
| DZA_GDPv2-638 | DWRC-1241 | T.durum | Landrace | 0.000515 | 0.001098 | 1.00E-04 | 0.000132 | 1.00E-04 | 0.259072 | 0.703543 | 0.03534 | 1.00E-04 | MED_LND |
| DZA_GDPv2-639 | DWRC-1242 | T.durum | Landrace | 9.99E-05 | 9.99E-05 | 9.99E-05 | 9.99E-05 | 9.99E-05 | 0.362331 | 0.531647 | 0.105422 | 9.99E-05 | MED_LND |
| ESP_GDPv2-641 | DWRC-1244 | T.durum | Landrace | 0.01201 | 0.001079 | 1.00E-04 | 0.013 | 0.038854 | 0.199173 | 0.661375 | 0.074308 | 1.00E-04 | MED_LND |
| DZA_GDPv2-642 | DWRC-1245 | T.durum | Landrace | 1.00E-04 | 1.00E-04 | 1.00E-04 | 0.008878 | 0.039033 | 0.269212 | 0.527584 | 0.154894 | 1.00E-04 | MED_LND |
| TUN_GDPv2-644 | DWRC-1247 | T.durum | Landrace | 1.00E-04 | 1.00E-04 | 1.00E-04 | 1.00E-04 | 0.047223 | 0.332086 | 0.456545 | 0.163646 | 1.00E-04 | MED_LND |
| TUN_GDPv2-646 | DWRC-1250 | T.durum | Landrace | 1.00E-04 | 1.00E-04 | 1.00E-04 | 1.00E-04 | 0.079651 | 0.373323 | 0.379471 | 0.167054 | 1.00E-04 | MED_LND |
| TUN_GDPv2-647 | DWRC-1251 | T.durum | Landrace | 0.002015 | 1.00E-04 | 1.00E-04 | 1.00E-04 | 1.00E-04 | 0.266001 | 0.62416 | 0.107324 | 1.00E-04 | MED_LND |
| TUN_GDPv2-649 | DWRC-1258 | T.durum | Landrace | 1.00E-04 | 1.00E-04 | 1.00E-04 | 1.00E-04 | 0.094498 | 0.371086 | 0.47365 | 0.060266 | 1.00E-04 | MED_LND |
| EGY_GDPv2-654 | DWRC-1263 | T.durum | Landrace | 0.008644 | 1.00E-04 | 0.01704 | 1.00E-04 | 1.00E-04 | 0.079658 | 0.801191 | 0.093067 | 1.00E-04 | MED_LND |
| MAR_GDPv2-655 | DWRC-1265 | T.durum | Landrace | 1.00E-04 | 1.00E-04 | 0.01736 | 1.00E-04 | 1.00E-04 | 0.107298 | 0.764342 | 0.110501 | 1.00E-04 | MED_LND |
| MAR_GDPv2-656 | DWRC-1266 | T.durum | Landrace | 1.00E-04 | 1.00E-04 | 1.00E-04 | 1.00E-04 | 1.00E-04 | 0.11122 | 0.730238 | 0.138344 | 0.019698 | MED_LND |
| MAR_GDPv2-657 | DWRC-1267 | T.durum | Landrace | 1.00E-04 | 1.00E-04 | 0.005874 | 1.00E-04 | 1.00E-04 | 0.109467 | 0.749315 | 0.134844 | 1.00E-04 | MED_LND |
| MAR_GDPv2-658 | DWRC-1268 | T.durum | Landrace | 9.99E-05 | 9.99E-05 | 9.99E-05 | 9.99E-05 | 9.99E-05 | 0.133128 | 0.695383 | 0.17089 | 9.99E-05 | MED_LND |
| MAR_GDPv2-659 | DWRC-1270 | T.durum | Landrace | 1.00E-04 | 1.00E-04 | 1.00E-04 | 1.00E-04 | 1.00E-04 | 0.118408 | 0.743517 | 0.137094 | 0.000482 | MED_LND |
| SAU_GDPv2-680 | DWRC-1314 | T.durum | Landrace | 1.00E-04 | 1.00E-04 | 1.00E-04 | 0.1846 | 0.081857 | 0.003346 | 0.729697 | 1.00E-04 | 1.00E-04 | MED_LND |
| ETH_GDPv2-683 | DWRC-1318 | T.durum | Landrace | 1.00E-04 | 1.00E-04 | 1.00E-04 | 1.00E-04 | 0.041726 | 0.097455 | 0.800655 | 0.059664 | 1.00E-04 | MED_LND |
| TUN_GDPv2-695 | DWRC-1333 | T.durum | Landrace | 1.00E-04 | 0.004233 | 1.00E-04 | 1.00E-04 | 0.098221 | 0.227685 | 0.60638 | 0.063082 | 1.00E-04 | MED_LND |
| TUN_GDPv2-696 | DWRC-1334 | T.durum | Landrace | 1.00E-04 | 0.014554 | 1.00E-04 | 1.00E-04 | 1.00E-04 | 0.055717 | 0.85661 | 0.068623 | 0.004095 | MED_LND |
| LBY_GDPv2-697 | DWRC-1336 | T.durum | Landrace | 1.00E-04 | 0.008507 | 1.00E-04 | 1.00E-04 | 0.034092 | 0.306251 | 0.612856 | 0.037895 | 1.00E-04 | MED_LND |
| IRQ_GDPv2-701 | DWRC-1343 | T.durum | Landrace | 1.00E-04 | 1.00E-04 | 1.00E-04 | 1.00E-04 | 0.223085 | 0.148765 | 0.537966 | 0.089685 | 1.00E-04 | MED_LND |
| GRC_GDPv2-706 | DWRC-1358 | T.durum | Landrace | 1.00E-04 | 1.00E-04 | 1.00E-04 | 0.027689 | 0.068915 | 0.304492 | 0.497785 | 0.10072 | 1.00E-04 | MED_LND |
| GRC_GDPv2-713 | DWRC-1374 | T.durum | Landrace | 9.99E-05 | 9.99E-05 | 9.99E-05 | 9.99E-05 | 0.263007 | 0.119527 | 0.616865 | 9.99E-05 | 9.99E-05 | MED_LND |
| ESP_GDPv2-714 | DWRC-1375 | T.durum | Landrace | 1.00E-04 | 0.009922 | 0.005836 | 1.00E-04 | 0.061419 | 0.2431 | 0.647837 | 0.031585 | 1.00E-04 | MED_LND |
| ITA_GDPv2-715 | DWRC-1376 | T.durum | Landrace | 9.99E-05 | 9.99E-05 | 9.99E-05 | 9.99E-05 | 0.495953 | 9.99E-05 | 0.503347 | 9.99E-05 | 9.99E-05 | MED_LND |
| GRC_GDPv2-718 | DWRC-1380 | T.durum | Landrace | 9.99E-05 | 9.99E-05 | 9.99E-05 | 9.99E-05 | 0.292596 | 0.112974 | 0.59383 | 9.99E-05 | 9.99E-05 | MED_LND |
| ITA_GDPv2-720 | DWRC-1386 | T.durum | Landrace | 9.99E-05 | 9.99E-05 | 9.99E-05 | 9.99E-05 | 9.99E-05 | 9.99E-05 | 0.999201 | 9.99E-05 | 9.99E-05 | MED_LND |
| ITA_GDPv2-722 | DWRC-1398 | T.durum | Landrace | 0.000746 | 1.00E-04 | 0.002276 | 1.00E-04 | 0.036369 | 0.197524 | 0.578374 | 0.184411 | 1.00E-04 | MED_LND |
| ITA_GDPv2-723 | DWRC-1399 | T.turgidum | Landrace | 0.008381 | 1.00E-04 | 1.00E-04 | 1.00E-04 | 0.128493 | 0.318139 | 0.408416 | 0.136171 | 1.00E-04 | MED_LND |
| ITA_GDPv2-724 | DWRC-1401 | T.turgidum | Landrace | 1.00E-04 | 1.00E-04 | 1.00E-04 | 1.00E-04 | 0.070978 | 0.254282 | 0.410881 | 0.258497 | 0.004963 | MED_LND |
| ITA_GDPv2-728 | DWRC-1405 | T.durum | Landrace | 9.99E-05 | 9.99E-05 | 9.99E-05 | 0.000561 | 9.99E-05 | 9.99E-05 | 0.990096 | 0.008744 | 9.99E-05 | MED_LND |
| ITA_GDPv2-729 | DWRC-1407 | T.durum | Landrace | 1.00E-04 | 0.016585 | 0.005426 | 1.00E-04 | 0.041837 | 0.124599 | 0.63454 | 0.176713 | 1.00E-04 | MED_LND |
| ITA_GDPv2-730 | DWRC-1408 | T.durum | Landrace | 0.002777 | 0.024454 | 1.00E-04 | 1.00E-04 | 0.117971 | 0.224973 | 0.485706 | 0.143819 | 1.00E-04 | MED_LND |
| ITA_GDPv2-734 | DWRC-1416 | T.durum | Landrace | 0.019099 | 0.006256 | 1.00E-04 | 1.00E-04 | 0.003214 | 0.265641 | 0.459199 | 0.246291 | 1.00E-04 | MED_LND |
| ITA_GDPv2-735 | DWRC-1417 | T.durum | Landrace | 0.013694 | 1.00E-04 | 1.00E-04 | 1.00E-04 | 1.00E-04 | 0.266671 | 0.508154 | 0.210982 | 1.00E-04 | MED_LND |
| ITA_GDPv2-736 | DWRC-1425 | T.durum | Landrace | 1.00E-04 | 0.002622 | 1.00E-04 | 1.00E-04 | 1.00E-04 | 0.242726 | 0.612261 | 0.141891 | 1.00E-04 | MED_LND |
| ITA_GDPv2-737 | DWRC-1426 | T.durum | Landrace | 1.00E-04 | 1.00E-04 | 0.007941 | 1.00E-04 | 1.00E-04 | 0.055189 | 0.81156 | 0.124809 | 1.00E-04 | MED_LND |
| ITA_GDPv2-749 | DWRC-2120 | T.durum | Landrace | 9.99E-05 | 9.99E-05 | 9.99E-05 | 9.99E-05 | 9.99E-05 | 9.99E-05 | 0.999201 | 9.99E-05 | 9.99E-05 | MED_LND |
| ETH_GDPv2-752 | DWRC-2164 | T.durum | Landrace | 9.99E-05 | 9.99E-05 | 9.99E-05 | 0.354197 | 9.99E-05 | 9.99E-05 | 0.645103 | 9.99E-05 | 9.99E-05 | MED_LND |
| ESP_GDPv2-755 | DWRC-2194 | T.durum | Landrace | 1.00E-04 | 0.002642 | 1.00E-04 | 1.00E-04 | 0.106665 | 0.307724 | 0.370506 | 0.212062 | 1.00E-04 | MED_LND |
| PRT_GDPv2-756 | DWRC-2206 | T.durum | Landrace | 1.00E-04 | 1.00E-04 | 1.00E-04 | 1.00E-04 | 0.035635 | 0.271459 | 0.514997 | 0.177409 | 1.00E-04 | MED_LND |
| ISR_GDPv2-759 | DWRC-2248 | T.durum | Landrace | 1.00E-04 | 0.003965 | 1.00E-04 | 1.00E-04 | 0.320713 | 0.106152 | 0.56867 | 1.00E-04 | 1.00E-04 | MED_LND |
| TUN_GDPv2-762 | DWRC-2285 | T.durum | Landrace | 9.99E-05 | 9.99E-05 | 9.99E-05 | 9.99E-05 | 9.99E-05 | 9.99E-05 | 0.999201 | 9.99E-05 | 9.99E-05 | MED_LND |
| EGY_GDPv2-764 | DWRC-1919 | T.turanicum | Landrace | 0.005172 | 1.00E-04 | 1.00E-04 | 1.00E-04 | 1.00E-04 | 0.096769 | 0.786474 | 0.111085 | 1.00E-04 | MED_LND |
| AZE_GDPv2-775 | DWRC-1937 | T.turanicum | Landrace | 1.00E-04 | 1.00E-04 | 1.00E-04 | 1.00E-04 | 0.133767 | 0.289299 | 0.490848 | 0.085587 | 1.00E-04 | MED_LND |
| JOR_GDPv2-777 | DWRC-1940 | T.polonicum | Landrace | 1.00E-04 | 1.00E-04 | 1.00E-04 | 1.00E-04 | 0.010589 | 0.033537 | 0.867357 | 0.088017 | 1.00E-04 | MED_LND |
| PRT_GDPv2-780 | DWRC-1946 | T.polonicum | Landrace | 1.00E-04 | 1.00E-04 | 1.00E-04 | 1.00E-04 | 0.010401 | 0.033177 | 0.87001 | 0.085912 | 1.00E-04 | MED_LND |
| na_GDPv2-782 | DWRC-1949 | T.polonicum | Landrace | 0.002993 | 1.00E-04 | 1.00E-04 | 1.00E-04 | 0.027418 | 0.054467 | 0.754525 | 0.154047 | 0.00625 | MED_LND |
| IRQ_GDPv2-787 | DWRC-1957 | T.polonicum | Landrace | 1.00E-04 | 1.00E-04 | 1.00E-04 | 1.00E-04 | 0.006646 | 0.026302 | 0.877696 | 0.088857 | 1.00E-04 | MED_LND |
| ESP_GDPv2-793 | DWRC-1964 | T.turgidum | Landrace | 1.00E-04 | 0.002892 | 1.00E-04 | 1.00E-04 | 0.000165 | 0.415487 | 0.426805 | 0.154252 | 1.00E-04 | MED_LND |
| ITA_GDPv2-090 | DWRC-0284 | T.durum | Landrace | 9.99E-05 | 9.99E-05 | 9.99E-05 | 9.99E-05 | 0.523919 | 9.99E-05 | 0.475381 | 9.99E-05 | 9.99E-05 | MID_LND |
| IND_GDPv2-1000 | DWRC-1147 | T.durum | Landrace | 0.137271 | 1.00E-04 | 0.007844 | 0.148221 | 0.568436 | 0.128376 | 1.00E-04 | 0.009552 | 1.00E-04 | MID_LND |
| MAR_GDPv2-1001 | DWRC-1271 | T.durum | Landrace | 1.00E-04 | 1.00E-04 | 0.005875 | 0.078135 | 0.60154 | 0.052071 | 0.26198 | 1.00E-04 | 1.00E-04 | MID_LND |
| IRN_GDPv2-1051 | DWRC-2_042 | T.turanicum | Landrace | 9.99E-05 | 9.99E-05 | 9.99E-05 | 9.99E-05 | 0.978153 | 9.99E-05 | 9.99E-05 | 9.99E-05 | 0.021147 | MID_LND |
| ICARDA_GDPv2-258 | DWRC-0881 | T.durum | Landrace | 1.00E-04 | 1.00E-04 | 0.029983 | 0.003818 | 0.47756 | 0.351053 | 0.137186 | 1.00E-04 | 1.00E-04 | MID_LND |
| TUN_GDPv2-283 | DWRC-0991 | T.durum | Landrace | 1.00E-04 | 1.00E-04 | 1.00E-04 | 1.00E-04 | 0.366993 | 0.246193 | 0.274078 | 0.112237 | 1.00E-04 | MID_LND |
| ISR_GDPv2-288 | DWRC-1036 | T.durum | Landrace | 1.00E-04 | 1.00E-04 | 1.00E-04 | 0.025026 | 0.641752 | 0.227394 | 0.027631 | 0.077798 | 1.00E-04 | MID_LND |
| BGR_GDPv2-290 | DWRC-1039 | T.durum | Landrace | 1.00E-04 | 0.024184 | 0.034266 | 0.047389 | 0.391722 | 0.275328 | 0.076235 | 0.142759 | 0.008017 | MID_LND |
| IRN_GDPv2-330 | DWRC-1443 | T.durum | Landrace | 1.00E-04 | 0.003829 | 1.00E-04 | 1.00E-04 | 0.934317 | 0.056591 | 0.001224 | 1.00E-04 | 0.003639 | MID_LND |
| UKR_GDPv2-333 | DWRC-1505 | T.dicoccum | D_Emmer | 0.030092 | 0.076183 | 0.310944 | 1.00E-04 | 0.084736 | 0.398701 | 0.048698 | 1.00E-04 | 0.050446 | MID_LND |
| SWE_GDPv2-393 | DWRC-2204 | T.durum | Landrace | 0.011679 | 1.00E-04 | 1.00E-04 | 0.027907 | 0.387804 | 0.404992 | 0.140144 | 0.027175 | 1.00E-04 | MID_LND |
| MLT_GDPv2-396 | DWRC-2224 | T.durum | Landrace | 1.00E-04 | 1.00E-04 | 1.00E-04 | 1.00E-04 | 0.555576 | 0.292191 | 0.054422 | 0.068053 | 0.029359 | MID_LND |
| OMN_GDPv2-407 | DWRC-2284 | T.durum | Landrace | 0.078628 | 0.044875 | 0.016247 | 0.238104 | 0.3234 | 1.00E-04 | 0.136598 | 0.046634 | 0.115414 | MID_LND |
| EGY_GDPv2-548 | DWRC-0107 | T.durum | Landrace | 0.136989 | 1.00E-04 | 1.00E-04 | 0.145665 | 0.489687 | 0.131658 | 1.00E-04 | 0.092159 | 0.003541 | MID_LND |
| EGY_GDPv2-549 | DWRC-0110 | T.durum | Landrace | 0.064273 | 1.00E-04 | 1.00E-04 | 0.133695 | 0.57909 | 0.127451 | 0.031955 | 0.063237 | 1.00E-04 | MID_LND |
| EGY_GDPv2-550 | DWRC-0112 | T.durum | Landrace | 0.139953 | 1.00E-04 | 0.001057 | 0.13235 | 0.495717 | 0.137826 | 1.00E-04 | 0.082556 | 0.010341 | MID_LND |
| EGY_GDPv2-551 | DWRC-0113 | T.durum | Landrace | 0.010965 | 0.051399 | 0.114788 | 0.128056 | 0.252731 | 0.207468 | 0.164293 | 0.0702 | 1.00E-04 | MID_LND |
| TUR_GDPv2-559 | DWRC-1010 | T.durum | Landrace | 1.00E-04 | 0.020431 | 1.00E-04 | 0.021383 | 0.191153 | 0.412948 | 0.318327 | 0.035458 | 1.00E-04 | MID_LND |
| ISR_GDPv2-566 | DWRC-1034 | T.durum | Landrace | 0.012901 | 1.00E-04 | 1.00E-04 | 1.00E-04 | 0.613873 | 0.23803 | 0.072585 | 0.062211 | 1.00E-04 | MID_LND |
| ISR_GDPv2-567 | DWRC-1035 | T.durum | Landrace | 0.011205 | 1.00E-04 | 1.00E-04 | 0.020343 | 0.471093 | 0.285193 | 0.156495 | 0.055371 | 1.00E-04 | MID_LND |
| ITA_GDPv2-579 | DWRC-1149 | T.durum | Landrace | 9.99E-05 | 9.99E-05 | 9.99E-05 | 9.99E-05 | 0.521833 | 9.99E-05 | 0.477467 | 9.99E-05 | 9.99E-05 | MID_LND |
| SYR_GDPv2-580 | DWRC-1150 | T.durum | Landrace | 9.99E-05 | 9.99E-05 | 0.012032 | 9.99E-05 | 0.943513 | 9.99E-05 | 0.043856 | 9.99E-05 | 9.99E-05 | MID_LND |
| ETH_GDPv2-583 | DWRC-1155 | T.durum | Landrace | 1.00E-04 | 1.00E-04 | 0.003488 | 1.00E-04 | 0.904973 | 0.066851 | 1.00E-04 | 1.00E-04 | 0.024188 | MID_LND |
| AFG_GDPv2-584 | DWRC-1157 | T.durum | Landrace | 0.015369 | 1.00E-04 | 1.00E-04 | 0.060184 | 0.482671 | 0.244483 | 0.127577 | 0.069416 | 1.00E-04 | MID_LND |
| IRQ_GDPv2-585 | DWRC-1158 | T.durum | Landrace | 0.032409 | 0.043458 | 1.00E-04 | 1.00E-04 | 0.28693 | 0.266466 | 0.112771 | 0.257667 | 1.00E-04 | MID_LND |
| MAR_GDPv2-590 | DWRC-1164 | T.durum | Landrace | 1.00E-04 | 1.00E-04 | 1.00E-04 | 1.00E-04 | 0.368769 | 0.245583 | 0.273891 | 0.111257 | 1.00E-04 | MID_LND |
| OMN_GDPv2-591 | DWRC-1165 | T.durum | Landrace | 0.093219 | 0.053245 | 1.00E-04 | 0.237656 | 0.306035 | 0.003236 | 0.146013 | 0.020707 | 0.139789 | MID_LND |
| JOR_GDPv2-604 | DWRC-1190 | T.durum | Landrace | 9.99E-05 | 9.99E-05 | 0.027139 | 9.99E-05 | 0.972162 | 9.99E-05 | 9.99E-05 | 9.99E-05 | 9.99E-05 | MID_LND |
| YEM_GDPv2-609 | DWRC-1198 | T.durum | Landrace | 0.387218 | 0.048119 | 1.00E-04 | 0.046027 | 0.426841 | 0.032643 | 0.002182 | 0.035328 | 0.021541 | MID_LND |
| SYR_GDPv2-610 | DWRC-1199 | T.durum | Landrace | 0.004755 | 1.00E-04 | 0.019026 | 1.00E-04 | 0.92788 | 1.00E-04 | 0.04784 | 1.00E-04 | 1.00E-04 | MID_LND |
| IRN_GDPv2-611 | DWRC-1201 | T.durum | Landrace | 1.00E-04 | 1.00E-04 | 0.00764 | 1.00E-04 | 0.935927 | 0.040023 | 0.015911 | 1.00E-04 | 1.00E-04 | MID_LND |
| IRN_GDPv2-612 | DWRC-1202 | T.durum | Landrace | 9.99E-05 | 9.99E-05 | 9.99E-05 | 9.99E-05 | 0.94814 | 0.044375 | 0.006885 | 9.99E-05 | 9.99E-05 | MID_LND |
| RUS_GDPv2-614 | DWRC-1205 | T.durum | Landrace | 1.00E-04 | 1.00E-04 | 0.009516 | 1.00E-04 | 0.464055 | 0.432312 | 0.084839 | 0.008878 | 1.00E-04 | MID_LND |
| OMN_GDPv2-616 | DWRC-1208 | T.durum | Landrace | 0.072358 | 0.020456 | 1.00E-04 | 0.210537 | 0.310243 | 0.020849 | 0.156494 | 0.089175 | 0.119788 | MID_LND |
| ITA_GDPv2-627 | DWRC-1224 | T.durum | Landrace | 0.006958 | 0.000621 | 1.00E-04 | 1.00E-04 | 0.27839 | 0.40985 | 0.260543 | 0.043338 | 1.00E-04 | MID_LND |
| SYR_GDPv2-633 | DWRC-1231 | T.durum | Landrace | 9.99E-05 | 9.99E-05 | 0.000106 | 9.99E-05 | 0.99865 | 9.99E-05 | 0.000644 | 9.99E-05 | 9.99E-05 | MID_LND |
| SYR_GDPv2-634 | DWRC-1234 | T.durum | Landrace | 9.99E-05 | 9.99E-05 | 0.017429 | 9.99E-05 | 0.953174 | 9.99E-05 | 0.028798 | 9.99E-05 | 9.99E-05 | MID_LND |
| RUS_GDPv2-653 | DWRC-1262 | T.durum | Landrace | 0.003345 | 1.00E-04 | 0.018301 | 0.032658 | 0.405048 | 0.350161 | 0.173918 | 0.016369 | 1.00E-04 | MID_LND |
| USA_GDPv2-660 | DWRC-1277 | T.durum | Landrace | 1.00E-04 | 1.00E-04 | 1.00E-04 | 0.016108 | 0.852173 | 0.064865 | 0.066354 | 1.00E-04 | 1.00E-04 | MID_LND |
| IRQ_GDPv2-661 | DWRC-1281 | T.durum | Landrace | 0.039451 | 1.00E-04 | 1.00E-04 | 1.00E-04 | 0.419679 | 0.150635 | 0.319331 | 0.070504 | 1.00E-04 | MID_LND |
| IRQ_GDPv2-662 | DWRC-1282 | T.durum | Landrace | 0.006656 | 0.025429 | 1.00E-04 | 0.013089 | 0.438361 | 0.305468 | 0.12175 | 0.089047 | 1.00E-04 | MID_LND |
| IRQ_GDPv2-663 | DWRC-1283 | T.durum | Landrace | 0.019524 | 1.00E-04 | 1.00E-04 | 0.014188 | 0.852028 | 0.097673 | 0.016187 | 1.00E-04 | 1.00E-04 | MID_LND |
| IRQ_GDPv2-664 | DWRC-1284 | T.durum | Landrace | 1.00E-04 | 1.00E-04 | 1.00E-04 | 0.032504 | 0.594642 | 0.22175 | 0.147628 | 0.003077 | 1.00E-04 | MID_LND |
| IRQ_GDPv2-665 | DWRC-1285 | T.durum | Landrace | 1.00E-04 | 1.00E-04 | 0.006653 | 0.003806 | 0.914798 | 0.014716 | 0.059627 | 1.00E-04 | 1.00E-04 | MID_LND |
| PRT_GDPv2-670 | DWRC-1298 | T.durum | Landrace | 0.162117 | 1.00E-04 | 0.007812 | 0.072852 | 0.526418 | 0.176741 | 0.02627 | 0.027591 | 1.00E-04 | MID_LND |
| IND_GDPv2-671 | DWRC-1303 | T.durum | Landrace | 1.00E-04 | 1.00E-04 | 0.009132 | 1.00E-04 | 0.550665 | 0.014845 | 0.416079 | 0.008879 | 1.00E-04 | MID_LND |
| SAU_GDPv2-674 | DWRC-1306 | T.durum | Landrace | 0.074444 | 1.00E-04 | 1.00E-04 | 0.136157 | 0.577362 | 0.174796 | 1.00E-04 | 0.036841 | 1.00E-04 | MID_LND |
| EGY_GDPv2-675 | DWRC-1308 | T.durum | Landrace | 0.091063 | 0.007034 | 0.022454 | 0.032379 | 0.585464 | 0.09642 | 0.164982 | 1.00E-04 | 0.000105 | MID_LND |
| IND_GDPv2-676 | DWRC-1310 | T.durum | Landrace | 0.079393 | 1.00E-04 | 0.036119 | 0.040123 | 0.479111 | 0.306078 | 1.00E-04 | 0.057728 | 0.001248 | MID_LND |
| IND_GDPv2-677 | DWRC-1311 | T.durum | Landrace | 1.00E-04 | 1.00E-04 | 0.063798 | 0.005716 | 0.534731 | 0.021941 | 0.026801 | 0.346712 | 1.00E-04 | MID_LND |
| SYR_GDPv2-678 | DWRC-1312 | T.durum | Landrace | 1.00E-04 | 1.00E-04 | 1.00E-04 | 1.00E-04 | 0.750911 | 0.087145 | 0.154558 | 0.006886 | 1.00E-04 | MID_LND |
| SYR_GDPv2-679 | DWRC-1313 | T.durum | Landrace | 1.00E-04 | 1.00E-04 | 0.025369 | 0.000619 | 0.877182 | 0.023852 | 0.072579 | 1.00E-04 | 1.00E-04 | MID_LND |
| ETH_GDPv2-681 | DWRC-1315 | T.durum | Landrace | 0.043499 | 1.00E-04 | 1.00E-04 | 0.254711 | 0.41518 | 0.076905 | 0.209305 | 1.00E-04 | 1.00E-04 | MID_LND |
| IRN_GDPv2-687 | DWRC-1323 | T.durum | Landrace | 9.99E-05 | 0.003026 | 9.99E-05 | 9.99E-05 | 0.923621 | 0.072754 | 9.99E-05 | 9.99E-05 | 9.99E-05 | MID_LND |
| ITA_GDPv2-689 | DWRC-1325 | T.durum | Landrace | 0.010557 | 1.00E-04 | 0.002708 | 1.00E-04 | 0.357422 | 0.388364 | 0.227642 | 0.013008 | 1.00E-04 | MID_LND |
| IRN_GDPv2-690 | DWRC-1327 | T.durum | Landrace | 1.00E-04 | 0.016341 | 0.00732 | 0.026026 | 0.711463 | 0.15176 | 0.054825 | 0.030534 | 0.001631 | MID_LND |
| IRN_GDPv2-691 | DWRC-1328 | T.durum | Landrace | 0.006662 | 0.054781 | 0.005049 | 1.00E-04 | 0.898041 | 0.011859 | 0.023308 | 1.00E-04 | 1.00E-04 | MID_LND |
| TUN_GDPv2-693 | DWRC-1331 | T.durum | Landrace | 1.00E-04 | 0.006848 | 1.00E-04 | 1.00E-04 | 0.062064 | 0.387182 | 0.372988 | 0.170519 | 1.00E-04 | MID_LND |
| TUR_GDPv2-698 | DWRC-1337 | T.durum | Landrace | 0.128738 | 1.00E-04 | 0.003097 | 0.151157 | 0.590075 | 0.1043 | 1.00E-04 | 0.020001 | 0.002432 | MID_LND |
| SYR_GDPv2-699 | DWRC-1338 | T.durum | Landrace | 1.00E-04 | 1.00E-04 | 1.00E-04 | 0.045146 | 0.562357 | 0.194293 | 0.12665 | 0.071154 | 1.00E-04 | MID_LND |
| BIH_GDPv2-700 | DWRC-1339 | T.durum | Landrace | 0.001754 | 1.00E-04 | 0.029915 | 0.007522 | 0.899576 | 0.033525 | 0.008808 | 0.0187 | 1.00E-04 | MID_LND |
| IRN_GDPv2-702 | DWRC-1346 | T.durum | Landrace | 0.007295 | 0.009917 | 0.01918 | 0.020107 | 0.801313 | 0.075258 | 0.049868 | 0.016963 | 1.00E-04 | MID_LND |
| GRC_GDPv2-707 | DWRC-1360 | T.durum | Landrace | 0.013609 | 1.00E-04 | 1.00E-04 | 0.022626 | 0.386558 | 0.412397 | 0.137075 | 0.027435 | 1.00E-04 | MID_LND |
| GRC_GDPv2-710 | DWRC-1368 | T.durum | Landrace | 0.005619 | 1.00E-04 | 0.000976 | 1.00E-04 | 0.91575 | 0.011419 | 0.064841 | 1.00E-04 | 0.001096 | MID_LND |
| IRQ_GDPv2-711 | DWRC-1370 | T.durum | Landrace | 1.00E-04 | 1.00E-04 | 1.00E-04 | 0.010502 | 0.610481 | 0.061666 | 0.300395 | 0.016557 | 1.00E-04 | MID_LND |
| IRQ_GDPv2-712 | DWRC-1371 | T.durum | Landrace | 0.033759 | 0.033538 | 1.00E-04 | 0.030456 | 0.658091 | 0.025056 | 1.00E-04 | 0.134745 | 0.084155 | MID_LND |
| ITA_GDPv2-721 | DWRC-1393 | T.durum | Landrace | 1.00E-04 | 1.00E-04 | 0.001604 | 1.00E-04 | 0.284605 | 0.374209 | 0.251943 | 0.087239 | 1.00E-04 | MID_LND |
| ITA_GDPv2-725 | DWRC-1402 | T.durum | Landrace | 1.00E-04 | 1.00E-04 | 1.00E-04 | 1.00E-04 | 0.026551 | 0.342456 | 0.329824 | 0.30067 | 1.00E-04 | MID_LND |
| ITA_GDPv2-731 | DWRC-1409 | T.durum | Landrace | 0.018068 | 1.00E-04 | 1.00E-04 | 1.00E-04 | 0.060196 | 0.41677 | 0.329406 | 0.17516 | 1.00E-04 | MID_LND |
| ITA_GDPv2-733 | DWRC-1414 | T.durum | Landrace | 0.014787 | 1.00E-04 | 1.00E-04 | 1.00E-04 | 0.066606 | 0.41825 | 0.339725 | 0.160233 | 1.00E-04 | MID_LND |
| ITA_GDPv2-747 | DWRC-2043 | T.durum | Landrace | 1.00E-04 | 0.016725 | 0.021012 | 1.00E-04 | 0.850161 | 0.04422 | 0.067482 | 1.00E-04 | 1.00E-04 | MID_LND |
| MAR_GDPv2-748 | DWRC-2082 | T.durum | Landrace | 1.00E-04 | 1.00E-04 | 1.00E-04 | 1.00E-04 | 0.323363 | 0.267957 | 0.269367 | 0.138813 | 1.00E-04 | MID_LND |
| ITA_GDPv2-750 | DWRC-2121 | T.durum | Landrace | 0.029264 | 1.00E-04 | 0.00089 | 1.00E-04 | 0.410762 | 0.329901 | 0.227966 | 1.00E-04 | 0.000917 | MID_LND |
| TUN_GDPv2-753 | DWRC-2171 | T.durum | Landrace | 1.00E-04 | 1.00E-04 | 1.00E-04 | 1.00E-04 | 0.338589 | 0.335135 | 0.256474 | 0.069303 | 1.00E-04 | MID_LND |
| TUR_GDPv2-754 | DWRC-2184 | T.durum | Landrace | 1.00E-04 | 1.00E-04 | 0.022544 | 1.00E-04 | 0.928139 | 0.020383 | 0.028435 | 1.00E-04 | 1.00E-04 | MID_LND |
| UK_GDPv2-758 | DWRC-2243 | T.durum | Landrace | 0.01793 | 1.00E-04 | 1.00E-04 | 1.00E-04 | 0.326924 | 0.251502 | 0.313258 | 0.089986 | 1.00E-04 | MID_LND |
| PAK_GDPv2-760 | DWRC-2251 | T.durum | Landrace | 0.019664 | 1.00E-04 | 1.00E-04 | 0.024272 | 0.507585 | 0.20999 | 0.124514 | 0.103214 | 0.010563 | MID_LND |
| IRQ_GDPv2-761 | DWRC-2261 | T.durum | Landrace | 1.00E-04 | 1.00E-04 | 1.00E-04 | 0.013799 | 0.813801 | 0.093906 | 0.077995 | 1.00E-04 | 1.00E-04 | MID_LND |
| IRN_GDPv2-763 | DWRC-2288 | T.turanicum | Landrace | 9.99E-05 | 9.99E-05 | 9.99E-05 | 9.99E-05 | 0.914656 | 0.084645 | 9.99E-05 | 9.99E-05 | 9.99E-05 | MID_LND |
| AZE_GDPv2-765 | DWRC-1921 | T.turanicum | Landrace | 1.00E-04 | 0.009608 | 1.00E-04 | 1.00E-04 | 0.54903 | 0.315497 | 0.03765 | 0.087816 | 1.00E-04 | MID_LND |
| IRQ_GDPv2-766 | DWRC-1922 | T.turanicum | Landrace | 9.99E-05 | 9.99E-05 | 9.99E-05 | 9.99E-05 | 0.98051 | 9.99E-05 | 9.99E-05 | 9.99E-05 | 0.018791 | MID_LND |
| IRN_GDPv2-768 | DWRC-1926 | T.turanicum | Landrace | 9.99E-05 | 9.99E-05 | 9.99E-05 | 9.99E-05 | 0.979304 | 9.99E-05 | 9.99E-05 | 9.99E-05 | 0.019996 | MID_LND |
| HUN_GDPv2-769 | DWRC-1928 | T.turanicum | Landrace | 9.99E-05 | 9.99E-05 | 9.99E-05 | 9.99E-05 | 0.977354 | 9.99E-05 | 9.99E-05 | 9.99E-05 | 0.021947 | MID_LND |
| TUR_GDPv2-771 | DWRC-1930 | T.turanicum | Landrace | 1.00E-04 | 0.002561 | 1.00E-04 | 1.00E-04 | 0.883788 | 0.02864 | 1.00E-04 | 0.067541 | 0.017071 | MID_LND |
| IRN_GDPv2-772 | DWRC-1932 | T.turanicum | Landrace | 9.99E-05 | 9.99E-05 | 9.99E-05 | 9.99E-05 | 0.977161 | 9.99E-05 | 9.99E-05 | 9.99E-05 | 0.022139 | MID_LND |
| AFG_GDPv2-773 | DWRC-1933 | T.turanicum | Landrace | 9.99E-05 | 9.99E-05 | 9.99E-05 | 9.99E-05 | 0.974933 | 9.99E-05 | 9.99E-05 | 9.99E-05 | 0.024368 | MID_LND |
| AUS_GDPv2-774 | DWRC-1934 | T.turanicum | Landrace | 0.032039 | 1.00E-04 | 1.00E-04 | 1.00E-04 | 0.496551 | 0.044384 | 0.31083 | 0.107342 | 0.008555 | MID_LND |
| IRN_GDPv2-776 | DWRC-1939 | T.polonicum | Landrace | 1.00E-04 | 1.00E-04 | 1.00E-04 | 0.002826 | 0.349813 | 0.318858 | 0.091483 | 0.23662 | 1.00E-04 | MID_LND |
| UK_GDPv2-778 | DWRC-1941 | T.polonicum | Landrace | 1.00E-04 | 1.00E-04 | 1.00E-04 | 1.00E-04 | 0.358689 | 0.315269 | 0.093434 | 0.232108 | 1.00E-04 | MID_LND |
| IRQ_GDPv2-801 | DWRC-1978 | T.turanicum | Landrace | 9.99E-05 | 9.99E-05 | 9.99E-05 | 9.99E-05 | 0.997046 | 9.99E-05 | 9.99E-05 | 9.99E-05 | 0.002255 | MID_LND |
| IRN_GDPv2-803 | DWRC-1980 | T.carthlicum | Landrace | 1.00E-04 | 1.00E-04 | 0.01531 | 0.010045 | 0.438854 | 0.418764 | 0.116627 | 1.00E-04 | 1.00E-04 | MID_LND |
| SYR_GDPv2-859 | DWRC-2017 | T.dicoccoides | W_Emmer | 1.00E-04 | 0.030208 | 1.00E-04 | 0.006185 | 1.00E-04 | 0.328235 | 0.318715 | 0.013844 | 0.302513 | MID_LND |
| IRN_GDPv2-895 | DWRC-1572 | T.dicoccum | D_Emmer | 0.020488 | 0.291385 | 0.130504 | 1.00E-04 | 1.00E-04 | 0.410231 | 0.006396 | 0.097472 | 0.043324 | MID_LND |
| KEN_GDPv2-883 | DWRC-1541 | T.dicoccum | D_Emmer | 0.061402 | 0.375368 | 0.470462 | 0.000774 | 0.037004 | 1.00E-04 | 0.005108 | 0.043239 | 0.006543 | MIDEEU_DOM |
| RUS_GDPv2-884 | DWRC-1543 | T.dicoccum | D_Emmer | 0.042436 | 0.238678 | 0.658105 | 1.00E-04 | 1.00E-04 | 0.039596 | 1.00E-04 | 0.020261 | 0.000625 | MIDEEU_DOM |
| RUS_GDPv2-885 | DWRC-1544 | T.dicoccum | D_Emmer | 0.027106 | 0.08892 | 0.797232 | 1.00E-04 | 0.00391 | 1.00E-04 | 1.00E-04 | 0.022707 | 0.059826 | MIDEEU_DOM |
| UKR_GDPv2-889 | DWRC-1560 | T.dicoccum | D_Emmer | 9.99E-05 | 9.99E-05 | 0.999201 | 9.99E-05 | 9.99E-05 | 9.99E-05 | 9.99E-05 | 9.99E-05 | 9.99E-05 | MIDEEU_DOM |
| ARM_GDPv2-890 | DWRC-1564 | T.dicoccum | D_Emmer | 1.00E-04 | 0.095455 | 0.88892 | 1.00E-04 | 1.00E-04 | 1.00E-04 | 0.00538 | 0.009745 | 1.00E-04 | MIDEEU_DOM |
| IRN_GDPv2-891 | DWRC-1565 | T.dicoccum | D_Emmer | 1.00E-04 | 0.404339 | 0.502918 | 0.005876 | 0.022235 | 0.004511 | 1.00E-04 | 0.059821 | 1.00E-04 | MIDEEU_DOM |
| IRN_GDPv2-892 | DWRC-1566 | T.dicoccum | D_Emmer | 0.016124 | 0.194561 | 0.50713 | 0.017377 | 1.00E-04 | 0.002492 | 1.00E-04 | 0.262016 | 1.00E-04 | MIDEEU_DOM |
| IRN_GDPv2-896 | DWRC-1573 | T.dicoccum | D_Emmer | 9.99E-05 | 9.99E-05 | 0.999201 | 9.99E-05 | 9.99E-05 | 9.99E-05 | 9.99E-05 | 9.99E-05 | 9.99E-05 | MIDEEU_DOM |
| RUS_GDPv2-900 | DWRC-1582 | T.dicoccum | D_Emmer | 1.00E-04 | 0.009925 | 0.972523 | 1.00E-04 | 0.01483 | 0.002222 | 1.00E-04 | 1.00E-04 | 1.00E-04 | MIDEEU_DOM |
| ARM_GDPv2-902 | DWRC-1584 | T.dicoccum | D_Emmer | 9.99E-05 | 0.017079 | 0.982222 | 9.99E-05 | 9.99E-05 | 9.99E-05 | 9.99E-05 | 9.99E-05 | 9.99E-05 | MIDEEU_DOM |
| GEO_GDPv2-903 | DWRC-1586 | T.dicoccum | D_Emmer | 9.99E-05 | 9.99E-05 | 0.999201 | 9.99E-05 | 9.99E-05 | 9.99E-05 | 9.99E-05 | 9.99E-05 | 9.99E-05 | MIDEEU_DOM |
| ESP_GDPv2-905 | DWRC-1592 | T.dicoccum | D_Emmer | 9.99E-05 | 9.99E-05 | 0.999201 | 9.99E-05 | 9.99E-05 | 9.99E-05 | 9.99E-05 | 9.99E-05 | 9.99E-05 | MIDEEU_DOM |
| YUG_GDPv2-920 | DWRC-1625 | T.dicoccum | D_Emmer | 0.050476 | 0.275904 | 0.569989 | 1.00E-04 | 0.016353 | 1.00E-04 | 0.00559 | 1.00E-04 | 0.081389 | MIDEEU_DOM |
| IRN_GDPv2-925 | DWRC-1648 | T.dicoccum | D_Emmer | 9.99E-05 | 9.99E-05 | 0.999201 | 9.99E-05 | 9.99E-05 | 9.99E-05 | 9.99E-05 | 9.99E-05 | 9.99E-05 | MIDEEU_DOM |
| IRN_GDPv2-926 | DWRC-1653 | T.dicoccum | D_Emmer | 0.043536 | 0.260963 | 0.670296 | 0.009164 | 0.015642 | 1.00E-04 | 1.00E-04 | 1.00E-04 | 1.00E-04 | MIDEEU_DOM |
| IRN_GDPv2-927 | DWRC-1657 | T.dicoccum | D_Emmer | 1.00E-04 | 1.00E-04 | 0.955936 | 0.005726 | 1.00E-04 | 0.013359 | 0.024479 | 1.00E-04 | 1.00E-04 | MIDEEU_DOM |
| IRN_GDPv2-928 | DWRC-1659 | T.dicoccum | D_Emmer | 9.99E-05 | 9.99E-05 | 0.982685 | 9.99E-05 | 9.99E-05 | 9.99E-05 | 9.99E-05 | 9.99E-05 | 0.016616 | MIDEEU_DOM |
| ROU_GDPv2-942 | DWRC-1693 | T.dicoccum | D_Emmer | 9.99E-05 | 9.99E-05 | 0.999201 | 9.99E-05 | 9.99E-05 | 9.99E-05 | 9.99E-05 | 9.99E-05 | 9.99E-05 | MIDEEU_DOM |
| CHN_GDPv2-948 | DWRC-1706 | T.dicoccum | D_Emmer | 1.00E-04 | 0.351935 | 0.626319 | 0.019635 | 1.00E-04 | 0.001611 | 1.00E-04 | 1.00E-04 | 1.00E-04 | MIDEEU_DOM |
| SYR_GDPv2-952 | DWRC-1711 | T.dicoccum | D_Emmer | 0.076074 | 0.131406 | 0.611228 | 0.015639 | 0.023655 | 1.00E-04 | 1.00E-04 | 0.022126 | 0.119673 | MIDEEU_DOM |
| YEM_GDPv2-954 | DWRC-1715 | T.dicoccum | D_Emmer | 0.029814 | 0.273376 | 0.478365 | 1.00E-04 | 0.086942 | 0.087295 | 1.00E-04 | 0.038684 | 0.005324 | MIDEEU_DOM |
| na_GDPv2-959 | DWRC-1990 | T.dicoccum | D_Emmer | 9.99E-05 | 9.99E-05 | 0.999201 | 9.99E-05 | 9.99E-05 | 9.99E-05 | 9.99E-05 | 9.99E-05 | 9.99E-05 | MIDEEU_DOM |
| na_GDPv2-975 | DWRC-2140 | T.dicoccum | D_Emmer | 1.00E-04 | 0.025601 | 0.373332 | 0.007204 | 0.208426 | 0.215351 | 0.142094 | 1.00E-04 | 0.027792 | MIDEEU_DOM |
| ISR_GDPv2-1002 | DWRC-1465 | T.dicoccoides | W_Emmer | 1.00E-04 | 1.00E-04 | 0.084593 | 0.020482 | 0.117376 | 0.075843 | 0.061635 | 0.024214 | 0.615657 | WLD_EM |
| ISR_GDPv2-1003 | DWRC-1469 | T.dicoccoides | W_Emmer | 0.007484 | 9.99E-05 | 9.99E-05 | 0.001145 | 9.99E-05 | 9.99E-05 | 9.99E-05 | 9.99E-05 | 0.990772 | WLD_EM |
| SYR_GDPv2-1004 | DWRC-1475 | T.dicoccoides | W_Emmer | 1.00E-04 | 1.00E-04 | 0.00701 | 0.032314 | 1.00E-04 | 1.00E-04 | 1.00E-04 | 0.002175 | 0.958001 | WLD_EM |
| LBN_GDPv2-1005 | DWRC-1482 | T.dicoccoides | W_Emmer | 0.018331 | 9.99E-05 | 0.005857 | 9.99E-05 | 9.99E-05 | 9.99E-05 | 9.99E-05 | 9.99E-05 | 0.975213 | WLD_EM |
| JOR_GDPv2-1008 | DWRC-2486 | T.dicoccoides | W_Emmer | 9.99E-05 | 9.99E-05 | 9.99E-05 | 9.99E-05 | 9.99E-05 | 9.99E-05 | 9.99E-05 | 9.99E-05 | 0.999201 | WLD_EM |
| JOR_GDPv2-1009 | DWRC-2490 | T.dicoccoides | W_Emmer | 9.99E-05 | 9.99E-05 | 9.99E-05 | 9.99E-05 | 9.99E-05 | 9.99E-05 | 9.99E-05 | 9.99E-05 | 0.999201 | WLD_EM |
| AFG_GDPv2-587 | DWRC-1160 | T.durum | Landrace | 1.00E-04 | 1.00E-04 | 0.151282 | 0.418827 | 0.021739 | 1.00E-04 | 1.00E-04 | 0.109137 | 0.298615 | WLD_EM |
| TUR_GDPv2-820 | DWRC-1446 | T.dicoccoides | W_Emmer | 0.018593 | 0.096845 | 0.221953 | 0.138612 | 0.023415 | 1.00E-04 | 1.00E-04 | 0.009419 | 0.490961 | WLD_EM |
| ISR_GDPv2-821 | DWRC-1447 | T.dicoccoides | W_Emmer | 0.000889 | 9.99E-05 | 9.99E-05 | 9.99E-05 | 9.99E-05 | 9.99E-05 | 9.99E-05 | 9.99E-05 | 0.998411 | WLD_EM |
| ISR_GDPv2-823 | DWRC-1449 | T.dicoccoides | W_Emmer | 0.024577 | 0.086572 | 0.166942 | 0.004051 | 1.00E-04 | 1.00E-04 | 1.00E-04 | 0.114677 | 0.602882 | WLD_EM |
| TUR_GDPv2-824 | DWRC-1451 | T.dicoccoides | W_Emmer | 0.037882 | 0.294738 | 0.200152 | 0.009464 | 0.016312 | 0.031717 | 0.017727 | 0.061418 | 0.33059 | WLD_EM |
| LBN_GDPv2-825 | DWRC-1452 | T.dicoccoides | W_Emmer | 1.00E-04 | 1.00E-04 | 0.304386 | 1.00E-04 | 1.00E-04 | 0.189002 | 0.153135 | 0.001188 | 0.351889 | WLD_EM |
| ISR_GDPv2-826 | DWRC-1453 | T.dicoccoides | W_Emmer | 0.001733 | 1.00E-04 | 1.00E-04 | 0.149891 | 0.170564 | 0.230927 | 0.235144 | 0.007988 | 0.203553 | WLD_EM |
| LBN_GDPv2-828 | DWRC-1456 | T.dicoccoides | W_Emmer | 0.027722 | 9.99E-05 | 9.99E-05 | 9.99E-05 | 9.99E-05 | 9.99E-05 | 0.008153 | 9.99E-05 | 0.963526 | WLD_EM |
| TUR_GDPv2-829 | DWRC-1457 | T.dicoccoides | W_Emmer | 0.04918 | 0.032157 | 0.085407 | 0.002039 | 0.006809 | 1.00E-04 | 1.00E-04 | 1.00E-04 | 0.824109 | WLD_EM |
| TUR_GDPv2-831 | DWRC-1459 | T.dicoccoides | W_Emmer | 0.055607 | 0.031906 | 0.005912 | 0.027237 | 0.005665 | 0.026046 | 0.03538 | 0.021632 | 0.790614 | WLD_EM |
| na_GDPv2-833 | DWRC-1463 | T.dicoccoides | W_Emmer | 1.00E-04 | 1.00E-04 | 0.013951 | 0.009323 | 0.067018 | 0.349805 | 0.10893 | 0.074113 | 0.37666 | WLD_EM |
| ISR_GDPv2-834 | DWRC-1466 | T.dicoccoides | W_Emmer | 0.019815 | 9.99E-05 | 9.99E-05 | 0.014541 | 9.99E-05 | 9.99E-05 | 9.99E-05 | 9.99E-05 | 0.965045 | WLD_EM |
| LBN_GDPv2-835 | DWRC-1467 | T.dicoccoides | W_Emmer | 1.00E-04 | 0.017802 | 0.088812 | 0.037759 | 0.478075 | 0.09063 | 0.109742 | 1.00E-04 | 0.17698 | WLD_EM |
| ISR_GDPv2-836 | DWRC-1470 | T.dicoccoides | W_Emmer | 1.00E-04 | 0.002317 | 0.215661 | 0.01946 | 0.112476 | 0.139922 | 0.207146 | 1.00E-04 | 0.302818 | WLD_EM |
| LBN_GDPv2-837 | DWRC-1472 | T.dicoccoides | W_Emmer | 1.00E-04 | 0.002566 | 0.183183 | 0.031047 | 0.124438 | 0.145013 | 0.217608 | 1.00E-04 | 0.295945 | WLD_EM |
| ISR_GDPv2-838 | DWRC-1474 | T.dicoccoides | W_Emmer | 9.99E-05 | 9.99E-05 | 9.99E-05 | 9.99E-05 | 9.99E-05 | 9.99E-05 | 9.99E-05 | 9.99E-05 | 0.999201 | WLD_EM |
| SYR_GDPv2-839 | DWRC-1477 | T.dicoccoides | W_Emmer | 0.057918 | 0.166219 | 0.204194 | 0.037278 | 0.009678 | 0.116605 | 0.029606 | 0.151187 | 0.227314 | WLD_EM |
| ISR_GDPv2-840 | DWRC-1479 | T.dicoccoides | W_Emmer | 1.00E-04 | 0.140996 | 0.197839 | 0.024056 | 0.085357 | 0.054618 | 0.141789 | 0.106559 | 0.248686 | WLD_EM |
| LBN_GDPv2-841 | DWRC-1481 | T.dicoccoides | W_Emmer | 0.012307 | 1.00E-04 | 1.00E-04 | 1.00E-04 | 1.00E-04 | 0.06958 | 0.393406 | 0.093068 | 0.431239 | WLD_EM |
| LBN_GDPv2-844 | DWRC-1488 | T.dicoccoides | W_Emmer | 1.00E-04 | 1.00E-04 | 0.256005 | 0.00604 | 1.00E-04 | 0.173307 | 0.132864 | 1.00E-04 | 0.431385 | WLD_EM |
| na_GDPv2-845 | DWRC-1492 | T.dicoccoides | W_Emmer | 0.01005 | 0.026843 | 0.060972 | 0.020718 | 1.00E-04 | 0.003463 | 1.00E-04 | 1.00E-04 | 0.877654 | WLD_EM |
| na_GDPv2-846 | DWRC-1493 | T.dicoccoides | W_Emmer | 0.010865 | 0.007593 | 1.00E-04 | 1.00E-04 | 0.001634 | 0.046629 | 0.039144 | 0.007136 | 0.8868 | WLD_EM |
| ISR_GDPv2-850 | DWRC-1498 | T.dicoccoides | W_Emmer | 0.025551 | 9.99E-05 | 0.011794 | 9.99E-05 | 9.99E-05 | 9.99E-05 | 9.99E-05 | 9.99E-05 | 0.962055 | WLD_EM |
| HUN_GDPv2-854 | DWRC-2012 | T.dicoccoides | W_Emmer | 0.017076 | 0.106294 | 0.186605 | 0.011453 | 1.00E-04 | 1.00E-04 | 1.00E-04 | 0.111333 | 0.566939 | WLD_EM |
| ISR_GDPv2-855 | DWRC-2013 | T.dicoccoides | W_Emmer | 1.00E-04 | 0.037318 | 0.030485 | 0.071137 | 1.00E-04 | 0.344046 | 0.229712 | 1.00E-04 | 0.287002 | WLD_EM |
| na_GDPv2-856 | DWRC-2014 | T.dicoccoides | W_Emmer | 0.080338 | 0.075325 | 0.224172 | 1.00E-04 | 1.00E-04 | 1.00E-04 | 1.00E-04 | 0.454752 | 0.165013 | WLD_EM |
| SYR_GDPv2-858 | DWRC-2016 | T.dicoccoides | W_Emmer | 0.057973 | 1.00E-04 | 0.015602 | 1.00E-04 | 0.045032 | 0.337659 | 0.001561 | 1.00E-04 | 0.541874 | WLD_EM |
| LBN_GDPv2-860 | DWRC-2146 | T.dicoccoides | W_Emmer | 1.00E-04 | 0.040166 | 0.04209 | 1.00E-04 | 1.00E-04 | 0.290432 | 0.288857 | 0.028096 | 0.310059 | WLD_EM |
| TUR_GDPv2-861 | DWRC-2147 | T.dicoccoides | W_Emmer | 1.00E-04 | 0.085541 | 0.210991 | 0.22752 | 0.015249 | 1.00E-04 | 1.00E-04 | 0.060137 | 0.400264 | WLD_EM |
| ISR_GDPv2-862 | DWRC-2149 | T.dicoccoides | W_Emmer | 0.040546 | 1.00E-04 | 0.043341 | 0.097273 | 1.00E-04 | 0.105799 | 0.146247 | 1.00E-04 | 0.566494 | WLD_EM |
| SYR_GDPv2-863 | DWRC-2484 | T.dicoccoides | W_Emmer | 0.022898 | 1.00E-04 | 1.00E-04 | 0.011726 | 1.00E-04 | 0.166709 | 0.246442 | 1.00E-04 | 0.551825 | WLD_EM |
| SYR_GDPv2-865 | DWRC-2493 | T.dicoccoides | W_Emmer | 0.027233 | 1.00E-04 | 0.028008 | 0.002786 | 0.021552 | 0.03878 | 0.003291 | 0.00868 | 0.86957 | WLD_EM |
| JOR_GDPv2-866 | DWRC-2494 | T.dicoccoides | W_Emmer | 0.018611 | 9.99E-05 | 9.99E-05 | 9.99E-05 | 9.99E-05 | 0.007984 | 9.99E-05 | 9.99E-05 | 0.972805 | WLD_EM |
| LBN_GDPv2-867 | DWRC-2495 | T.dicoccoides | W_Emmer | 0.019142 | 9.99E-05 | 9.99E-05 | 9.99E-05 | 9.99E-05 | 9.99E-05 | 9.99E-05 | 9.99E-05 | 0.980159 | WLD_EM |
| JOR_GDPv2-868 | DWRC-2496 | T.dicoccoides | W_Emmer | 0.04391 | 1.00E-04 | 0.021234 | 0.040629 | 1.00E-04 | 1.00E-04 | 1.00E-04 | 1.00E-04 | 0.893728 | WLD_EM |
| JOR_GDPv2-870 | DWRC-2499 | T.dicoccoides | W_Emmer | 0.031628 | 0.08057 | 0.264092 | 0.093735 | 0.01323 | 1.00E-04 | 1.00E-04 | 1.00E-04 | 0.516445 | WLD_EM |
| TUR_GDPv2-871 | DWRC-2500 | T.dicoccoides | W_Emmer | 1.00E-04 | 0.082579 | 0.290094 | 0.299441 | 1.00E-04 | 1.00E-04 | 0.018241 | 0.011381 | 0.297963 | WLD_EM |
| TUR_GDPv2-872 | DWRC-2501 | T.dicoccoides | W_Emmer | 0.028133 | 1.00E-04 | 0.026703 | 1.00E-04 | 1.00E-04 | 1.00E-04 | 0.001535 | 1.00E-04 | 0.943129 | WLD_EM |
| ETH_GDPv2-971 | DWRC-2005 | T.dicoccum | D_Emmer | 0.215546 | 0.131108 | 1.00E-04 | 1.00E-04 | 0.226089 | 0.06399 | 0.052133 | 0.033223 | 0.277711 | WLD_EM |
| na_GDPv2-973 | DWRC-2008 | T.dicoccum | D_Emmer | 0.084705 | 0.155793 | 0.213214 | 0.058141 | 1.00E-04 | 0.086423 | 0.006829 | 0.158722 | 0.236072 | WLD_EM |

Accessions ^1^the first three letters indicate the name of the country of origin based on United Nations three-letters country code, followed by accession name in Global Durum Diversity Panel. Each color indicates the subpopulation based on the ancestral coefficient of the individuals at K=9.

**
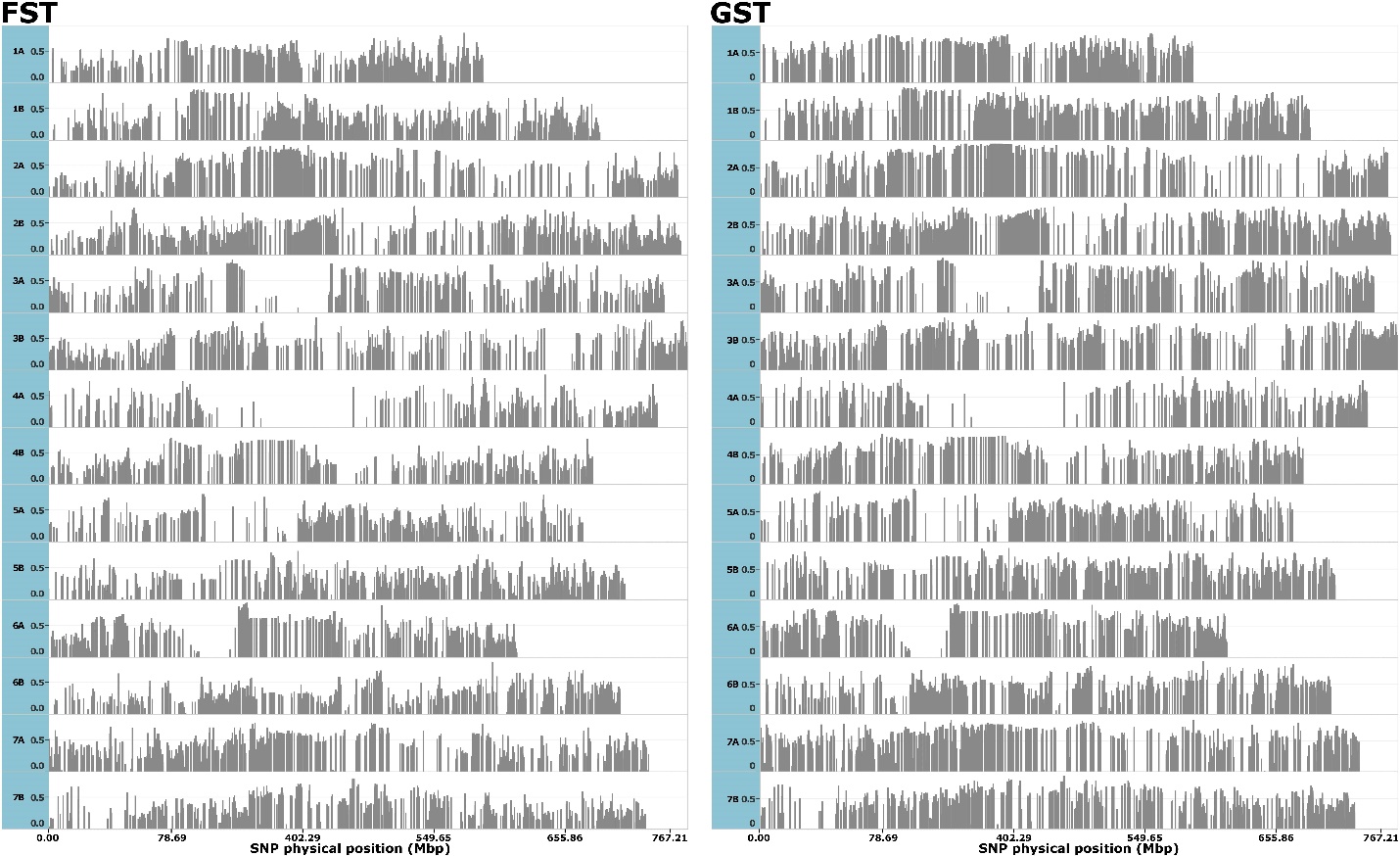
**

**Figure S1:** Pattern of differentiation at each locus based on *F_ST_* and *_GST_*

**
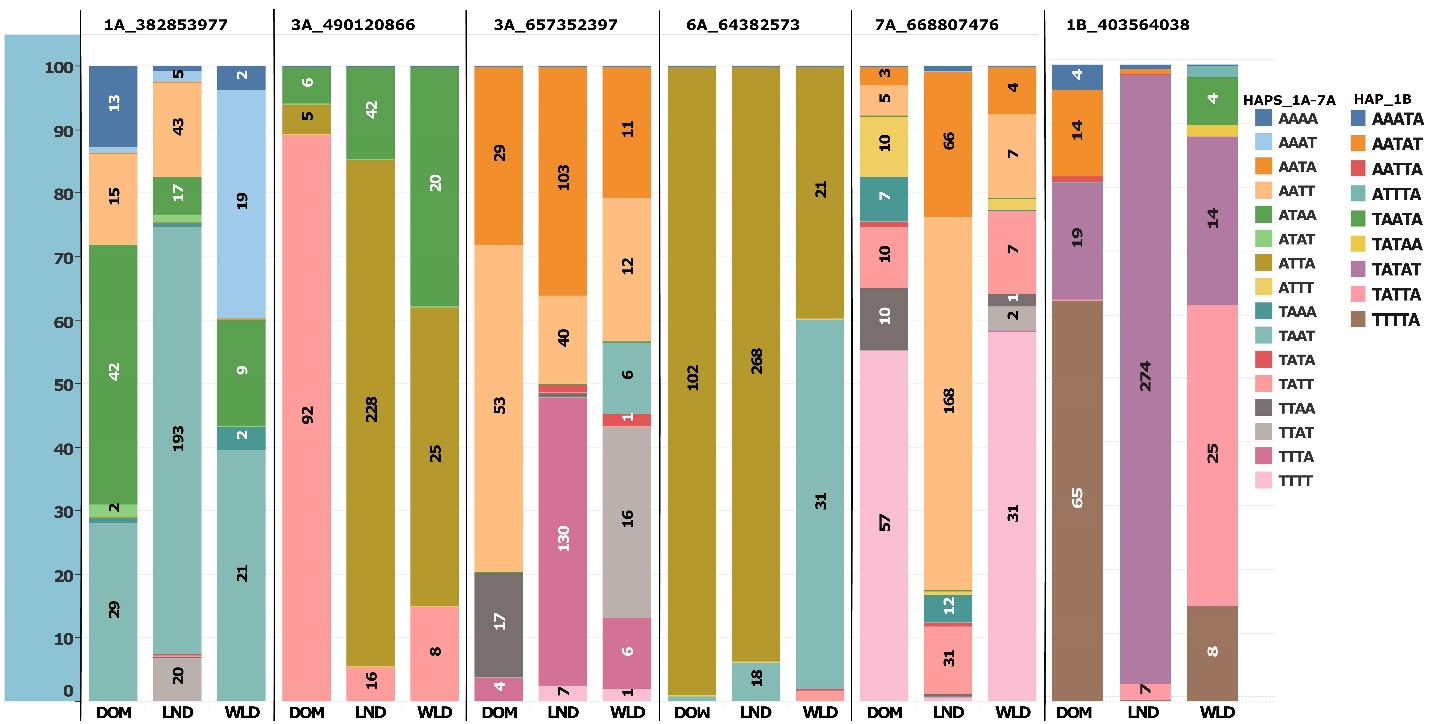
**

**Figure S2.** Allelic frequencies at each locus in landrace, domesticated and wild emmer wheats. The Y-axis and the labels show the percent and absolute frequencies, respectively.

**
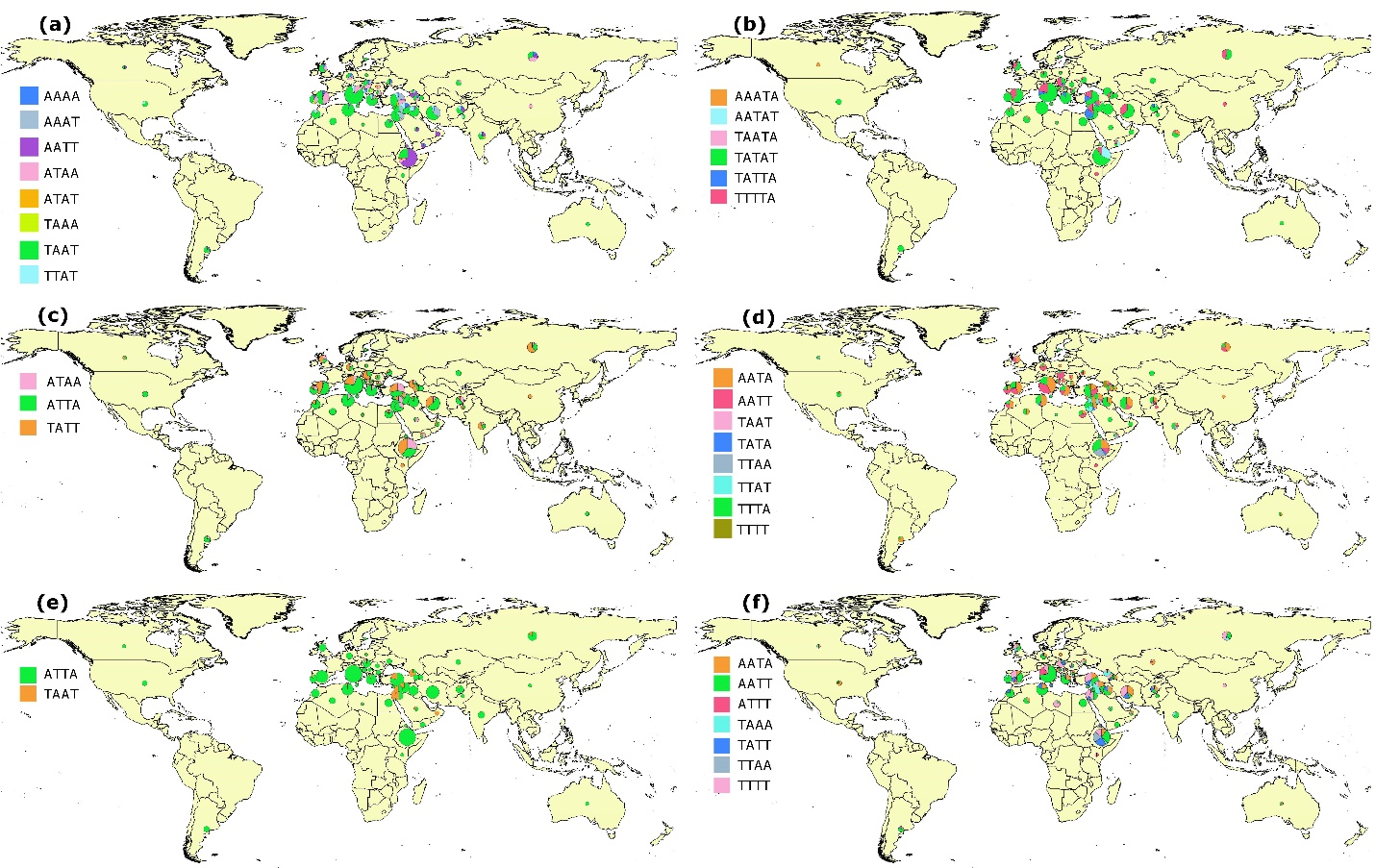
**

**Figure S3**. The geographic distribution of single-locus haplotypes. (**a**) Haplotypes at locus 1A, (**b**) haplotype 1B (**c**) haplotype 3A1, (**d**) haplotype 3A2, (**e**) haplotype 6A and (**f**) haplotype 7A. Each color in each pie chart represents a haplotype, the size of the pie circle is proportional with the number of accessions included.

**Table S2** Major genes in the uniquely differentiated region of chromosome 2B.

| ACCESSION | GENE_NAME | CHRO | START | Trait regulated |
| --- | --- | --- | --- | --- |
| TRAESCS2B02G273500 | PIF4 | 2B | 374801457 | FL |
| TRAESCS2B02G254000 | CBT | 2B | 281872239 | FL, Cr |
| TRAESCS2B02G267000 | MTERF2 | 2B | 359612800 | Cr |
| TRAESCS2B02G271700 | CIPK23 | 2B | 373301321 | FL, Sdr |
| TRAESCS2B02G250900 | SPL3 | 2B | 260765427 | FL, Sdr,Vr |
| TRAESCS2B02G248700 | CPK9 | 2B | 255050988 | FL,Sdr |
| TRAESCS2B02G266700 | BAM1 | 2B | 359310845 | FL |
| TRAESCS2B02G264000 | TIFY5 | 2B | 356582219 | FL |
| TRAESCS2B02G256600 | GRF9 | 2B | 298324714 | FL |
| TRAESCS2B02G253600 | CSN7 | 2B | 278435469 | FL,Sdr |
| TRAESCS2B02G264100 | GLO1 | 2B | 356594198 | Cr |
| TRAESCS2B02G268900 | OEP61 | 2B | 364316379 | FL |
| TRAESCS2B02G263600 | HEN1 | 2B | 354875272 | FL |
| TRAESCS2B02G268100 | CRL5 | 2B | 361422652 | FL |
| TRAESCS2B02G270300 | PSBO1 | 2B | 368613668 | Cr |
| TRAESCS2B02G267500 | PSBP | 2B | 360481412 | Cr |
| TRAESCS2B02G273900 | ATPC1 | 2B | 375544513 | Cr |
| TRAESCS2B02G251400 | pod | 2B | 263811436 | FL |
| TRAESCS2B02G260300 | TRAESCS2B02G260300 | 2B | 326675374 | Sdr |
| TRAESCS2B02G250500 | CAR2 | 2B | 260605521 | Sdr |
| TRAESCS2B02G263000 | EXO84A | 2B | 350298617 | FL |
| TRAESCS2B02G248900 | TRAESCS2B02G248900 | 2B | 256326294 | Cr |
| TRAESCS2B02G249200 | PBF | 2B | 257522750 | FL |
| TRAESCS2B02G255300 | HRD1 | 2B | 291583780 | FL |
| TRAESCS2B02G257000 | SHL | 2B | 302039648 | Cr |
| TRAESCS2B02G260100 | HD16 | 2B | 325844444 | FL,Cr,Sdr |
| TRAESCS2B02G271300 | CP33 | 2B | 371165255 | Cr |
| TRAESCS2B02G268400 | NCED2 | 2B | 363341361 | Sdr |
| TRAESCS2B02G250000 | TPX2 | 2B | 259029791 | FL |
| TRAESCS2B02G246600 | LTD | 2B | 250743348 | Cr |
| TRAESCS2B02G257300 | PNP1 | 2B | 306885521 | Cr |
| TRAESCS2B02G257800 | PKP1 | 2B | 310928737 | Cr |
| TRAESCS2B02G270600 | AAO1 | 2B | 369476063 | Sdr |
| TRAESCS2B02G270500 | OVA6 | 2B | 368972606 | Cr |
| TRAESCS2B02G269700 | Rf1 | 2B | 367657356 | Cr |
| TRAESCS2B02G271900 | ERD4 | 2B | 373476491 | Sdr |
| TRAESCS2B02G260200 | ZKT | 2B | 326271354 | Cr |
| TRAESCS2B02G263300 | EMB2247 | 2B | 353564383 | Cr |
| TRAESCS2B02G268000 | PR | 2B | 361284425 | Cr |
| TRAESCS2B02G269300 | DET2 | 2B | 364947818 | Cr |
| TRAESCS2B02G275000 | MAP1B | 2B | 379094707 | Cr |
| TRAESCS2B02G255900 | NS1 | 2B | 296012982 | Cr |
| TRAESCS2B02G272300 | PSAK | 2B | 373746850 | Cr |
| TRAESCS2B02G267900 | PR | 2B | 361125893 | Cr |
| TRAESCS2B02G266900 | PSKR | 2B | 359608269 | Sdr |
| TRAESCS2B02G247300 | HSP15.4 | 2B | 253713658 | Cr |
| TRAESCS2B02G275200 | CF9 | 2B | 379832241 | Cr |
| TRAESCS2B02G255000 | CYP21-3 | 2B | 288470184 | Sdr |
| TRAESCS2B02G253300 | TRAESCS2B02G253300 | 2B | 275294308 | Cr |
| TRAESCS2B02G257100 | TRAESCS2B02G257100 | 2B | 303094016 | Cr |
| TRAESCS2B02G266600 | AAP1 | 2B | 359130524 | Sdr |
| TRAESCS2B02G273400 | TRAESCS2B02G273400 | 2B | 374685224 | Cr |
| TRAESCS2B02G275600 | TRAESCS2B02G275600 | 2B | 380308325 | Cr |
| TRAESCS2B02G274400 | TRAESCS2B02G274400 | 2B | 377526036 | Sdr |
| TRAESCS2B02G248500 | TRAESCS2B02G248500 | 2B | 254786750 | Cr |
| TRAESCS2B02G256300 | TRAESCS2B02G256300 | 2B | 298151055 | Cr |
| TRAESCS2B02G259100 | CI51 | 2B | 320738984 | Sdr |
| TRAESCS2B02G269200 | CPK17 | 2B | 364729261 | Cr |
| TRAESCS2B02G246800 | TRAESCS2B02G246800 | 2B | 250901986 | Cr |
| TRAESCS2B02G259700 | TRAESCS2B02G259700 | 2B | 324794055 | Cr |
| TRAESCS2B02G250400 | FPP | 2B | 259781668 | Sdr |
| TRAESCS2B02G261500 | TRAESCS2B02G261500 | 2B | 335493444 | Sdr |
| TRAESCS2B02G265300 | ATP9 | 2B | 357675575 | Sdr |
| TRAESCS2B02G259300 | TRAESCS2B02G259300 | 2B | 321690002 | Sdr |
| TRAESCS2B02G254400 | TRAESCS2B02G254400 | 2B | 283013461 | Sdr |
| TRAESCS2B02G267800 | TRAESCS2B02G267800 | 2B | 360981446 | Sdr |
| TRAESCS2B02G274300 | TRAESCS2B02G274300 | 2B | 377334561 | Sdr |
| TRAESCS2B02G261000 | FTIP7 | 2B | 331228711 | Sdr |
| TRAESCS2B02G250100 | PPC3-1.2 | 2B | 259364568 | Sdr |
| TRAESCS2B02G255100 | TRAESCS2B02G255100 | 2B | 288936508 | Cr |
| TRAESCS2B02G252200 | PNG1 | 2B | 269360367 | Sdr |
| TRAESCS2B02G254800 | 2KGR | 2B | 286508203 | Cr |
| TRAESCS2B02G270000 | TRAESCS2B02G270000 | 2B | 368303722 | Sdr |
| TRAESCS2B02G263800 | TRAESCS2B02G263800 | 2B | 355885467 | Sdr |
| TRAESCS2B02G252900 | NUP54 | 2B | 272756410 | Sdr |
| TRAESCS2B02G247900 | TRAESCS2B02G247900 | 2B | 254621790 | Sdr |
| TRAESCS2B02G245900 | TRAESCS2B02G245900 | 2B | 250065530 | Sdr |
| TRAESCS2B02G274700 | TRAESCS2B02G274700 | 2B | 378435090 | Sdr |
| TRAESCS2B02G257400 | TRAESCS2B02G257400 | 2B | 308714375 | Cr |
| TRAESCS2B02G262100 | TRAESCS2B02G262100 | 2B | 339108410 | Sdr |
| TRAESCS2B02G252000 | MYB2 | 2B | 266961015 | Cr |
| TRAESCS2B02G273000 | TRAESCS2B02G273000 | 2B | 374239120 | Cr |
| TRAESCS2B02G268600 | TRAESCS2B02G268600 | 2B | 363486580 | Cr |

Cr=Circadian clocking, FL=Flowering, Sdr=Seed dormancy, Vr=Vernalization

**Table S3.** Passport information of the accessions and ancestral coefficients (Q1 and Q2) based on the unique 2B region from genome scan of WG resequencing data

| **Accession** | **Acc_ID** | **Subspecies** | **Common name** | **Latitude** | **Longitude** | **Q1** | **Q2** | **Group** |
| --- | --- | --- | --- | --- | --- | --- | --- | --- |
| HUN_Ttur_Riv | B002 | *Triticum turgidum* L. ssp. turgidum | Rivet wheat | 47.41667 | 19.33333 | 0.0001 | 0.9999 | Group1 |
| GEO_Ttur_Riv | B008 | *Triticum turgidum* L. ssp. turgidum | Rivet wheat | 41.71667 | 44.81667 | 0.0001 | 0.9999 | Group1 |
| IRN_Ttur_Riv | B009 | *Triticum turgidum* L. ssp. turgidum | Rivet wheat | 38 | 45 | 0.0001 | 0.9999 | Group1 |
| AFG_Ttur_Kho | B056 | *Triticum turgidum* L. ssp. turanicum (Jakubz.) A. Love & D. Love | Khorasan wheat | 34.345 | 62.19972 | 0.0001 | 0.9999 | Group1 |
| ESP_Ttur_Dom | B064 | *Triticum turgidum* L. ssp. dicoccum (Schrank) Thell. | Domesticated Emmer | 43.36667 | -5.83333 | 0.0001 | 0.9999 | Group1 |
| GBR_Ttur_Dom | B076 | *Triticum turgidum* L. ssp. dicoccum (Schrank) Thell. | Domesticated emmer | 53 | -2 | 0.0001 | 0.9999 | Group1 |
| CHN_Ttur_Per | B093 | *Triticum turgidum* L. ssp. carthlicum (Nevski) A. Love & D. Love | Persian wheat | 45.75 | 126.65 | 0.0001 | 0.9999 | Group1 |
| HUN_Ttur_Per | B094 | *Triticum turgidum* L. ssp. carthlicum (Nevski) A. Love & D. Love | Persian wheat | 47.41667 | 19.33333 | 0.0001 | 0.9999 | Group1 |
| TUR_Ttur_Per | B096 | *Triticum turgidum* L. ssp. carthlicum (Nevski) A. Love & D. Love | Persian wheat | 41.13333 | 42.91667 | 0.0001 | 0.9999 | Group1 |
| GEO_Ttur_Per | B097 | *Triticum turgidum* L. ssp. carthlicum (Nevski) A. Love & D. Love | Persian wheat | 41.71667 | 44.78333 | 0.0001 | 0.9999 | Group1 |
| GEO_Ttur_Per | B098 | *Triticum turgidum* L. ssp. carthlicum (Nevski) A. Love & D. Love | Persian wheat | 41.71667 | 44.78333 | 0.0001 | 0.9999 | Group1 |
| TUR_Ttur_Per | B099 | *Triticum turgidum* L. ssp. carthlicum (Nevski) A. Love & D. Love | Persian wheat | 40.88333 | 43.26667 | 0.0001 | 0.9999 | Group1 |
| GEO_Ttur_Per | B100 | *Triticum turgidum* L. ssp. carthlicum (Nevski) A. Love & D. Love | Persian wheat | 41.71667 | 44.78333 | 0.0001 | 0.9999 | Group1 |
| GEO_Tkar_Geo | B110 | Triticum karamyschevii NEVSKI var. karamyschevii | Georgian wheat | 41.62667 | 44.68333 | 0.0001 | 0.9999 | Group1 |
| GEO_Tkar_Geo | B111 | Triticum karamyschevii NEVSKI var. karamyschevii | Georgian wheat | 41.71667 | 44.88333 | 0.0001 | 0.9999 | Group1 |
| GEO_Tkar_Geo | B112 | Triticum karamyschevii NEVSKI var. karamyschevii | Georgian wheat | 41.62667 | 44.80333 | 0.0001 | 0.9999 | Group1 |
| HUN_Ttur_Riv | B004 | *Triticum turgidum* L. ssp. turgidum | Rivet wheat | 47.41667 | 19.33333 | 0.0013 | 0.9987 | Group1 |
| TUR_Ttur_Per | B101 | *Triticum turgidum* L. ssp. carthlicum (Nevski) A. Love & D. Love | Persian wheat | 40.91667 | 42.61667 | 0.0021 | 0.9980 | Group1 |
| GEO_Ttur_Per | B102 | *Triticum turgidum* L. ssp. carthlicum (Nevski) A. Love & D. Love | Persian wheat | 43 | 42.61667 | 0.0032 | 0.9968 | Group1 |
| TUR_Ttur_Kho | B061 | *Triticum turgidum* L. ssp. turanicum (Jakubz.) A. Love & D. Love | Khorasan wheat | 37.45056 | 35.81639 | 0.0032 | 0.9968 | Group1 |
| ISR_Ttur_Wil | B040 | *Triticum turgidum* L. ssp. dicoccoides (Korn. ex Asch. & Graebn.) Thell. | Wild emmer | 31.8 | 35.03333 | 0.0063 | 0.9937 | Group1 |
| SYR_Ttur_Wil | B027 | *Triticum turgidum* L. ssp. dicoccoides (Korn. ex Asch. & Graebn.) Thell. | Wild emmer | 32.86944 | 36.03083 | 0.9978 | 0.0022 | Group2 |
| HUN_Ttur_Kho | B054 | *Triticum turgidum* L. ssp. turanicum (Jakubz.) A. Love & D. Love | Khorasan wheat | 47.41667 | 19.33333 | 0.9982 | 0.0018 | Group2 |
| LBN_Ttur_Wil | B034 | *Triticum turgidum* L. ssp. dicoccoides (Korn. ex Asch. & Graebn.) Thell. | Wild emmer | 33.51667 | 35.86667 | 0.9984 | 0.0016 | Group2 |
| ETH_Ttur_Dom | B082 | *Triticum turgidum* L. ssp. dicoccum (Schrank) Thell. | Domesticated emmer | 9.116667 | 38.38333 | 0.9994 | 0.0006 | Group2 |
| TUR_Ttur_Riv | B001 | *Triticum turgidum* L. ssp. turgidum | Rivet wheat | 40 | 41.5 | 0.9999 | 0.0001 | Group2 |
| ETH_Ttur_Riv | B003 | *Triticum turgidum* L. ssp. turgidum | Rivet wheat | 9.033333 | 38.7 | 0.9999 | 0.0001 | Group2 |
| MAC_Ttur_Riv | B005 | *Triticum turgidum* L. ssp. turgidum | Rivet wheat | 41.16667 | 20.83333 | 0.9999 | 0.0001 | Group2 |
| MAC_Ttur_Riv | B006 | *Triticum turgidum* L. ssp. turgidum | Rivet wheat | 41.55222 | 21.97639 | 0.9999 | 0.0001 | Group2 |
| TUR_Ttur_Riv | B007 | *Triticum turgidum* L. ssp. turgidum | Rivet wheat | 41.21917 | 31.95583 | 0.9999 | 0.0001 | Group2 |
| TUR_Ttur_Riv | B010 | *Triticum turgidum* L. ssp. turgidum | Rivet wheat | 38.18333 | 36.5 | 0.9999 | 0.0001 | Group2 |
| TUR_Ttur_Riv | B011 | *Triticum turgidum* L. ssp. turgidum | Rivet wheat | 38 | 37 | 0.9999 | 0.0001 | Group2 |
| EGY_Ttur_Riv | B012 | *Triticum turgidum* L. ssp. turgidum | Rivet wheat | 29.5 | 34 | 0.9999 | 0.0001 | Group2 |
| ETH_Ttur_Pol | B013 | *Triticum turgidum* L. ssp. polonicum (L.) Thell. | Polish wheat | 8.966667 | 37.65 | 0.9999 | 0.0001 | Group2 |
| PRT_Ttur_Pol | B014 | *Triticum turgidum* L. ssp. polonicum (L.) Thell. | Polish wheat | 40.61667 | -8.41667 | 0.9999 | 0.0001 | Group2 |
| HUN_Ttur_Pol | B015 | *Triticum turgidum* L. ssp. polonicum (L.) Thell. | Polish wheat | 47.41667 | 19.33333 | 0.9999 | 0.0001 | Group2 |
| ETH_Ttur_Pol | B016 | *Triticum turgidum* L. ssp. polonicum (L.) Thell. | Polish wheat | 8.533333 | 37.98333 | 0.9999 | 0.0001 | Group2 |
| ETH_Ttur_Pol | B017 | *Triticum turgidum* L. ssp. polonicum (L.) Thell. | Polish wheat | 8.966667 | 37.65 | 0.9999 | 0.0001 | Group2 |
| IND_Ttur_Pol | B018 | *Triticum turgidum* L. ssp. polonicum (L.) Thell. | Polish wheat | 24.58333 | 73.68333 | 0.9999 | 0.0001 | Group2 |
| ETH_Ttur_Pol | B019 | *Triticum turgidum* L. ssp. polonicum (L.) Thell. | Polish wheat | 8.966667 | 37.65 | 0.9999 | 0.0001 | Group2 |
| HUN_Ttur_Pol | B020 | *Triticum turgidum* L. ssp. polonicum (L.) Thell. | Polish wheat | 47.41667 | 19.33333 | 0.9999 | 0.0001 | Group2 |
| TUR_Ttur_Pol | B021 | *Triticum turgidum* L. ssp. polonicum (L.) Thell. | Polish wheat | 39.91361 | 28.15778 | 0.9999 | 0.0001 | Group2 |
| PRT_Ttur_Pol | B022 | *Triticum turgidum* L. ssp. polonicum (L.) Thell. | Polish wheat | 40.61667 | -8.41667 | 0.9999 | 0.0001 | Group2 |
| IRN_Ttur_Wil | B023 | *Triticum turgidum* L. ssp. dicoccoides (Korn. ex Asch. & Graebn.) Thell. | Wild emmer | 34.36667 | 46.1 | 0.9999 | 0.0001 | Group2 |
| SYR_Ttur_Wil | B024 | *Triticum turgidum* L. ssp. dicoccoides (Korn. ex Asch. & Graebn.) Thell. | Wild emmer | 32.99 | 35.69 | 0.9999 | 0.0001 | Group2 |
| ISR_Ttur_Wil | B025 | *Triticum turgidum* L. ssp. dicoccoides (Korn. ex Asch. & Graebn.) Thell. | Wild emmer | 32.59667 | 35.00194 | 0.9999 | 0.0001 | Group2 |
| ISR_Ttur_Wil | B026 | *Triticum turgidum* L. ssp. dicoccoides (Korn. ex Asch. & Graebn.) Thell. | Wild emmer | 31.95 | 35.33333 | 0.9999 | 0.0001 | Group2 |
| ISR_Ttur_Wil | B028 | *Triticum turgidum* L. ssp. dicoccoides (Korn. ex Asch. & Graebn.) Thell. | Wild emmer | 32.59667 | 35.00194 | 0.9999 | 0.0001 | Group2 |
| ISR_Ttur_Wil | B029 | *Triticum turgidum* L. ssp. dicoccoides (Korn. ex Asch. & Graebn.) Thell. | Wild emmer | 32.96667 | 35.53333 | 0.9999 | 0.0001 | Group2 |
| SYR_Ttur_Wil | B030 | *Triticum turgidum* L. ssp. dicoccoides (Korn. ex Asch. & Graebn.) Thell. | Wild emmer | 32.99 | 35.69 | 0.9999 | 0.0001 | Group2 |
| LBN_Ttur_Wil | B031 | *Triticum turgidum* L. ssp. dicoccoides (Korn. ex Asch. & Graebn.) Thell. | Wild emmer | 33.51667 | 35.86667 | 0.9999 | 0.0001 | Group2 |
| ISR_Ttur_Wil | B032 | *Triticum turgidum* L. ssp. dicoccoides (Korn. ex Asch. & Graebn.) Thell. | Wild emmer | 32.9 | 35.38333 | 0.9999 | 0.0001 | Group2 |
| TUR_Ttur_Wil | B033 | *Triticum turgidum* L. ssp. dicoccoides (Korn. ex Asch. & Graebn.) Thell. | Wild emmer | 37.88333 | 39.86667 | 0.9999 | 0.0001 | Group2 |
| LBN_Ttur_Wil | B035 | *Triticum turgidum* L. ssp. dicoccoides (Korn. ex Asch. & Graebn.) Thell. | Wild emmer | 33.53667 | 35.86667 | 0.9999 | 0.0001 | Group2 |
| ISR_Ttur_Wil | B036 | *Triticum turgidum* L. ssp. dicoccoides (Korn. ex Asch. & Graebn.) Thell. | Wild emmer | 32.96667 | 35.53333 | 0.9999 | 0.0001 | Group2 |
| SYR_Ttur_Wil | B037 | *Triticum turgidum* L. ssp. dicoccoides (Korn. ex Asch. & Graebn.) Thell. | Wild emmer | 32.64778 | 36.79 | 0.9999 | 0.0001 | Group2 |
| TUR_Ttur_Wil | B038 | *Triticum turgidum* L. ssp. dicoccoides (Korn. ex Asch. & Graebn.) Thell. | Wild emmer | 37.86667 | 39.88333 | 0.9999 | 0.0001 | Group2 |
| ISR_Ttur_Wil | B039 | *Triticum turgidum* L. ssp. dicoccoides (Korn. ex Asch. & Graebn.) Thell. | Wild emmer | 32.59667 | 35.00194 | 0.9999 | 0.0001 | Group2 |
| TUR_Ttur_Wil | B041 | *Triticum turgidum* L. ssp. dicoccoides (Korn. ex Asch. & Graebn.) Thell. | Wild emmer | 37.88333 | 39.86667 | 0.9999 | 0.0001 | Group2 |
| LBN_Ttur_Wil | B042 | *Triticum turgidum* L. ssp. dicoccoides (Korn. ex Asch. & Graebn.) Thell. | Wild emmer | 33.50111 | 35.83944 | 0.9999 | 0.0001 | Group2 |
| ISR_Ttur_Wil | B044 | *Triticum turgidum* L. ssp. dicoccoides (Korn. ex Asch. & Graebn.) Thell. | Wild emmer | 32.96667 | 35.53333 | 0.9999 | 0.0001 | Group2 |
| TUR_Ttur_Wil | B045 | *Triticum turgidum* L. ssp. dicoccoides (Korn. ex Asch. & Graebn.) Thell. | Wild emmer | 37.88333 | 39.86667 | 0.9999 | 0.0001 | Group2 |
| TUR_Ttur_Wil | B046 | *Triticum turgidum* L. ssp. dicoccoides (Korn. ex Asch. & Graebn.) Thell. | Wild emmer | 37.78333 | 39.76667 | 0.9999 | 0.0001 | Group2 |
| ISR_Ttur_Wil | B047 | *Triticum turgidum* L. ssp. dicoccoides (Korn. ex Asch. & Graebn.) Thell. | Wild emmer | 31.8 | 35.03333 | 0.9999 | 0.0001 | Group2 |
| ISR_Ttur_Wil | B048 | *Triticum turgidum* L. ssp. dicoccoides (Korn. ex Asch. & Graebn.) Thell. | Wild emmer | 32.96667 | 35.53333 | 0.9999 | 0.0001 | Group2 |
| SYR_Ttur_Wil | B049 | *Triticum turgidum* L. ssp. dicoccoides (Korn. ex Asch. & Graebn.) Thell. | Wild emmer | 32.99 | 35.69 | 0.9999 | 0.0001 | Group2 |
| TUR_Ttur_Wil | B050 | *Triticum turgidum* L. ssp. dicoccoides (Korn. ex Asch. & Graebn.) Thell. | Wild emmer | 37.88333 | 39.86667 | 0.9999 | 0.0001 | Group2 |
| SYR_Ttur_Wil | B052 | *Triticum turgidum* L. ssp. dicoccoides (Korn. ex Asch. & Graebn.) Thell. | Wild emmer | 32.99 | 35.69 | 0.9999 | 0.0001 | Group2 |
| AFG_Ttur_Kho | B053 | *Triticum turgidum* L. ssp. turanicum (Jakubz.) A. Love & D. Love | Khorasan wheat | 34.51667 | 65.13333 | 0.9999 | 0.0001 | Group2 |
| PRT_Ttur_Kho | B055 | *Triticum turgidum* L. ssp. turanicum (Jakubz.) A. Love & D. Love | Khorasan wheat | 40.61667 | -8.41667 | 0.9999 | 0.0001 | Group2 |
| IRN_Ttur_Kho | B057 | *Triticum turgidum* L. ssp. turanicum (Jakubz.) A. Love & D. Love | Khorasan wheat | 34.31667 | 47.06667 | 0.9999 | 0.0001 | Group2 |
| IRN_Ttur_Kho | B058 | *Triticum turgidum* L. ssp. turanicum (Jakubz.) A. Love & D. Love | Khorasan wheat | 34.31667 | 47.06667 | 0.9999 | 0.0001 | Group2 |
| IRN_Ttur_Kho | B059 | *Triticum turgidum* L. ssp. turanicum (Jakubz.) A. Love & D. Love | Khorasan wheat | 35.86667 | 47.81667 | 0.9999 | 0.0001 | Group2 |
| TUR_Ttur_Kho | B060 | *Triticum turgidum* L. ssp. turanicum (Jakubz.) A. Love & D. Love | Khorasan wheat | 40.75 | 33.41667 | 0.9999 | 0.0001 | Group2 |
| TUR_Ttur_Kho | B062 | *Triticum turgidum* L. ssp. turanicum (Jakubz.) A. Love & D. Love | Khorasan wheat | 37.73333 | 38.66667 | 0.9999 | 0.0001 | Group2 |
| ETH_Ttur_Dom | B063 | *Triticum turgidum* L. ssp. dicoccum (Schrank) Thell. | Domesticated emmer | 9.2 | 38.6 | 0.9999 | 0.0001 | Group2 |
| ETH_Ttur_Dom | B065 | *Triticum turgidum* L. ssp. dicoccum (Schrank) Thell. | Domesticated emmer | 9.316667 | 42.11667 | 0.9999 | 0.0001 | Group2 |
| ETH_Ttur_Dom | B066 | *Triticum turgidum* L. ssp. dicoccum (Schrank) Thell. | Domesticated emmer | 13.48333 | 39.55 | 0.9999 | 0.0001 | Group2 |
| OMN_Ttur_Dom | B067 | *Triticum turgidum* L. ssp. dicoccum (Schrank) Thell. | Domesticated emmer | 23.83333 | 56.33333 | 0.9999 | 0.0001 | Group2 |
| ETH_Ttur_Dom | B068 | *Triticum turgidum* L. ssp. dicoccum (Schrank) Thell. | Domesticated emmer | 23.16667 | 57.66667 | 0.9999 | 0.0001 | Group2 |
| ETH_Ttur_Dom | B069 | *Triticum turgidum* L. ssp. dicoccum (Schrank) Thell. | Domesticated emmer | 9.316667 | 42.11667 | 0.9999 | 0.0001 | Group2 |
| ETH_Ttur_Dom | B070 | *Triticum turgidum* L. ssp. dicoccum (Schrank) Thell. | Domesticated emmer | 9.166667 | 38.81667 | 0.9999 | 0.0001 | Group2 |
| ETH_Ttur_Dom | B071 | *Triticum turgidum* L. ssp. dicoccum (Schrank) Thell. | Domesticated emmer | 8.866667 | 38.78333 | 0.9999 | 0.0001 | Group2 |
| SRB_Ttur_Dom | B072 | *Triticum turgidum* L. ssp. dicoccum (Schrank) Thell. | Domesticated emmer | 44.83333 | 20.5 | 0.9999 | 0.0001 | Group2 |
| ETH_Ttur_Dom | B073 | *Triticum turgidum* L. ssp. dicoccum (Schrank) Thell. | Domesticated emmer | 9.316667 | 42.11667 | 0.9999 | 0.0001 | Group2 |
| ETH_Ttur_Dom | B074 | *Triticum turgidum* L. ssp. dicoccum (Schrank) Thell. | Domesticated emmer | 8.733333 | 38.98333 | 0.9999 | 0.0001 | Group2 |
| ETH_Ttur_Dom | B075 | *Triticum turgidum* L. ssp. dicoccum (Schrank) Thell. | Domesticated emmer | 9.316667 | 42.11667 | 0.9999 | 0.0001 | Group2 |
| ETH_Ttur_Dom | B077 | *Triticum turgidum* L. ssp. dicoccum (Schrank) Thell. | Domesticated emmer | 11.5 | 40 | 0.9999 | 0.0001 | Group2 |
| USA_Ttur_Dom | B078 | *Triticum turgidum* L. ssp. dicoccum (Schrank) Thell. | Domesticated emmer | 46 | -94 | 0.9999 | 0.0001 | Group2 |
| ETH_Ttur_Dom | B079 | *Triticum turgidum* L. ssp. dicoccum (Schrank) Thell. | Domesticated emmer | 9.2 | 38.6 | 0.9999 | 0.0001 | Group2 |
| SRB_Ttur_Dom | B080 | *Triticum turgidum* L. ssp. dicoccum (Schrank) Thell. | Domesticated emmer | 44.83333 | 20.5 | 0.9999 | 0.0001 | Group2 |
| IRN_Ttur_Dom | B081 | *Triticum turgidum* L. ssp. dicoccum (Schrank) Thell. | Domesticated emmer | 32.68333 | 51.68333 | 0.9999 | 0.0001 | Group2 |
| Mon_Ttur_Dom | B083 | *Triticum turgidum* L. ssp. dicoccum (Schrank) Thell. | Domesticated emmer | 42.35 | 19.3 | 0.9999 | 0.0001 | Group2 |
| RUS_Ttur_Dom | B084 | *Triticum turgidum* L. ssp. dicoccum (Schrank) Thell. | Domesticated emmer | 42 | 47 | 0.9999 | 0.0001 | Group2 |
| RUS_Ttur_Dom | B085 | *Triticum turgidum* L. ssp. dicoccum (Schrank) Thell. | Domesticated emmer | 42 | 47 | 0.9999 | 0.0001 | Group2 |
| RUS_Ttur_Dom | B086 | *Triticum turgidum* L. ssp. dicoccum (Schrank) Thell. | Domesticated emmer | 43 | 47 | 0.9999 | 0.0001 | Group2 |
| RUS_Ttur_Dom | B087 | *Triticum turgidum* L. ssp. dicoccum (Schrank) Thell. | Domesticated emmer | 42 | 47 | 0.9999 | 0.0001 | Group2 |
| ETH_Ttur_Dom | B088 | *Triticum turgidum* L. ssp. dicoccum (Schrank) Thell. | Domesticated emmer | 8.6 | 39.11667 | 0.9999 | 0.0001 | Group2 |
| IRN_Ttur_Dom | B089 | *Triticum turgidum* L. ssp. dicoccum (Schrank) Thell. | Domesticated emmer | 36.23333 | 46.26667 | 0.9999 | 0.0001 | Group2 |
| Arm_Ttur_Dom | B090 | *Triticum turgidum* L. ssp. dicoccum (Schrank) Thell. | Domesticated emmer | 40.18333 | 44.5 | 0.9999 | 0.0001 | Group2 |
| IRN_Ttur_Dom | B091 | *Triticum turgidum* L. ssp. dicoccum (Schrank) Thell. | Domesticated emmer | 36.23333 | 46.26667 | 0.9999 | 0.0001 | Group2 |
| IRN_Tisp_Isp | B103 | Triticum ispahanicum Heslot | Ispahanicum | 36.23333 | 46.29667 | 0.9999 | 0.0001 | Group2 |
| FRA_Tisp_Isp | B104 | Triticum ispahanicum Heslot | Ispahanicum | 45.7772 | 3.087 | 0.9999 | 0.0001 | Group2 |
| IRN_Tisp_Isp | B105 | Triticum ispahanicum Heslot | Ispahanicum | 36.23333 | 46.26667 | 0.9999 | 0.0001 | Group2 |
| FRA_Tisp_Isp | B106 | Triticum ispahanicum Heslot | Ispahanicum | 45.7772 | 3.117 | 0.9999 | 0.0001 | Group2 |
| IRN_Tisp_Isp | B107 | Triticum ispahanicum Heslot | Ispahanicum | 32.68333 | 51.68333 | 0.9999 | 0.0001 | Group2 |
| IRN_Tisp_Isp | B108 | Triticum ispahanicum Heslot | Ispahanicum | 36.23333 | 46.26667 | 0.9999 | 0.0001 | Group2 |
| IRN_Tisp_Isp | B109 | Triticum ispahanicum Heslot | Ispahanicum | 33.56667 | 50.85 | 0.9999 | 0.0001 | Group2 |
| TUR_Ttur_Dur | B113 | *Triticum turgidum* L. ssp. durum (Desf.) Husn. | Durum | 40.79778 | 29.43056 | 0.9999 | 0.0001 | Group2 |
| TUR_Ttur_Dur | B114 | *Triticum turgidum* L. ssp. durum (Desf.) Husn. | Durum | 37.95 | 58.33333 | 0.9999 | 0.0001 | Group2 |
| IRN_Ttur_Dur | B115 | *Triticum turgidum* L. ssp. durum (Desf.) Husn. | Durum | 34.31667 | 47.06667 | 0.9999 | 0.0001 | Group2 |
| IRN_Ttur_Dur | B116 | *Triticum turgidum* L. ssp. durum (Desf.) Husn. | Durum | 32.31667 | 50.83333 | 0.9999 | 0.0001 | Group2 |
| IRN_Ttur_Dur | B117 | *Triticum turgidum* L. ssp. durum (Desf.) Husn. | Durum | 37 | 49 | 0.9999 | 0.0001 | Group2 |
| IRN_Ttur_Dur | B118 | *Triticum turgidum* L. ssp. durum (Desf.) Husn. | Durum | 34.13333 | 45.91667 | 0.9999 | 0.0001 | Group2 |
| IRN_Ttur_Dur | B119 | *Triticum turgidum* L. ssp. durum (Desf.) Husn. | Durum | 34.31667 | 47.06667 | 0.9999 | 0.0001 | Group2 |
| IRN_Ttur_Dur | B120 | *Triticum turgidum* L. ssp. durum (Desf.) Husn. | Durum | 32.68333 | 51.68333 | 0.9999 | 0.0001 | Group2 |
| IRN_Ttur_Dur | B121 | *Triticum turgidum* L. ssp. durum (Desf.) Husn. | Durum | 34.11167 | 46.52833 | 0.9999 | 0.0001 | Group2 |
| KAZ_Ttur_Dur | B122 | *Triticum turgidum* L. ssp. durum (Desf.) Husn. | Durum | 51.8 | 68.33333 | 0.9999 | 0.0001 | Group2 |
| IRN_Ttur_Dur | B123 | *Triticum turgidum* L. ssp. durum (Desf.) Husn. | Durum | 34.31667 | 47.06667 | 0.9999 | 0.0001 | Group2 |
| TUR_Ttur_Dur | B124 | *Triticum turgidum* L. ssp. durum (Desf.) Husn. | Durum | 37.02944 | 37.99028 | 0.9999 | 0.0001 | Group2 |
| PRT_Ttur_Dur | B125 | *Triticum turgidum* L. ssp. durum (Desf.) Husn. | Durum | 39 | -9.13333 | 0.9999 | 0.0001 | Group2 |

Passport information was extracted from (Zhou et al. 2020), each color shade indicates accessions sharing over 98% of their ancestral proportion.

Zhou Y, Zhao X, Li Y, Xu J, Bi A, Kang L, Xu D, Chen H, Wang Y, Wang Y-g (2020) Triticum population sequencing provides insights into wheat adaptation. Nature genetics 52:1412-1422
